# A phase II trial of weekly nab-paclitaxel for progressive and symptomatic desmoid tumors

**DOI:** 10.1038/s41467-022-33975-6

**Published:** 2022-10-21

**Authors:** Javier Martin-Broto, Andres Redondo, David S. Moura, Claudia Valverde, Jose Manuel Morales, Antonio Lopez-Pousa, Javier Martinez-Trufero, Antonio Gutierrez, Roberto Díaz-Beveridge, Pablo Luna, Virginia Martinez-Marin, David Marcilla, Ivan Arribas, Patricio Ledesma, Jose Antonio Lopez-Martin, Davide Di Lernia, Jorge Zamora, Nadia Hindi

**Affiliations:** 1 Health Research Institute-Fundación Jiménez Díaz University Hospital, Universidad Autónoma de Madrid (IIS-FJD, UAM), 28040 Madrid, Spain; 2 Medical Oncology Department, Fundación Jimenez Diaz University Hospital, 28040 Madrid, Spain; 3 General de Villalba University Hospital, 28400 Madrid, Spain; 4 Autonomous University of Madrid, 28049 Madrid, Spain; 5 Department of Medical Oncology, Hospital Universitario La Paz, 28046 Madrid, Spain; 6 Department of Medical Oncology, Vall d’Hebron University Hospital, 08035 Barcelona, Spain; 7 Radiology Department, Virgen del Rocio University Hospital, 41013 Sevilla, Spain; 8 Medical Oncology Department, Sant Pau Hospital, 08025 Barcelona, Spain; 9 Medical Oncology Department, Miguel Servet University Hospital, 50009 Zaragoza, Spain; 10 Hematology Department, University Hospital Son Espases, 07120 Mallorca, Spain; 11 Medical Oncology Department, Hospital Universitari i Politècnic La Fe, 46026 Valencia, Spain; 12 Medical Oncology Department, Son Espases University Hospital, 07120 Mallorca, Spain; 13 Pathology Department, Virgen del Rocio University Hospital, 41013 Sevilla, Spain; 14 Universitat de València, 46010 Valencia, Spain; 15 Sofpromed Investigacion Clinica SLU, 07009 Mallorca, Spain; 16 Medical Oncology Department, 12 de Octubre University Hospital, 28041 Madrid, Spain

**Keywords:** Sarcoma, Phase II trials

## Abstract

Desmoid fibromatosis (DF) are mesenchymal neoplasms, with potential aggressive course and relevant clinical impact. New systemic therapy modalities are needed in this symptomatic/progressive population. In this multicenter, phase II trial (NCT03275818), patients with symptomatic/progressing DF received three cycles of weekly nab-paclitaxel. Brief pain inventory short form (BPI-SF) was collected at baseline and in every visit. MRI was performed every 3 months. Primary composite endpoint was RECIST 1.1 overall response rate (ORR) and/or clinical response (improvement ≥ 2 points in BPI-SF). If 40% of patients achieved clinical/radiological response, further investigation would be warranted. Toxicity, progression-free survival (PFS), pattern of response and its correlation with clinical best response and BPI, variation of physical function, and analgesic consumption were secondary endpoints. The translational research reported was not a pre-specified secondary outcome. Forty eligible patients started therapy, being 35 radiologically and clinically evaluable. The study achieved its primary endpoint, as 7(20%) patients obtained RECIST partial response, whereas 31(89%) experienced pain reduction of ≥2 points in BPI-SF worst pain. Therapy was well tolerated. With a median follow-up of 30(14–44) months, median 12 and 24-months PFS rates were 91%(CI 95%, 82–100) and 84%(CI 95%, 71–97). For clinical progression, 12 and 24-months PFS rates were 85% (CI 95%, 73–97) and 74% (CI 95%, 58–90) respectively. Short course of nab-paclitaxel is active, safe and achieves quick and durable responses in progressing/symptomatic DF patients.

## Abstract (web summary)

In patients with progressive and symptomatic desmoid fibromatosis (DF) treatment with chemotherapy is typically prolonged, while surgery carries a high risk of relapse. Here, the authors report the results of a phase II clinical trial of short-course nab-paclitaxel in patients with progressive and symptomatic DF.

## Introduction

Desmoid fibromatosis (DF) constitutes a ubiquitous mesenchymal clonal neoplasia demonstrating a fibroblastic/myofibroblastic proliferation in long fascicles, sometimes with notorious collagen fibers, with infiltrating local growth but a null metastatic potential^1^. This intriguing tumor may exhibit different behaviors, from indolent subtle growth to fast aggressive infiltration or even to spontaneous regression. In general, 50% of DF tumors show a pauci-symptomatic comportment, and this has been more evident since the wait-and-see policies have become widespread^2^.

The paradigm switch from surgery as cornerstone to a non-interventional preferred approach in DF is related to several factors. On the one hand, large surgical series from sarcoma expert centers reported worrisome figures, with 5-year relapse-free survival ranging from 52.8%^3^ to 69%^4^, or an ~90% incidence of relapse if surgery was offered in recurrent DF^5^. Furthermore, recurrent DF cases usually display a more clinically apparent disease. On the other hand, observational studies reported a similar 3-year event-free survival between conservative vs up-front surgical approaches, 68 vs 65%, respectively^6^. Tumor location has prognostic influence, with the DF of abdominal wall being the most frequently related to spontaneous regression (28.4% in a large series), and the best tolerated for wait-and-see policies^7,8^. At the other extreme, extra-abdominal DF tumors have the highest recurrence rate^3^, especially DF tumors located in girdles or extremities^9^. Furthermore, DF can present as a particularly painful mass or can provoke a functional impairment of an extremity that forces the use of therapy. Likewise, wait-and-see policy is also not recommended if the DF tumor growth is threatening vital structures (such as major vessels, nerves, visceral organs etc.), or in the context of disturbing symptoms.

Of note, DF may frequently induce new symptoms or clinical progression in the absence of RECIST progressive disease (PD)^10^. This singular fact of DF has barely been addressed in studies^11,12^ despite its paramount relevance, since persistent symptoms can determine a poorly perceived quality of life in DF patients. Particularly, pain is the most debilitating symptom reported by DF patients^13^. Substantial symptomatic relief with different treatments is frequently seen in DF patients in the absence of RECIST response, somewhat already prospectively described two decades ago^14^.

The median time to clinical improvement or to response is lacking in most studies; however, this factor is critical to appreciate the added value of a treatment against DF. In prospective studies with systemic treatment, the median time to radiological response has been observed as around 10 months^15–17^. In contrast, the median time to radiological stabilization with a wait-and-see strategy takes longer than 1 year^17,18^, or even more than 2 years^19^, according to different prognostic factors in the series.

The gain-of-function mutation of the *CTNNB1* gene in sporadic DF entails, among other effects, an increase of HIF-1α transcription^20^. Additionally, Wnt pathway activation is related to metabolic remodeling and accentuates the Warburg effect that, ultimately, stimulates neoangiogenesis^21^. Microtubule-targeting drugs, such as taxanes, inhibit angiogenesis through HIF-1α protein translation^22^. These findings provide rationale for the use of taxanes in DF patients. Besides, taxanes can also inhibit metalloproteinase-7 (MMP-7) and vascular endothelial growth factor (VEGF), both targets of beta-catenin and overexpressed in DF.

Among taxanes, nanoparticle albumin-bound paclitaxel (nab-paclitaxel) is a novel formulation of paclitaxel that does not require solvents for its formulation. In breast cancer there is considerable accrued experience with this compound. In the pivotal phase III trial, nab-paclitaxel every 3 weeks, was superior to paclitaxel in terms of response and time to progression.

Our hypothesis is that a low dose and a short course of nab-paclitaxel would be advantageous over other prolonged chemotherapy administrations and might be enough to induce a quick symptomatic response and/or radiological response in measurable and symptomatic DF patients.

Here we show the results of a phase II trial exploring 3-month nab-paclitaxel administration, in this specific subset of symptomatic DF patients.

## Results

### Patients and treatment

**Fig. 1:**
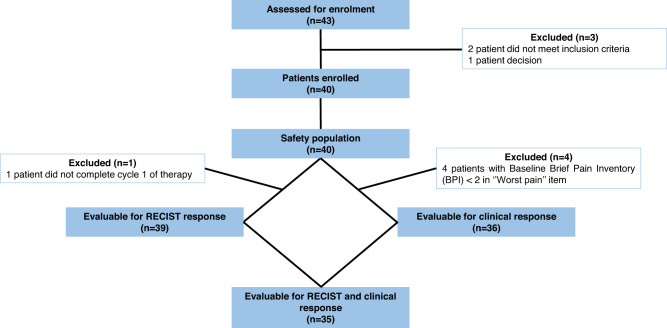
Consort diagram. This figure summarizes the flux of patients in the study.

From May 2017 to September 2019, 43 patients were assessed for eligibility in 8 hospitals belonging to GEIS network. Finally, 40 patients were enrolled in the trial (first and last patient enrolled on 9 May 2017 and 27 September 2019 respectively) as 2 patients did not meet the inclusion criteria and 1 patient refused to be enrolled. The safety population was constituted by these 40 patients since all received at least one nab-paclitaxel administration. There were 39 patients evaluable for RECIST response, one patient only received three nab-paclitaxel doses and then decided to discontinue due to a grade 2 allergic reaction. Four out of the 40 patients did not reach 2-points in the worst pain form of BPI-SF and, as a result, 36 constituted the evaluable population for clinical response. Finally, 35 patients were fully evaluable for both primary endpoints (Fig. 1). The clinical cut-off for the final analyses of data was April 30, 2021. Median dose intensity for nab-paclitaxel in the 35 evaluable patients for the two main endpoints was 100% (33–100%). In this population, the number of patients with dose reductions was 4 (11%), while those with any dose delay or discontinuation numbered 10 (29%).

Demographics and clinical characteristics are listed in Table 1. At entry, there were 13 out of 40 patients (32.5%) with exclusive radiological RECIST progression, 12 out of 40 patients (30%) with exclusive symptomatic progression and 15 out of 40 patients (37.5%) with both radiological and symptomatic progression. Previous surgical resection had been performed in 21 patients (52%), while 10 (25%) had previously received at least one class of systemic treatment including chemotherapy.

**Table 1:** Patient demographics

	*N* = 40
Median age, years (range)	38 (18–76)
Gender (M/F)	14 (35%)/26 (65%)
Reason for inclusion:	
Progression	28 (70%)
Pain	26 (65%)
Functional impairment	20 (50%)
Median time diagnosis to enrollment,	
months (range)	17 (1–187)
Median previous relapses	1 (0–10)
Previous therapy:	23 (57%)
Previous surgery:	21 (52%)
More than 1	7 (17%)
Previous radiotherapy:	5 (12%)
Previous chemotherapy:	6 (15%)
Location:	
Thoracic wall	10 (25%)
Intraabdominal	7 (17%)
Upper extremity	7 (17%)
Lower extremity	7 (17%)
Head and neck	6 (15%)
Abdominal wall	3 (7%)
Location 2:	
High risk (Head and neck	
Or proximal Upper extremity)	11 (27%)
Other	29 (72%)
ECOG BASELINE:	
0	15 (38%)
1	24 (61%)
Extension at baseline:	
Localized	18 (45%)
Locally advanced	22 (55%)
Resectable baseline:	
Resectable	3 (7%)
Unresectable	37 (92%)
Nuclear β-catenin expression	
Positive	39 (98%)
Negative	0 (0%)
Not assessable^a^	1 (2%)
*CTNNB1* mutation:	
T41A	22 (55%)
S45F	3 (8%)
NA	15 (37%)

*M* male, *F* female, *NA* not available.

^a^The diagnosis of this case was confirmed by sequencing *CTNNB1* gene.

### Primary endpoints

Radiological response observed in the subset of 35 patients, evaluable for both main endpoints, was distributed as partial response (PR) in 9 (26%) and stable disease (SD) in 26 (74%) according to local evaluation, whereas following central review, there were 7 PR (20%), 27 SD (77%) and 1 PD (3%). The clinical response in the same population, ≥2-point decrease in worst pain from baseline, was seen in 31 patients (89%). Additionally, ≥4-point and ≥6-point decreases from baseline were seen in 20 (57%) and 11 (31%) patients (Supplementary Table 1).

**Fig. 2:**
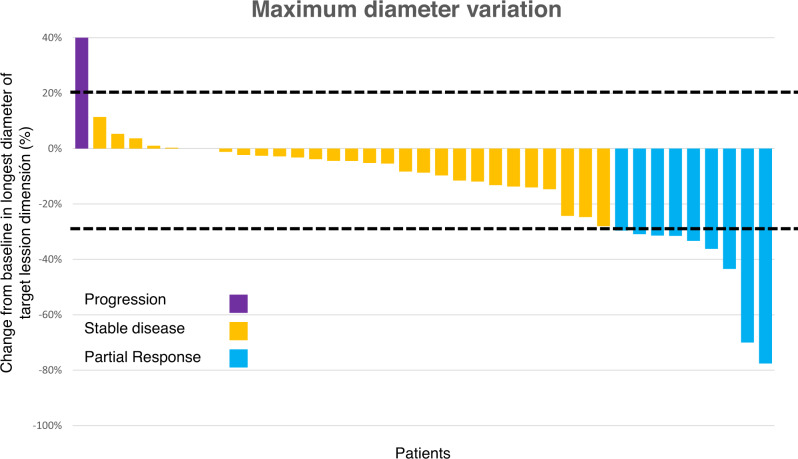
Water fall plot. Among the 39 evaluable patients with evaluable data, the Overall Response Rate (ORR) by RECIST 1.1 was 23%. Lower and upper dashed lines represent the cut-off for progressive disease (an increase of at least 20% in the sum of diameters of target lesions) and for partial response (at least 30% decrease), respectively. Partial response (blue); Stable disease (yellow) and Progression (purple). Source data are provided as a Source Data file.

Regarding the 39 RECIST-evaluable patients, there were 10 (26%) PR and 29 (74%) SD as a result of local assessment, while there were 9 (23%) PR, 29 (74%) SD, and 1 (3%) PD after central radiological assessment (Fig. 2). Remarkably, the median time to obtain radiological PR was 3 (0.5–12.2) and 6.3 (2.3–18.6) months for local and central assessments, respectively. And the median time to obtain a clinical response was 0.9 months (0.2–2.9).

### Secondary endpoints

As listed in Table 2, the proportion of patients that experienced at least one episode of grade 3 or 4 toxicities was exceptionally low. In detail, grade 3 neutropenia was seen in 7.5% of patients, grade 3 mucositis in 2.5% and grade 3 peripheral neuropathy in 2.5%. No grade 4 or 5 toxicities were observed in the trial. The most frequent hematological toxicities were neutropenia (57.5%) and anemia (52.5%). Among non-hematological toxicities, fatigue (55%), alopecia (42.5%), increased alanine aminotransferase (ALT) (37.5%), and diarrhea (35%) were the most frequent treatment emergent adverse effects.

**Table 2:** Toxicity profile (*n* = 40)

Toxicity	Any grade (%)	Grade 1 (%)	Grade 2 (%)	Grade 3 (%)	Grade 4 (%)
Hematological – *n* (%)					
Neutropenia	23 (57.5)	10 (25.0)	10 (25.0)	3 (7.5)	0
Anemia	21 (52.5)	16 (40.0)	5 (12.5)	0	0
Leukopenia	16 (40.0)	13 (32.5)	1 (2.5)	2 (5.0)	0
Thrombocytopenia	2 (5.0)	2 (5.0)	0	0	0
Lymphocytopenia	1 (2.5)	0	1 (2.5)	0	0
Non-hematological – *n* (%)					
Fatigue	22 (55.0)	13 (32.5)	9 (22.5)	0	0
Alopecia	17 (42.5)	3 (7.5)	14 (35.0)	0	0
ALT increased	16 (40.0)	12 (30.0)	4 (10.0)	0	0
Diarrhea	14 (35.0)	11 (27.5)	3 (7.5)	0	0
Neuropathy	14 (35.0)	11 (27.5)	2 (5.0)	1 (2.5)	0
Nausea	13 (32.5)	12 (30.0)	1 (2.5)	0	0
AST increased	10 (25.0)	10 (25.0)	0	0	0
Myalgia/arthralgia	10 (25.0)	7 (17.5)	3 (7.5)	0	0
Hypernatremia	9 (22.5)	9 (22.5)	0	0	0
Mucositis oral	9 (22.5)	8 (20.0)	0	1 (2.5)	0
Hypoglycemia	7 (17.5)	7 (17.5)	0	0	0
Rash and other skin disorders	6 (15.0)	6 (15.0)	0	0	0
Fever	5 (12.5)	5 (12.5)	0	0	0
Headache	5 (12.5)	4 (10.0)	1 (2.5)	0	0
Hyponatremia	5 (12.5)	3 (7.5)	2 (5.0)	0	0
Pruritus	5 (12.5)	4 (10.0)	1 (2.5)	0	0
Constipation	4 (10.0)	4 (10.0)	0	0	0
Dysgeusia	4 (10.0)	3 (7.5)	1 (2.5)	0	0
Nail changes	4 (10.0)	3 (7.5)	1 (2.5)	0	0
Vomiting	4 (10.0)	4 (10.0)	0	0	0
Anorexia	3 (7.5)	2 (5.0)	1 (2.5)	0	0
Dizziness	3 (7.5)	3 (7.5)	0	0	0
Insomnia	3 (7.5)	3 (7.5)	0	0	0
Other gastrointestinal disorders	3 (7.5)	2 (5.0)	1 (2.5)	0	0
Hyperkalemia	2 (5.0)	1 (2.5)	1 (2.5)	0	0
Hypocalcemia	2 (5.0)	1 (2.5)	1 (2.5)	0	0
Hypokalemia	2 (5.0)	2 (5.0)	0	0	0
Localized edema	2 (5.0)	2 (5.0)	0	0	0
Other respiratory disorders	2 (5.0)	2 (5.0)	0	0	0
ALP increased	1 (2.5)	1 (2.5)	0	0	0
Nab-paclitaxel allergic reaction	1 (2.5)	0	1 (2.5)	0	0
Anxiety	1 (2.5)	1 (2.5)	0	0	0
Blood bilirubin increased	1 (2.5)	0	1 (2.5)	0	0
CPK increased	1 (2.5)	1 (2.5)	0	0	0
Epistaxis	1 (2.5)	1 (2.5)	0	0	0
Hypercalcemia	1 (2.5)	1 (2.5)	0	0	0
Hypersomnia	1 (2.5)	1 (2.5)	0	0	0
Hypotension	1 (2.5)	1 (2.5)	0	0	0
Odynophagia	1 (2.5)	1 (2.5)	0	0	0
Pneumonitis	1 (2.5)	0	1 (2.5)	0	0
Vision loss	1 (2.5)	1 (2.5)	0	0	0

*ALT* alanine aminotransferase, *AST* aspartate aminotransferase, *ALP* alkaline phosphatase, *CPK* creatine phosphokinase.

With a median follow-up of 30 (14–44) months, 5 patients (14%) experienced a radiological progression in accordance with central assessment while 8 patients (23%) experienced a clinical progression within the efficacy population (*n* = 35). Neck and proximal upper extremities had significantly worse 30-month PFS rate, 20% compared with 80% in other tumor locations (Supplementary Table 2). The median PFS at 12 and 24 months for RECIST and central review were 91% (CI 95%, 82–100) and 84% (CI 95%, 71–97), respectively in the efficacy population. For clinical progression or increase of at least 2-points in the worst pain questionnaire of BPI-SF, the proportion of patients with PFS rate at 12 and 24 months were 85% (CI 95%, 73–97) and 74% (CI 95%, 58–90), respectively.

Considering analgesic drug consumption, 11 patients (31%) reduced the score by at least one in the AQA, 21 patients (60%) did not change the score and 2 (5%) patients increased the score by one (Supplementary Table 1).

In the univariate analysis, the only two factors with significant favorable prognosis in 30-month PFS rate for RECIST were younger age (≤50 years old) and the achievement of a partial radiological response (Table 3). Univariate analysis for primary endpoint is shown in Supplementary Table 2. The variation of physical function was a pre-specified outcome. However, further secondary endpoint analysis, including this latter, will be presented in a separate article, more focused on quality of life parameters. No other relevant deviations from the planned analysis have been identified.

**Table 3:** Univariate progression-free survival analysis

	Intention-to-treat population (*n* = 40)				Intention-to-treat sub-population (*n* = 35)			
	30 months-central PFS	*P* value	30 months-CR PFS*	*P* value	30 months-central PFS	*P* value	30 months-CR PFS*	*P* value
Median age (years):		0.11		0.057		0.15		0.051
18–38	94% (83–100)		77% (58–97)		93% (79–100)		80% (59–100)	
>38	77% (56–97)		46% (20–73)		76% (55–97)		45% (19–71)	
Age (years):		<0.001		0.25		<0.001		0.33
18–50	93% (83–100)		66% (48–83)		92% (81–100)		65% (46–84)	
>50	57% (20–94)		57% (20–94)		57% (20–94)		57% (20–94)	
Sex (M/F):		0.23		0.093		0.2		0.15
Male	77% (53–100)		48% (21–75)		73% (47–99)		69% (47–91)	
Female	91% (78–100)		72% (52–92)		90% (76–100)		50% (22–78)	
Median time diagnosis to enrollment (range):		0.85		0.93		0.62		0.95
0–17	84% (67–100)		62% (39–84)		81% (61–100)		61% (36–86)	
>17	87% (71–100)		64% (40–88)		87% (70.100)		63% (39–87)	
Previous relapses:		0.37		0.81		0.41		0.9
No	79% (58–100)		65% (40–91)		78% (56–100)		62% (35–89)	
Yes	90% (77–100)		61% (39–83)		89% (74–100)		62% (39–85)	
Previous surgery:		0.64		0.38		0.62		0.58
No	83% (65–100)		73% (53–93)		81% (61–100)		70% (48–92)	
Yes	88% (75–100)		52% (27–77)		87% (70–100)		55% (29–81)	
Previous chemotherapy:		0.37		0.6		0.37		0.44
No	83% (70–97)		63% (45–81)		81% (67–93)		63% (44–82)	
Yes	100% (NA)		60% (17–100)		100% (NA)		50% (1–99)	
Location:		0.15		<0.001		0.16		<0.001
Head and neck & Proximal upper extremity	71% (43–100)		20% (0–51)		71% (43–100)		20% (0–51)	
Other	90% (77–100)		81% (63–98)		90% (77–100)		80% (63–98)	
ECOG baseline:		0.32		0.18		0.22		0.43
0	79% (59–100)		80% (60–100)		74% (49–99)		75% (50–99)	
1	90% (77–100)		50% (27–73)		89% (76–100)		54% (30–77)	
Local response:		0.72		0.28		0.72		0.35
PR	90% (71–100)		80% (55–100)		89% (68–100)		78% (51–100)	
SD	84% (69–99)		56% (36–76)		82% (66–98)		56% (35–78 f)	
Central response:		<0.001		<0.001		<0.001		<0.001
PR	100% (NA)		100% (NA)		100% (NA)		100% (NA)	
SD	84% (69–98)		53% (33–73)		83% (67–98)		54% (33–75)	
PD	0% (NA)		0% (NA)		0% (NA)		0% (NA)	
Change in T2 enhance sequence (MRI)^a^:		0.23		0.13		0.17		0.056
0–7.5	94% (82–100)		79% (57–100)		94% (82–100)		79% (57–100)	
>7.5	77% (57–97)		54% (30–78)		73% (51–96)		46% (20–71)	
New score:		0.63		0.42		0.53		0.25
No change	100% (NA)		67% (13–100)		100% (NA)		67% (13–100)	
0–4	90% (71–100)		80% (55–100)		90% (71–100)		80% (55–100)	
5–9	100% (NA)		100% (NA)		100% (NA)		100% (NA)	
>10	77% (57–97)		54% (30–78)		73% (51–96)		46% (20–71)	
Reduction in pain (BPI worst pain reduction)		0.98		0.73		0.45		0.37
At least −2	87% (65–100)		56% (17–95)		85% (72–99)		50% (1–99)	
<−2	85% (72–99)		65% (47–83)		75% (32–100)		65% (47–83)	

^a^Change in T2: defined as the percentage in changes of the fibrous component of the tumor in T2-enhanced sequence during therapy. Two-sided Fisher’s exact test. Multiple comparisons were not used for this analysis.

*PFS* progression-free survival, *CR PFS* clinical/radiological PFS, *M* male, *F* female, *PR* partial response, *SD* stable disease, *PD* progressive disease, *BPI* Brief Pain Inventory.

### Translational research

Differential gene expression analysis was carried out by grouping samples according to RECIST response [responders (*n* = 5) vs. non-responders (*n* = 11)]. A total of 865 genes were significantly (*p* < 0.05) and differently expressed according to RECIST response; 383 and 482 were overexpressed or underexpressed, respectively, in the samples of patients that had a PR to nab-paclitaxel, compared to non-responders.

Among these genes, 43 genes were significantly and differently overexpressed in the responders group, when considering the genes with a logFC >1 (Supplementary Table 3). No gene was significantly and differently underexpressed in the responders group when a logFC < −1 cut-off was applied.

EnrichR found two MSigDB Hallmarks that significantly overlapped with genes differently expressed between patients that responded to the treatment or experienced disease progression. Those two pathways are PI3K/AKT/mTOR signaling and Angiogenesis (Supplementary Fig. 1). Angiogenesis enrichment was supported by *THBD*, *VTN*, SPP*1*, *LPL* and *FGFR1*. PI3K/AKT/mTOR signaling was supported by *TIAM1*, *PIKFYVE*, *GRK2*, *PRKCB*, *CAMK4*, *MKNK2*, *ITPR2*, *MAPK1*, *TRIB3*, and *FGF22*.

**Fig. 3:**
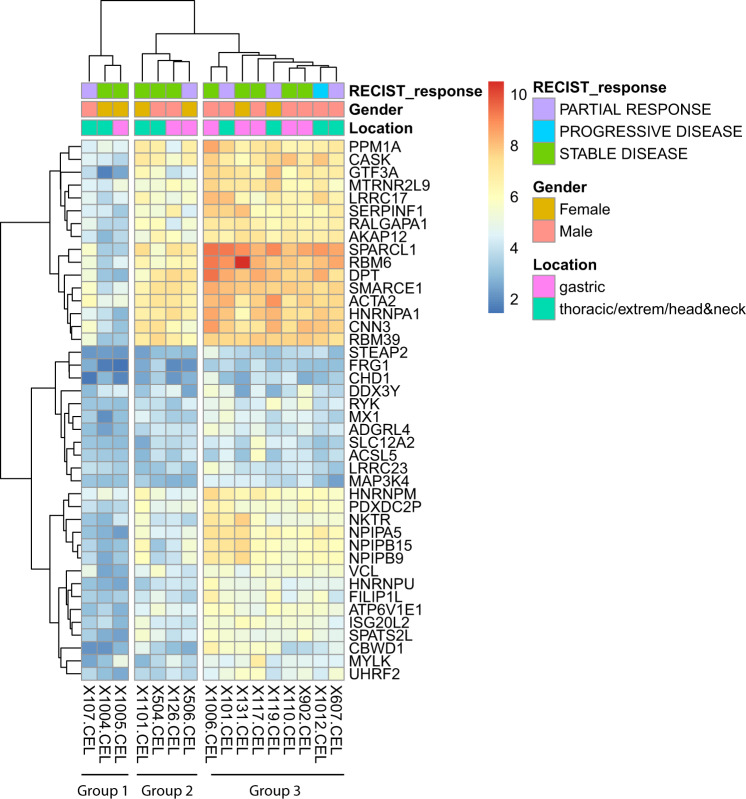
Heatmap clustering of patients based on gene expression profiling. Patients (columns; *n* = 16) were clustered in three groups based on a combination of mRNA differentially expression data (rows), modeled according to patient response (fitting a linear model through limma package). Zooming to column-wise cluster annotation, samples are clustered, with k-mean clustering method, according to three features: gender (m/f), location of the tumor and type of response [RECIST classification: Partial Response (PR), Stable Disease (SD) or Progression Disease (PD)]. Source data are provided as a Source Data file.

The unsupervised clustering carried-out using the 43 gene-set grouped patients into 3 different subsets (Fig. 3). Interestingly, a significant correlation was found favoring group 3 with respect to RECIST response achievement, 56% vs 0% (*p* = 0.034) for joined groups 1 and 2.

## Discussion

In this phase II trial, we found an early clinical and radiological, centrally reviewed response, of 89 and 20%, respectively. This outcome was achieved in a series of 35 progressing patients by radiological RECIST 1.1, clinical criteria or both, and evaluable for the two main endpoints. Hence, this short-term regimen with nab-paclitaxel was active and safe in DF, with the accomplishment of the alternative hypothesis of obtaining at least 40% response in either of the two main endpoints. The 2-point decrease in the BPI-SF score of worst pain was conservatively selected following previous recommendations^23,24^ and was probed to be clinically meaningful in a previous phase II trial exploring denosumab in aggressive giant cell tumor of bone patients, a similar context of a locally aggressive mesenchymal tumor with null or very low metastatic potential^25^.

Composite main endpoints are rarely seen in clinical trials, but in recent times are increasingly being considered^26^. In the case of DF, this is of paramount importance since a single primary endpoint does not provide a comprehensive clear image of what active treatments are potentially attaining. In many circumstances, clinical impairment in the absence of radiological RECIST progression of DF represents a worthy argument for starting therapy^11^. By contrast, patients may dramatically improve their symptoms even when no RECIST response was obtained^16^. This has also been the case of our patients where the proportion of subjects with clinical response was 89%, a fourfold increase in comparison with radiological responses. Additionally, the time to reach a response may also importantly be perceived by patients. Few studies address this question. One prospective study acknowledged that time to clinical response was a matter of a few weeks after systemic treatment with gamma-secretase inhibitor, but it was not quantified^16^. In a retrospective study, the median time to clinical response with toremifene, defined as any pain improvement, was 4 months and the authors communicated a total of 75% of pain improvement in 16 symptomatic patients^11^. Other retrospective series treated with hormone therapy, in this case tamoxifen, reported 31% of symptomatic relief from a series of 35 DF patients^27^. Additionally, the time to radiological response reported in a series with systemic treatments was around 10 months^15–17^. Our prospective trial compares favorably with these previous studies, showing a median time to clinical and radiological responses as short as 0.9 and 6.3 months, respectively. Notably, the time to spontaneous regression or stabilization is barely mentioned in published series, but it takes more than one year^17–19^.

Other outcomes such as PFS and duration of response are also crucial in DF, indicating how long certain therapeutic strategies are able to concede disease control for both symptomatic and radiologically aspects. Reports of PFS in some larger series of DF with active surveillance were 2-year PFS of 71%^28^, 3-year PFS of 38%^29^, or 5-year PFS rate of 49.9%^7^. Regarding PFS reports in series treated with systemic therapies, the 2-year PFS rates usually exceed 80%^11,17^, and 5-year PFS rates ranged from 55 to 81%^15,30^. These previous reports have focused on PFS following RECIST; in this respect our study reports a competitive 2-year PFS rate of 86% by central radiology review. Importantly, a recent phase 3 trial in DF communicated 2-year PFS rates of 81 and 36% for sorafenib and placebo arms, respectively. This side of the coin should also be taken into account in some contexts where active surveillance may not be the best choice. In line with this, our series might represent an extreme in the spectrum of biological behavior of DF: all patients were symptomatic, and with radiological or clinical progression, or both. Moreover, the proportion of abdominal wall DF, the most favorable site for regression or PFS^8^, constituted only a 7% in our study. In the case of regorafenib trial, the patients were enrolled in progression, defined as size increase of ≥10%, or symptomatic or with unresectable tumors. Further, the trial enrolled a substantial proportion of abdominal cases 28 and 43% for regorafenib and placebo arms, respectively^17^. On the other hand, the pazopanib trial enrolled progressive, but not all symptomatic, patients^31^. In addition, neck DT accounted for 2% compared to 15% in our study.

In contrast to the long-lasting schemes of systemic treatment that typically have been used in DF, our proposal intended to offer a short-term regimen, whilst the other benefits, such as activity and safety, are maintained. The classic weekly low-dose of methotrexate plus vinca alkaloids duration is around 1 year^14,32^, and it is not free of concerning toxicity as neutropenia grade 3 or peripheral neuropathy^31^ that impelled investigators to make modifications such as a biweekly administration, but still with a median duration that exceeded one year^15^. The duration of targeted therapies in DF trials has been similar or even longer. Thus, imatinib duration in DF ranged between 1 and 2 years^12,33^, sorafenib was administered without specific duration and widely exceeded one year^17^, pazopanib was prescribed for one year^31^, and the median duration with gamma secretase inhibitors was more than two years^34^. Despite these studies communicating manageable treatment-emergent adverse effects, they also reported grade 3/4 toxicities in the range of 11 to 45% for Imatinib, 56% for pazopanib, 31% for sorafenib, and 47% for gamma secretase inhibitors. Accordingly, shorter schemes, such as our tested regimen, represent a rational alternative attaining earlier and durable responses while avoiding sustained toxicity related with more durable treatments. Nevertheless, our short scheme was not enough to maintain clinical and/or radiological benefits in DF of the neck or proximal upper extremities. In line with this, DF of the neck have been related to a higher recurrence rate in previous publications^35,36^, and DF of the limbs resulted in a more unfavorable location than the trunk in some reports^8^.

A limitation of this trial is that, even when all cases were centrally reviewed before accrual and exhibited a characteristic immunostaining profile, the mutational status was available for only 55% of cases. The difficulties in selecting an adequate questionnaire for measuring functionality in all the DF locations hindered the systematic collection of some dramatic improvements in this area.

The finding of enrichment for angiogenesis and mTOR-related genes among those differently expressed in RECIST responders or non-responders, is in line with our expectations. Nab-paclitaxel was supposed to act as an antiangiogenic through the inhibition of some cytokines such as bFGF and VEGF, and by other interfering mechanisms. Interestingly, the inhibition mTOR pathway can reduce the process of angiogenesis through the inhibition of HIF-1α^37^. The statistically significant correlation favoring group 3 with higher probability to obtain a RECIST reponse, gives us new insights on potential new targets and predictive gene- signature that should be analyze on the whole series and then externally validated with other series using antiangiogenic in DF.

Since DF behavior is heterogeneous, wait-and-see options should not be universally recommended. Developing new strategies, such as shorter regimens, can be beneficial for patients if toxicity can be minimized whilst maintaining adequate activity.

Three-month nab-paclitaxel administration showed to be active, quick to achieve responses, durable for maintaining responses (except for neck and proximal upper extremities) and safe. This regimen warrants further investigation in symptomatic and progressing DF.

## Methods

### Study design

ABRADES (Abraxane in desmoid and desmoplastic tumors) study, a phase II, non-randomized, open-label, multicenter clinical trial with two different cohorts, was sponsored by the Spanish Group for Research on Sarcoma (GEIS) and conducted in 14 Spanish sarcoma expert centers, in pediatric and adult populations (aged ≥6 months). (ClinicalTrials.gov identifier NCT03275818). Data from the DF cohort is presented here, from which 8 centers were actively accruing patients. The study protocol was approved by a central ethics committee (“Provincial de Sevilla” Research Ethics Committee (Seville, Spain)), covering all participating centers at national level in Spain. Procedures were performed in accordance with good clinical practice (GCP) guidelines and in compliance with the Declaration of Helsinki. All patients signed a written informed consent form to participate in the study, including consent for use of tumoral samples. Patients aged ≥6 months and ≤20 years received nab-paclitaxel 240 mg/m^2^, while patients aged ≥21 years received nab-paclitaxel 125 mg/m^2^, on days 1, 8, and 15 in cycles of 28 days. Patients in the DF cohort received a maximum of three cycles. Nab-paclitaxel was administered intravenously over 30 min, without corticosteroid or antihistamine premedication. Central pathology review was a compulsory requirement for trial entry. Radiological assessment was only allowed by MRI, with perfusion and diffusion sequences being mandatory, specific thorough determinations are reflected in the protocol. Studies with MRI were performed at baseline and every 3 months for the first year, and then every 6 months for two additional years. Central radiological anonymous assessment was compulsory for all imaging studies. Brief Pain Inventory - Short Form (BPI-SF) was collected at baseline, days 1, 8, and 15, at the end of treatment and every 3 months for the first 3 years. Additionally, analgesic quantification algorithm (AQA) was collected at the same intervals as the BPI-SF. More details can be found within the protocol through this link: https://grupogeis.org/GEIS-39_Abrades_Protocol_V2_220218-clean.pdf. A copy of this protocol is also available as a Supplementary Note in the Supplementary Information File.

### Patients

Eligible patients had centrally confirmed histology of DF; measurable disease according to RECIST 1.1 criteria; and which score was at least 2 points in the worst pain section of the BPI-SF. It was determined that this decrease in the score was the minimally important difference (MID) that patients perceived as relevant and that would have led the clinician to plan a change in the patient’s management^23^. The MID for BPI-SF was conservatively defined as 2 points in the score on the basis of anchor-based methods^24^ and previous similar experience^25^. Additional criteria were Eastern Cooperative Oncologic Group (ECOG) performance status ≤1, deep DF from extremities, trunk or head and neck regions, intra-abdominal DF were enrolled if harboring *CTNNB1* mutation. Patients had to have clinical or radiological progression in the previous 6 months, and adequate bone marrow, renal and liver function. Normal cardiac function with left ventricular ejection fraction of at least 50% had to be demonstrated by echocardiogram or by multigated acquisition scan. Previous chemotherapy, prostaglandin inhibitors or hormone therapy were allowed. Availability of a paraffin embedded tumor block for central pathology confirmation and translational research was mandatory. An effective method of contraception had to be used in men or women with childbearing potential, before entry into the study, throughout the treatment and for 6 months after ending the study treatment. Most relevant exclusion criteria were prior taxanes therapy, more than one previous chemotherapy line, and unavailability to undergo MRI, DF tumors with ill-defined margins, previous irradiated target lesion or women who are pregnant or breast-feeding. Secondary side effects were collected according to CTCAE 4.0. The first patient was enrolled in 9 May 2017 and the last patient was enrolled in 27 September 2019.

### Statistics and reproducibility

The primary endpoint of the study was double: overall response rate (ORR) according to RECIST 1.1 or clinical response with an improvement of at least 2 points in the BPI-SF worst pain score, in the absence of PD. Secondary objectives were progression-free survival (PFS) measured as a median, variation of symptoms other than pain and also scored in the BPI-SF and analgesic consumption during the first year, analysis of the safety profile of nab-paclitaxel and central assessment of the pattern of responses in different sequences of MRI. For sample size estimation, a feasible Simon two-stage design was used, selecting error rates alpha equal to 5% and beta equal to 20%. The option related to efficacy was 40% of either ORR and/or clinical response, whatever of both. Success, defined as ORR and/or clinical response in 20% of the cases or less will be considered as unacceptable and would not warrant further investigation (null hypothesis, H0 20%). While success defined as ORR and/or clinical response in 40% of the cases or more will be considered as an acceptable result warranting further investigation of the drug in DF (alternative hypothesis, H1 40%). A total of 21 eligible and treated patients was estimated to be included in the first stage. If ≤4 patients had radiological and/or clinical response, the trial would be stopped. Otherwise, with >4 patients with radiological and/or clinical response, the trial will continue to accrue up to 35 eligible and evaluable patients to enter the study. If 12 or more successes were observed in those 35 subjects, it will be concluded that the results of the trial warrant further investigation^38^. Comparisons between qualitative variables were done using the Fisher Exact Test or Chi-square. Time to event variables (OS and PFS) were measured from the date of therapy onset and were estimated according to the Kaplan-Meier method. Comparisons between the variables of interest were performed by the log-rank test. All p-values reported were 2-sided, and statistical significance was defined at *p* < 0.05. Statistical analysis was performed with SSPS version 28.

### Translational research

Gene expression profiling was performed using Clariom™ S Pico human assay (Applied Biosystems™; ThermoFisher Scientific, Inc.; Foster City, CA, USA), which accurately measured the expression levels of more than 20,000 well-annotated genes, in order to obtain a transcriptome-wide gene-level expression profile. Sixteen samples were used in this exploratory analysis due to budget restrictions. Briefly, RNA was amplified and labeled using the GeneChip® WT PLUS Reagent Kit (Thermo Fisher Scientific, Inc.; Waltham, MA, USA). Amplification was performed with 100 ng of total RNA input, following the procedures described in the WT PLUS Reagent Kit user manual. The amplified cDNA was quantified, fragmented, and labeled to hybridize to GeneChip® Clariom S Human Array (Thermo Fisher Scientific, Inc.), using 5.5 μg of single-stranded cDNA product and following manufacturers’ protocols. Washing, staining (GeneChip® Fluidics Station 450, Thermo Fisher Scientific, Inc.), and scanning (GeneChip® Scanner 3000, Thermo Fisher Scientific, Inc.) were performed following the protocols outlined in the user manual for cartridge arrays.

### Bioinformatics analysis

Arrays were processed (background corrected, quantile normalized and summarized) via the RMA (Robust Multi-array Average) method from the oligo package (v1.54.1). Summarization was achieved with the corresponding custom CDF (pd.clariomshuman.hs.entrezg v25.0.0) from Brainarray (http://brainarray.mbni.med.umich.edu/Brainarray).

Differential expression analyses were performed using the Ebayes function in the limma package (v3.46.2). Analyses was executed taking into account RECIST 1.1 response [responders (*n* = 5) vs. non-responders (SD and PD; *n* = 11)], using non-responders as the reference for data analyses. Pathway enrichment was accomplished with Enrichr^39^ on the Hallmark gene sets (MSigDB Hallmark 2020)^40^, restricting the input to those genes showing differential expression (*p*-value ≤ 0.05). Benjamini and Hochberg correction for multiple comparisons was applied and a *p*-value threshold of 0.05 was defined^41^. A clustering was performed in the final filtered gene-set using FactoMineR and Factoextra R packages in an unsupervised manner relying on both Gap Statistic and Average Silhouette methods to determine the optimal number of groups for the K-means algorithm. Costumed Heat map was produced using pheatmap R package.

### Reporting summary

Further information on research design is available in the Nature Research Reporting Summary linked to this article.

## Supplementary information

Supplementary Information

Reporting Summary

Supplementary Data 1

Supplementary Data 2

## Data Availability

The Microarray dataset is available in the ArrayExpress database under accession code E-MTAB-12163. The clinical variables used for the translational research and raw gene expression data are available in the ArrayExpress database under the access [E-MTAB-12163]. Complete clinical data is provided in Supplementary Data 1 and 2. Data from patients was anonymized. All the identifiers, with the exception of gender, have been omitted. Shared public results implied statistical results (significant tests), displayed in form of tables (i.e. data that passes a filter) and statistical plots (e.g. differentially expressed genes heatmaps). Shared tables contemplate normalized genes counts, filtering for genes significance and genes differential expression. Plots include expression heatmaps. The pipeline of the study is shared in detail in the methods section of the paper. The study protocol is available in the Supplementary Information file. The remaining data are available within the Article, Supplementary Information, Supplementary Data or Source Data file.

## Code availability

The custom code is permanently available in the ArrayExpress database under accession code E-MTAB-12163.
