# Supplementary material for: A phase II trial of weekly nab-paclitaxel for progressive and symptomatic desmoid tumors
Source: Nat Commun. 2022 Oct 21;13:6278. doi: 10.1038/s41467-022-33975-6 (PMC9587294; doi:10.1038/s41467-022-33975-6)
Supplement: Supplementary file 1 — Supplementary Information [file 41467_2022_33975_MOESM1_ESM.pdf]

Supplementary Table 1 – Trial efficacy data

	GLOBAL SERIES (N=40)	EFFICACY (clinical) (N=36)	EFFICACY (RECIST) (N=39)	EFFICACY (clinical & RECIST) (N=35)
Local response:				
- PR	10 (25%)	9 (25%)	10 (26%)	9 (26%)
- SD	30 (75%)	27 (75%)	29 (74%)	26 (74%)
Central response:				
- PR	9 (22%)	7 (19%)	9 (23%)	7 (20%)
- SD	29 (72%)	27 (75%)	29 (74%)	27 (77%)
- PD	1 (2%)	1 (3%)	1 (3%)	1 (3%)
- Not evaluable	1 (2%)	1 (3%)		
Median dimension change RECIST (%)	-8.7 (-77.6%-40)	-8.7 (-77.6%-40)	-8.7 (-77.6%-40)	-8.7 (-77.6%-40)
Change in T2* (%):				
- No change	3 (7%)	3 (8%)	3 (8%)	3 (9%)
- 0-4	10 (25%)	10 (28%)	10 (26%)	10 (29%)
- 5-9	3 (7%)	3 (8%)	3 (8%)	3 (9%)
- 10-24	6 (15%)	6 (17%)	6 (15%)	6 (17%)
- >24	12 (30%)	9 (25%)	12 (30%)	9 (26%)
- Not evaluable	6 (15%)	5 (14%)	5 (13%)	4 (11%)
Change in painkillers use (AQA):				
- -3	3 (7%)	2 (6%)	3 (8%)	2 (6%)
- -2	2 (5%)	2 (6%)	2 (5%)	2 (6%)
- -1	8 (20%)	7 (19%)	8 (20%)	7 (20%)
- 0	23 (57%)	21 (58%)	23 (59%)	21 (60%)
- 1	2 (5%)	2 (6%)	2 (5%)	2 (5%)
- No data	2 (5%)	2 (6%)	1 (3%)	1 (3%)
Reduction in pain (BPI Worst pain)				
- 0 a -1	6 (19%)	4 (11%)	8 (20%)	4 (12%)
- At least -2	25 (81%)	32 (89%)	31 (79%)	31 (89%)
- At least -4	17 (55%)	21 (58%)	20 (51%)	20 (57%)
- At least -6	12 (39%)	11 (30%)	11 (28%)	11 (31%)
Median time to local BR, months (range)	2.9 (0.5-12.2)	2.9 (0.5-12.2)	2.9 (0.5-12.2)	3 (0.5-12.2)
Median time to central BR, months (range)	6.3 (2.3-18.6)	6.3 (2.3-18.6)	6.3 (2.3-18.6)	6.3 (2.3-18.6)
Median time to BPI reduction, months (range)	1 (0.2-2.9)	1 (0.2-2.9)	0.9 (0.2-2.9)	0.9 (0.2-2.9)
Median duration of local response, months (range)				
- PR	27 (17-42)	-	27 (17-42)	28 (17-42)
- SD	21 (2-44)	-	20 (2-44)	19 (2-44)
Median duration of central response, months (range)				
- PR	32 (24-42)	-	32 (24-42)	32 (24-42)
- SD	27 (11-44)	-	27 (11-44)	27 (11-44)
- PD	2 (2-2)	-	2 (2-2)	2 (2-2)

PR: partial response; SD: stable disease; PD: progressive disease; AQA: Analgesic Quantification Algorithm; BPI: Brief pain inventory.

* Change in T2: defined as the percentage in changes of the fibrous component of the tumor in T2-enhanced sequence during therapy

Supplementary Table 2 – Univariate analysis of the influence of clinical factors in radiological and clinical endpoints (n=40)

	% RECIST Response	P	% BPI-SF* at least -2 points	P	% BPI-SF* at least -6 points	p
Median age (years):		0.69		0.69		1
- 18-38	16%		75%		15%	
- >38	25%		85%		10%	
Gender (M/F):		0.016		0.1		0.32
- Male	43%		64%		21%	
- Female	8%		88%		8%	
Median time diagnosis to enrollment (range):		1		0.24		0.018
- 0-17	19%		71%		0%	
- >17	22%		89%		26%	
Previous relapses:		0.69		0.69		0.071
- No	25%		75%		0%	
- Yes	17%		83%		21%	
Previous surgery:		0.45		0.44		0.049
- No	26%		74%		0%	
- Yes	15%		86%		24%	
Previous chemotherapy:		1		1		0.15
- No	21%		79%		9%	
- Yes	20%		83%		33%	
Location:		0.4		0.4		0.6
- Head and neck & Proximal upper extremity	25%		76%		10%	
- Other	9%		91%		18%	
ECOG BASELINE:		0.037		0.69		0.14
- 0	40%		73%		0%	
- 1	8%		83%		21%	
Change in T2 enhance sequence (MRI)#:		1		1		1
- 0-7.5	25%		81%		6%	
- >7.5	22%		78%		11%	
New score:		0.43		0.68		0.72
- No change	0%		100%		0%	
- 0-4	30%		70%		10%	
- 5-9	33%		100%		0%	
- 10-24	0%		83%		0%	
- >24	33%		75%		17%	

*BPI-SF Worst pain: #Change in T2: defined as the percentage in changes of the fibrous component in the tumor in T2-enhanced sequence during therapy. Two-sided Fisher's exact test. Multiple comparisons were not used for this analysis.

Supplementary Table 3 – Genes significantly and differently expressed according to response, with a logFC < -1 or >1

Gene	logFC*	p.value	adjusted p.value
DPT	1.919	0.021	0.819
SPARCL1	1.837	0.035	0.819
RBM6	1.761	0.036	0.819
GTF3A	1.692	0.048	0.819
HNRNPA1	1.519	0.037	0.819
NPIPA5	1.518	0.039	0.819
CNN3	1.439	0.041	0.819
NPIPB15	1.404	0.042	0.819
RBM39	1.399	0.044	0.819
NKTR	1.374	0.027	0.819
NPIPB9	1.328	0.043	0.819
CBWD1	1.327	0.038	0.819
DDX3Y	1.324	0.002	0.819
SMARCE1	1.313	0.020	0.819
AKAP12	1.300	0.033	0.819
SERPINF1	1.228	0.050	0.819
CASK	1.214	0.044	0.819
RALGAPA1	1.206	0.021	0.819
MX1	1.201	0.001	0.819
LRRC17	1.185	0.040	0.819
PPM1A	1.150	0.037	0.819
MYLK	1.140	0.013	0.819
ADGRL4	1.119	0.001	0.819
HNRNPU	1.110	0.018	0.819
CHD1	1.107	0.008	0.819
HNRNPM	1.088	0.008	0.819
STEAP2	1.081	0.002	0.819
SPATS2L	1.073	0.023	0.819
UHRF2	1.068	0.014	0.819
RYK	1.045	0.005	0.819
MTRNR2L9	1.044	0.033	0.819
LRRC23	1.043	0.000	0.819
VCL	1.040	0.024	0.819
SLC12A2	1.031	0.002	0.819
MAP3K4	1.030	0.001	0.819
PDXDC2P	1.029	0.015	0.819
ISG20L2	1.026	0.012	0.819
ACTA2	1.024	0.037	0.819
FRG1	1.020	0.003	0.819
ACSL5	1.007	0.003	0.819
ATP6V1E1	1.003	0.030	0.819
ANKRD36B	1.002	0.050	0.819
FILIP1L	1.000	0.037	0.819

*positive logFC means that gene expression levels are higher in desmoid tumors that had a response to NAB-paclitaxel. Two-sided Limma package (v3.46.2) from R Bioconductor was implemented with default parameters. Benjamini & Hochberg correction for multiple comparisons was applied.

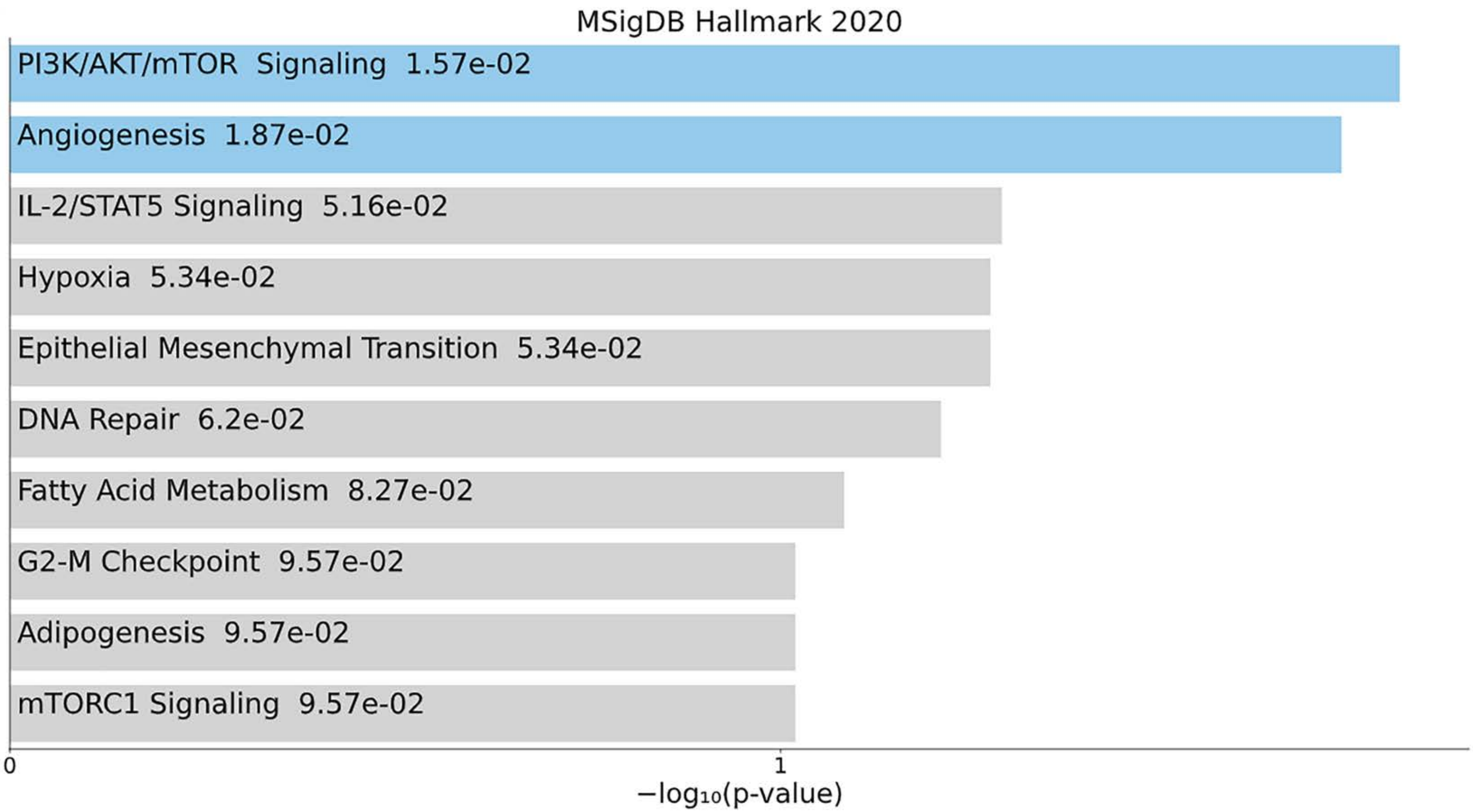

Supplementary Figure 1- EnrichR pathway analysis. Blue bars correspond to significant pathways. PI3K/AKT/mTOR Signaling (p-value: 0.015734, members: *TIAM1*, *PIKFYVE*, *GRK2*, *PRKCB*, *CAMK4*, *MKNK2*, *ITPR2*, *MAPK1*, *TRIB3*, and *FGF22*. Angiogenesis (p-value: 0.018715, members: *THBD*, *VTN*, *SPP1*, *LPL*, and *FGFR1*). The p values, obtained in the enrichment analysis with both upregulated and downregulated genes, were adjusted with Benjamini and Hochberg method.

GEIS-39

CLINICAL TRIAL PROTOCOL

Title:

Phase II trial of nab-paclitaxel for the treatment of desmoid tumors and multiply relapsed/refractory desmoplastic small round cell tumors and Ewing's sarcoma

Sponsor Protocol Number: GEIS-39
EudraCT Number: 2016-002464-14
Study Acronym: ABRADES

Protocol Version: 2 of 22/02/2018

Sponsor: Grupo Español de Investigación en Sarcomas (GEIS)

Coordinating Clinical Investigators:

- Dr. Javier Martín – Hospital Universitario Virgen del Rocío (Seville)
- Dr. Jaume Mora – Hospital Sant Joan de Déu (Barcelona)

Translational Research Coordinators:

- Dr. David da Silva Moura – Hospital Universitario Virgen del Rocío (Seville)
- Dr. Ángel Montero – Hospital Sant Joan de Déu (Barcelona)

The information contained in this document is confidential and cannot be revealed to other persons without the written authorization of the investigators, except its use to obtain the informed consent of the subjects who shall receive the drug under investigation, in addition to communications to health authorities, clinical trial ethics committees or those professionals appointed to carry out the study.

PROTOCOL VERSION CHRONOLOGY

Version	Date	Amendment No.	Summary of Changes
1	01/12/2016	NA	• Original protocol
2	22/02/2018	3*	• S3: Primary objectives for DT modified. Physical function variation added as secondary objective for DT.<br>• S4: Inclusion criteria for DT (4.1.1) changed (1, 2. 3, 4, 5, 10, previous criterion 9 removed). Exclusion criteria for DT (4.1.2) changed (1, 3, 7, 13). Inclusion criteria for DSRCT/ES (4.2.1) changed (1, 5, 11). Exclusion criteria for DSRCT/ES (4.2.2) changed (7).<br>• S5.1: Upper age limit removed. New dose for patients weighing 10 kg or less.<br>• S5.3: At end of section it is mentioned that treatment must be stopped if additional reductions required.<br>• S6.1: Drug distribution channel changed.<br>• S7.1: Calendar of assessments divided into two (one for each cohort). Changes for DT calendar: 3 cycles maximum, serologies at baseline, AEs collected from consent signature, MRI must include diffusion and perfusion, Legends 2, 4 and 8 modified. Changes for DSRCT/ES calendar: Cycles up to progression, serologies at baseline, AEs collected from consent signature,<br>• S7.3: CRO contact changed.<br>• S7.5: Table 5 parameters changed.<br>• S7.7: MRI type for DT and frequency of radiological tests specified. CRO contact changed.<br>• S7.11: Follow-up visits text changed.<br>• Section 8.5: "parent/guardian" is specified. SAEs collected up to 28 days after last dose.<br>• S10.2: DT endpoints expressed differently. Physical function variation added as secondary endpoint for DT.<br>• S10.3: Statistical plan for DT updated.<br>• S11.3: CRO contact changed.

***Note:** Amendments 1 and 2 did not include changes to the protocol; they were just related to the addition of sites in Spain.

CONTACT INFORMATION

Sponsor:

Grupo Español de Investigación en Sarcomas (GEIS)
C/ Diego de León, 47
28006 Madrid, Spain
www.grupogeis.org

CRO appointed by the Sponsor as main contact point:

Sofpromed Investigación Clínica, SLU
C/ del Ter, 27 2nd Floor – Office 3
07009 Palma de Mallorca, Spain
Tel: +34 648 414 261
Fax: +34 971 570 222
E-mail: ensayos@sofpromed.com

LIST OF TABLES

Table 1: Dose modifications for hematological toxicities ... 30

Table 2: Dose modifications for other toxicities ... 31

Table 3: Procedures and assessments (Desmoid tumor) ... 35

Table 4: Procedures and assessments (Desmoplastic small round cell tumor and Ewing's sarcoma) ... 36

Table 5: Clinical laboratory assessments ... 39

Table 6: Choi/RECIST criteria for response evaluation ... 41

PROTOCOL SIGNATURES PAGE

Sponsor: Grupo Español de Investigación en Sarcomas (GEIS)

Study Title: Phase II trial of nab-paclitaxel for the treatment of desmoid tumors and multiply relapsed/refractory desmoplastic small round cell tumors and Ewing's sarcoma

Sponsor Protocol Number: GEIS-39

EudraCT Number: 2016-002464-14

Study Acronym: ABRADES

Protocol Version and Date: Version 2 of 22/02/2018

I read this protocol and I accept to conduct this trial in accordance with the protocol stipulations, GCP guidelines and the Declaration of Helsinki.

Coordinating Investigators:

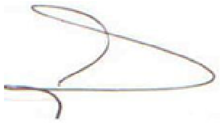

……………………………..
Dr. Javier Martín
Hospital Universitario Virgen del Rocío (Seville)
Desmoid tumor cohort

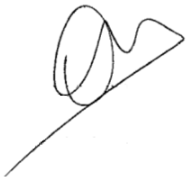

……………………………..
Dr. Jaume Mora
Hospital Sant Joan de Déu (Barcelona)
DSRCT and Ewing's sarcoma cohort

Local Site Principal Investigator Name:……………………………………………..

Site Name:……………………………………………………………………….……..

Local Site Principal Investigator Signature:…………………………………………

Signature Date:…………………………………………………………………………

TABLE OF CONTENTS

LIST OF TABLES **4**

GLOSSARY OF ABBREVIATIONS **8**

1. TRIAL SUMMARY **9**

2. INTRODUCTION **18**

2.1 Background and scientific rationale 18

2.2 Nab-paclitaxel 23

3. CLINICAL STUDY OBJECTIVES **25**

4. SELECTION OF SUBJECTS **26**

4.1 Inclusion and exclusion criteria for cohort 1: DT 26

4.2 Inclusion and exclusion criteria for cohort 2: DSRCT and ES 27

4.3 Treatment discontinuation 28

4.4 Subject discontinuation from the study 28

4.5 Screening failures 28

5. TREATMENT **30**

5.1 Treatment administration 30

5.2 Body surface area or weight changes 30

5.3 Dose modifications 30

5.4 Treatment delays 31

5.5 Missed treatment 32

5.6 Treatment overdose 32

5.7 Concomitant medications and procedures 32

6. DRUG MANAGEMENT **34**

6.1 Supply and distribution of study drug 34

6.2 Packaging and labeling 34

6.3 Accountability and disposal 34

6.4 Drug compliance 34

6.5 Local pharmacy responsibilities 34

7. STUDY PROCEDURES AND ASSESSMENTS **35**

7.1 Calendar of activities 35

7.2 Subject information and consent 37

7.3 Eligibility and enrollment procedures 37

7.4 Clinical assessments 38

7.5 Laboratory assessments 38

7.6 Safety assessments 40

7.7 Efficacy assessments 41

7.8 Pain assessments 43

7.9 Treatment management 43

7.10 Biological sample collection 43

7.11 Survival 43

8. PHARMACOVIGILANCE **44**

8.1 Monitoring, Recording and reporting of adverse events 44

8.2 Evaluation of adverse events 44

8.3 Abnormal laboratory values 46

8.4 Pregnancy ... 47
8.5 Reporting of serious adverse events ... 47
8.6 Expedited reporting of adverse events ... 48
9. DATA MANAGEMENT ... 49
9.1 Electronic case report form (e-CRF) ... 49
9.2 Data reviews ... 49
9.3 Record keeping ... 50
10. STUDY DESIGN AND STATISTICS ... 51
10.1 Study design ... 51
10.2 Study endpoints ... 51
10.3 Sample size ... 51
10.4 Efficacy analysis ... 52
10.5 Safety analysis ... 52
11. TRANSLATIONAL STUDY ... 53
11.1 Translational objectives ... 53
11.2 Methods and experiments ... 53
11.3 Biological sample collection ... 55
12. ETHICAL AND REGULATORY ASPECTS ... 56
12.1 Ethics committees ... 56
12.2 Competent authorities ... 56
12.3 Ethical conduct of the study ... 56
12.4 Subject privacy ... 56
12.5 Subject information sheet and consent ... 57
12.6 Insurance policy ... 57
13. TRIAL GOVERNANCE AND RESPONSIBILITIES ... 58
13.1 Trial Steering Committee (TSC) ... 58
13.2 Trial Management Group (TMG) ... 58
13.3 Sponsor responsibilities ... 58
13.4 Principal investigators responsibilities ... 58
14. STUDY DEVELOPMENT CONSIDERATIONS ... 59
14.1 Inclusion of the study in clinical trial registries ... 59
14.2 Quality control and quality assurance ... 59
14.3 Definition of end of study ... 59
14.4 Sponsor discontinuation criteria ... 59
14.5 Publication of results ... 59
APPENDIX A: PAIN AND SYMPTOMS ASSESSMENT ... 61
APPENDIX B: DETERMINATION OF CREATININE CLEARANCE (Cl_{CR}) ... 62
APPENDIX C: DECLARATION OF HELSINKI, WORLD MEDICAL ASSOCIATION GENERAL ASSEMBLY ... 63
REFERENCES ... 67

GLOSSARY OF ABBREVIATIONS

AE	Adverse Event
ALT	Alanine Aminotransferase
AST	Aspartate Aminotransferase
BP	Blood Pressure
BSA	Body Surface Area
CBR	Clinical Benefit Rate
CR	Complete Response
e-CRF	Electronic Case Report Form
CRO	Contract Research Organization
CT	Computed Tomography
DVT	Deep Venous Thrombosis
DP	Disease Progression
DBP	Diastolic Blood Pressure
ECG	Electrocardiogram
ECOG	Eastern Cooperative Oncology Group
EudraCT	European Union Drug Regulating Authorities Clinical Trials
GCP	Good Clinical Practice
GEIS	Grupo Español de Investigación en Sarcomas
HPF	High Power Field
ICH	International Conference on Harmonization
IMP	Investigational Medicinal Product
INR	International Normalized Ratio
ISF	Investigator Site File
L	Liter
LVEF	Left Ventricular Ejection Fraction
mg	Milligrams
mg/m^2	Milligrams per meter squared
mg/kg	Milligrams per kilogram
MRI	Magnetic Resonance Imaging
µg	Microgram
mL	Milliliter
mL/min	Milliliter per minute
mm	Millimeter
ms	Millisecond
MUGA	Multi-gated Radionuclide Angiography
MVD	Microvessel Density
NCI CTCAE 4.0	National Cancer Institute Common Terminology for Coding of Adverse Events version 4.0
ORR	Objective Response Rate
OS	Overall Survival
PD	Progressive Disease
PET	Positron Emission Tomography
PFS	Progression-Free Survival
PK	Pharmacokinetics
PR	Partial Response
RECIST	Response Evaluation Criteria in Solid Tumors
SAE	Serious Adverse Event
SD	Stable Disease
SGOT	Serum Glutamic Oxaloacetic Transaminase
SGPT	Serum Glutamic Pyruvic Transaminase
SmPC	Summary of Product Characteristics
SBP	Systolic Blood Pressure
STS	Soft Tissue Sarcoma
SUSAR	Suspected Unexpected Serious Adverse Reaction
TKI	Tyrosine Kinase Inhibitor
TIA	Transient Ischemic Attack
TMG	Trial Management Group
TSC	Trial Steering Committee
ULN	Upper Limit of Normal

1. TRIAL SUMMARY

**Trial Title**	Phase II trial of nab-paclitaxel for the treatment of desmoid tumors and multiply relapsed/refractory desmoplastic small round cell tumors and Ewing's sarcoma
**Sponsor Protocol Number**	GEIS-39
**EudraCT Number**	2016-002464-14
**Study Acronym**	ABRADES
**Study Type**	A two-cohort, phase II, open-label, non-randomized, multicenter clinical trial of a licensed IMP
**Sponsor**	Grupo Español de Investigación en Sarcomas (GEIS)<br>C/ Diego de León, 47<br>28006, Madrid, Spain<br>www.grupogeis.org
**CRO**	Sofpromed Investigación Clínica, SLU<br>C/ del Ter, 27 - 2nd Floor – Office 3<br>07009 Palma de Mallorca, Spain<br>Telephone: +34 648 414 261<br>Fax: +34 971 570 222<br>ensayos@sofpromed.com
**Coordinating Investigators**	Clinical study:<br>• Dr. Javier Martín - Hospital Universitario Virgen del Rocío (Seville)<br>• Dr. Jaume Mora - Hospital Sant Joan de Déu (Barcelona)<br>Translational study:<br>• Dr. David da Silva Moura - Hospital Universitario Virgen del Rocío (Seville)<br>• Dr. Ángel Montero - Hospital Sant Joan de Déu (Barcelona)<br>Central radiology review:<br>• Dr. José Manuel Morales - Hospital Universitario Virgen del Rocío (Seville)
**Planned Calendar**	• Administrative start-up: 3 months<br>• First subject first visit: 1st quarter 2017<br>• Total recruitment period duration: 30 months<br>• Follow-up period: 12 months<br>• Estimated end of study date: 3rd quarter 2020

<table>
<tr><td>Estimated Accrual Rate</td><td>3 cases per month</td></tr>
<tr><td>Clinical Study Objectives</td><td>Cohort 1: Desmoid tumor

Primary clinical study objectives

• To determine the overall response rate (ORR) according to RECIST 1.1 and/or clinical benefit rate (CBR) at 3 months with pain improvement of at least 2 points in the Brief Pain Inventory – Short Form (BPI-SF).

Secondary clinical study objectives

• To define the pattern of radiological response according to MRI parameters and to correlate it with CBR and Brief Pain Inventory (BPI) parameters.
• To estimate the efficacy of nab-paclitaxel as measured by the progression-free survival (PFS) assessed by median time.
• To analyze the variation of symptoms during the first year from trial enrollment in accordance with BPI and Analgesic Quantification Algorithm (AQA).
• To analyze the variation of physical function during the first year from trial enrollment.
• To evaluate the safety profile of nab-paclitaxel according to CTCAE 4.0.

Cohort 2: Desmoplastic small round cell tumor and Ewing's sarcoma

Primary clinical study objective

• To determine the objective response rate (ORR) in subjects with desmoplastic small round cell tumor and Ewing's sarcoma, using RECIST 1.1 criteria.

Secondary clinical study objectives

• To evaluate the safety profile of nab-paclitaxel according to CTCAE 4.0.</td></tr>
<tr><td>Translational Study Objectives</td><td>DT Cohort

1.- To analyze protein levels of potential predictive and/or prognostic biomarkers of the response to nab-paclitaxel. The biomarkers (β-catenin, APC, CD105, SPARC, MMP-7, FAS, FASL, THBS1, VEGF and VEGFR1-2) will be evaluated by tissue microarray (TMA)/ immunohistochemistry (IHC) from paraffin block tumor biopsies samples and/ or by ELISA from peripheral blood plasma samples.
2.- To analyze the role of nab-paclitaxel in modulating the expression of soluble factors (CD105, SPARC, FASL and VEGF). Soluble factors expression will be determined by ELISA and qRT-PCR from peripheral blood samples.
3.- To correlate protein expression of potential predictive and/ or prognostic biomarkers of the response to nab-paclitaxel (β-catenin, APC, CD105, SPARC, FAS, FASL, MMP-7, THBS1, VEGF and VEGFR1-2), with clinical endpoint (response rate, time to response, number of lines etc.) in DT.</td></tr>
</table>

	DSRCT and ES Cohort<br><br>1.- To correlate the activity of nab-paclitaxel against DSRCT and Ewing's sarcoma with the expression of SPARC by immunohistochemistry.<br>2.- To correlate the activity of nab-paclitaxel against DSRCT and Ewing's sarcoma with the loss of p16.<br>3.- To generate DSRCT and Ewing's sarcoma tumor xenografts from subjects being entered into the trial and evaluate the activity of nab-paclitaxel by preclinical pharmacology.
**Disease Under Study**	• Cohort 1: Subjects with desmoid tumor<br>• Cohort 2: Subjects with desmoplastic small round cell tumor or Ewing's sarcoma
**Sample Size**	60 subjects: Maximum of 35 subjects for cohort 1 (DT) and 25 for cohort 2 (DSRCT and ES)
**Treatment**	nab-paclitaxel (ABRAXANE) will be administered as follows:<br><br>• Age ≥ 21: 125 mg/m^2 days 1, 8 and 15 in cycles of 28 days<br>• Age ≥ 6 months and ≤ 20 years: 240 mg/m^2 (for patients weighing > 10 kg) and 11.5 mg/kg (for patients weighing ≤ 10 kg) on days 1, 8 and 15 in cycles of 28 days<br><br>Subjects in the DT cohort will receive a maximum of three cycles. Subjects in the DSRCT and ES cohort will receive unlimited cycles until disease progression, the subject begins a new anticancer treatment, withdrawal of parent/guardian/subject consent/assent, parent/guardian/subject refusal, physician decision, toxicity that cannot be managed by dose delay or dose reduction alone or the study ends for any reason.<br><br>nab-paclitaxel will be administered intravenously over approximately 30 minutes, without corticosteroid or antihistamine premedication.
**Drug Information**	• Name of the medicinal product: Nab-paclitaxel (ABRAXANE) 5 mg/mL<br>• Composition: Each vial contains 100 or 250 mg of paclitaxel formulated as albumin bound nanoparticles<br>• Pharmaceutical form: Powder for suspension for infusion
**Inclusion and Exclusion Criteria**	**Cohort 1: Desmoid tumor**<br><br>Inclusion criteria:<br><br>1. Subjects (parent or legal guardian if subject under 18 years) must voluntarily sign the informed consent form before any study test is conducted that is not part of routine subject care.<br>2. Subjects with pathologic diagnosis of deep desmoid tumor of extremities, trunk wall or head and neck region. Intra-abdominal desmoid tumor cases could be enrolled if harboring betacatenin mutation.<br>3. Subjects must be symptomatic (at least 2 points in the worst pain questionnaire of BPI) and they must be in clinical or radiological progression (according to RECIST 1.1) in the last 6 months.<br>4. Age ≥ 6 months.

5. Subjects could have received one previous chemotherapy line if the scheme was methotrexate plus vinca alkaloids. Patients who received prostaglandin inhibitors or hormone therapy are also eligible.
6. Availability of archive tumor block.
7. Measurable disease, according to RECIST 1.1 criteria.
8. Performance status ≤1 (ECOG).
9. Normal ECG values.
10. Adequate bone marrow function (hemoglobin ≥ 9 g/dL, leukocytes ≥ 3.000/mm^3, neutrophils ≥ 1.500/mm^3, platelets ≥ 100.000/mm^3). Subjects with plasma creatinine ≤ 1.6 mg/dl, transaminases ≤ 2.5 times the ULN, total bilirubin ≤ 1.25 times the ULN are acceptable.
11. Men or women of childbearing potential must use an effective method of contraception before entry into the study and throughout the same and for 6 months after ending the study treatment. Women of childbearing potential must have a negative urine or serum pregnancy test before study entry.
12. HBV and HCV serologies must be performed prior to inclusion. If HbsAg is positive it is recommended to reject the existence of replicative phase ($HbaAg^+$, DNA VHB^+) remaining at investigators' discretion the preventive treatment with lamivudine. If a potential subject is positive for anti-HCV antibodies, presence of the virus should be ruled out with a qualitative PCR, or the subject should NOT be included in the study (if a qualitative PCR cannot be performed then subject will not be able to enter the study).

Exclusion criteria:

1. Prior taxane therapy for any indication.
2. Less than 4 weeks elapsed since prior exposure to chemotherapy.
3. More than one previous chemotherapy line.
4. Subjects with desmoid tumor of abdominal cavity (abdominal wall is not an exclusion criterion).
5. Desmoid tumor with ill-defined margins.
6. Unavailability to undergo MRI.
7. Previously irradiated target lesion (if radiation dose exceeded 50 Gy).
8. Pre-existing neuropathy greater than grade 1.
9. Other active invasive malignancy requiring ongoing therapy or expected to require systemic therapy within two years. However, localized squamous cell carcinoma of the skin, basal cell carcinoma of the skin, carcinoma in situ of the cervix or other malignancies requiring only locally ablative therapy, will not result in exclusion.
10. Concomitant anticancer therapy, immunotherapy or radiation therapy within prior 4 weeks.
11. Uncontrolled intercurrent illness including but not limited to ongoing or active infection requiring IV antibiotic, symptomatic congestive heart failure, unstable angina pectoris, cardiac arrhythmia, psychiatric illness or social situations that would limit compliance with study requirements.
12. Hb < 9 g/dL.
13. Women who are pregnant or breast-feeding.
14. Known hypersensitivity to protein bound paclitaxel.
15. Any other concurrent condition that in the investigators opinion would jeopardize compliance with the protocol.
16. Known positive test for infection by human immunodeficiency virus (HIV).
17. Subjects participating in another clinical trial or receiving any other investigational product.

Cohort 2: DSRCT and ES

Inclusion criteria:

1. Subjects (parent or legal guardian if subject under 18 years) must voluntarily sign the informed consent form before any study test is conducted that is not part of routine subject care.
2. Subject diagnosed of relapsed/refractory desmoplastic small round cell tumor (DSRCT) or Ewing's sarcoma.
3. DSRCT subjects must have received at least one previous poli-chemotherapy line.
4. Ewing's sarcoma subjects must have received at least two standard chemotherapy lines.
5. Age ≥ 6 months.
6. Availability of archive tumor blocks or slides (new biopsy recommended).
7. Measurable disease, according to RECIST 1.1 criteria.
8. Performance status ≤1 (ECOG).
9. Adequate respiratory functions: FEV1 > 1L.
10. Normal ECG values.
11. Adequate bone marrow function (hemoglobin ≥ 9 g/dL, leukocytes ≥ 3,000/mm^3, neutrophils ≥ 1,500/mm^3, platelets ≥ 100,000/mm^3). Subjects with plasma creatinine ≤ 1.6 mg/dL, transaminases ≤ 2.5 times the ULN, total bilirubin ≤ 1.25 times ULN, CPK ≤ 2.5 times ULN, alkaline phosphatase ≤ 2.5 times the ULN are acceptable. If alkaline phosphatase is > 2.5 times the ULN, then the alkaline phosphatase liver fraction and/or 5' nucleotidase and/or GGT must be ≤ ULN.
12. Men or women of child bearing potential should be using an effective method of contraception before entry into the study and throughout the same and for 6 months after ending the study. Women of childbearing potential must have a negative urine pregnancy test before study entry.
13. HBV and HCV serologies must be performed prior to inclusion. If HbsAg is positive it is recommended to reject the existence of replicative phase ($HbaAg^{+}$, DNA VHB^{+}) remaining at investigators' discretion the preventive treatment with lamivudine. If a potential subject is positive for anti-HCV antibodies, presence of the virus should be ruled out with a qualitative PCR, or the subject should NOT be included in the study (if a qualitative PCR cannot be performed then subject will not be able to enter the study).
14. Prior taxane therapy for any indication is accepted.
15. > Grade 3 (intense and diffuse) expression of SPARC by immunohistochemistry.

Exclusion criteria:

1. Less than 4 weeks elapsed since prior exposure to chemotherapy.
2. Pre-existing neuropathy greater than Grade 1.
3. Other active invasive malignancy requiring ongoing therapy or expected to require systemic therapy within two years. However, localized squamous cell carcinoma of the skin, basal cell carcinoma of the skin, carcinoma in situ of the cervix or other malignancies requiring only locally ablative therapy, will not result in exclusion.
4. Concomitant anticancer therapy, immunotherapy or radiation therapy within prior 4 weeks.
5. Uncontrolled intercurrent illness including but not limited to ongoing or active infection requiring IV antibiotic, symptomatic congestive heart failure, unstable angina pectoris, cardiac arrhythmia, psychiatric illness or social situations that would limit compliance with study requirements.
6. Hb < 9 g/dL.
7. Women who are pregnant or breast-feeding.
8. Known hypersensitivity to protein bound paclitaxel.
9. Any other concurrent condition that in the investigators opinion would jeopardize compliance with the protocol.
10. Known positive test for infection by human immunodeficiency virus (HIV).
11. Subjects participating in another clinical trial or receiving any other investigational product.

**Statistical Design**	For sample size calculation, a Simon two-stage admissible design[77] is used for cohort 1 (DT), and a Simon two-stage Minimax design is used for cohort 2 (DSRCT and ES). Sample size was calculated using error rates alpha equal to 5% and beta equal to 20%. The total estimated sample size is 60 treated patients (35 for cohort 1 and 25 for cohort 2).<br><br>**<u>Cohort 1: Desmoid tumor</u>**<br><br>Option related to efficacy in ORR and/or CBR with pain improvement: 40% - Sample size: 35 subjects<br><br>Success (defined as RECIST 1.1 response and/or CBR with pain improvement) in 20% of the cases or less will be considered as unacceptable and would not warrant further investigation (null hypothesis). Therefore, the value of P0 will be taken as 20% (RECIST 1.1 response and/or CBR with pain improvement).<br><br>Success (defined as RECIST 1.1 response and/or CBR with pain improvement) in 40% of the cases or more will be considered as an acceptable result warranting further investigation of the drug in this histology (alternative hypothesis). Therefore, the value of P1 will be taken as 40% (RECIST 1.1 response and/or CBR with pain improvement).<br><br>A total of 21 eligible and treated subjects will be included in the first stage of the study. If ≤ 4 RECIST 1.1 responses and/or CBR with pain improvement are observed the trial will be stopped in this cohort with the conclusion that the drug should not be further investigated.<br><br>Else (>4 RECIST 1.1 responses and/or CBR with pain improvement are observed), subjects will continue to be accrued until 35 eligible subjects enter the study. If 12 or more successes are observed in those 35 subjects, it will be concluded that the results of this trial warrant further investigation.<br><br>**<u>Cohort 2: DSRCT at least 2nd line and Ewing's sarcoma at least 3rd line</u>**<br><br>Option related to efficacy: 30% - Sample size: 25 subjects<br><br>Success (defined as RECIST 1.1 response) in 10% of the cases or less will be considered as unacceptable, and would not warrant further investigation (null hypothesis). Therefore, the value of P0 will be taken as 10%. Success in 30% of the cases or more will be considered as an acceptable result warranting further investigation of the drug in this histology (alternative hypothesis). Therefore, the value of P1 will be taken as 30%.<br><br>A total of 16 eligible and treated subjects will be included in the first stage of the study. If 1 or less successes are observed, the trial will be stopped in this cohort with the conclusion that the drug should not be further investigated. Else (>1 successes), subjects will continue to be accrued until 25 eligible subjects enter the study. If 5 or more successes are observed in those 25 subjects, it will be concluded that the results of this trial warrant further investigation.

**Analytical Endpoints**	**<u>Cohort 1: Desmoid tumor</u>**<br><br>***Primary clinical study endpoint***<br><br>• Overall response rate (ORR) (confirmed complete response [CR] and partial response [PR]), measured using RECIST 1.1 criteria. Response criteria will be based on the baseline identification of target lesions and radiological assessments every 3 months until tumor progression.<br>• Clinical benefit rate (CBR), measured as CR+PR+SD for 3 months with improvement of pain of at least 2 points in the Brief Pain Inventory – Short Form (BPI-SF).<br><br>***Secondary clinical study endpoints***<br><br>• Pattern of radiological response according to MRI parameters (decrease of contrast enhancement in T1-Gd wi, decrease of high signal in T2-wi; increase of low signal bands; increase of the ADC score of diffusion) correlated with CBR and BPI parameters.<br>• Efficacy measured by the progression-free survival (PFS) rate assessed by median time.<br>• Variation of symptoms during the first year from trial enrollment, measured with BPI and Analgesic Quantification Algorithm (AQA).<br>• Variation of physical function during the first year from trial enrollment, assessed every 3 months.<br>• Safety profile of nab-paclitaxel, through assessment of adverse event type, incidence, severity, time of appearance, related causes, as well as physical explorations and laboratory tests. Toxicity will be graded and tabulated by using NCI-CTCAE 4.0.<br><br>**<u>Cohort 2: Desmoplastic small round cell tumor and Ewing's sarcoma</u>**<br><br>***Primary clinical study endpoint***<br><br>• Objective response rate (ORR) (confirmed complete response [CR] and partial response [PR]), measured using RECIST 1.1 criteria. Response criteria will be based on the baseline identification of target lesions and radiological assessments every 2 months until tumor progression.<br><br>***Secondary clinical study endpoint***<br><br>• Safety profile of nab-paclitaxel, through assessment of adverse event type, incidence, severity, time of appearance, related causes, as well as physical explorations and laboratory tests. Toxicity will be graded and tabulated by using NCI-CTCAE 4.0.

Procedures and assessments (Desmoid tumor)

**Protocol section reference**	**Study stages >>**	**BEFORE TREATMENT**	**TREATMENT**					**END OF TREATMENT[1] (+/- 7 days)**	**FOLLOW-UP (+/- 7 days)**
		**Screening (≤ 28 days of start of treatment)**	**Every cycle (up to 3 cycles)**						
			**Week 1 (Day 1)**	**Week 2 (Day 8)**	**Week 3 (Day 15)**				
**7.2**	**Subject information and consent**								
	Subject information	X (prior to screening)							
	Informed consent	X (prior to screening)							
**7.3**	**Eligibility and enrollment procedures**								
	Screening number assignment	X							
	Central pathology review	X							
	Eligibility confirmation	X							
	Subject enrollment	X							
**7.4**	**Clinical assessments**								
	Demographic data and medical history	X							
	ECOG	X	X					X	
**7.5**	**Laboratory assessments (+/- 3 days)**								
	Biochemistry[2]	X	X[3]	X	X			X	
	Hematology[4]	X	X[5]	X	X			X	
	Coagulation tests[6]	X							
	Thyroid function test	X							
	Pregnancy test	X							
	HBV/HCV serology	X							
**7.6**	**Safety assessments**								
	Physical examination	X	X					X	X
	Vital signs[7]	X	X	X	X			X	
	Adverse events[8]	X	X	X	X			X	X
	Concomitant medication	X	X	X	X			X	
	ECG[9]	X							
	LVEF	X						X	
**7.7**	**Efficacy assessments**								
	MRI (including diffusion and perfusion)	X						X[10]	X
	Central radiology review							X	
**7.8**	**Pain assessments[11]**								
	Brief Pain Inventory (BPI)	X	X	X	X			X	X
	Analgesic Quantification Algorithm (AQA)	X	X	X	X			X	X
**7.9**	**Treatment management**								
	Treatment administration[12]		X	X	X				
	Treatment compliance				X				
**7.10**	**Biological sample collection**								
	Tumor block collection	X							
	Blood sample collection	X						X	
**7.11**	**Survival**								
	Follow-up visits								X[13]

1. End of treatment for desmoid tumor cohort is on day 84 (after 3 cycles of 28 days).
2. Biochemistry: ALT, AST, bilirubin, creatinine, CPK, GGT, ALP, glucose, albumin, total protein, LDH, urea, sodium, potassium, calcium, magnesium, inorganic phosphate.
3. Biochemistry must be performed on day 1 if not done within one week prior to treatment initiation.
4. Hematology: hemoglobin, WBC, RBC, lymphocytes, platelets, neutrophils, hematocrit.
5. Hematology must be performed on day 1 if not done within one week prior to treatment initiation.
6. Coagulation (partial thromboplastin time, fibrinogen and INR) may be repeated if clinically indicated.
7. Vital signs include: blood pressure, heart rate and temperature.
8. AEs must be collected from the signature of the informed consent, during the entire treatment and up to 28 days after last administration of study drug.
9. ECG may be repeated if clinically indicated.
10. Tumor assessment should be performed if end of treatment was not due to radiological progression, or if the last radiological imaging was performed more than 2 months ago.
11. BPI and AQA only for desmoid tumor cohort. At baseline, days 1, 8, and 15, at the end of treatment, and then every 3 months for 3 years.
12. Nab-paclitaxel will be administered on days 1, 8, and 15 of each cycle.
13. Every 3 months during the first year and every 6 months for 2 additional years (maximum follow-up within the study).

Procedures and assessments (Desmoplastic small round cell tumor and Ewing's sarcoma)

Protocol section reference	Study stages >>	BEFORE TREATMENT	TREATMENT					END OF TREATMENT[1] (+/- 7 days)	FOLLOW-UP (+/- 7 days)
		Screening (≤ 28 days of start of treatment)	Every cycle (until progression)				Every 8 weeks		
			Week 1 (Day 1)	Week 2 (Day 8)	Week 3 (Day 15)				
**7.2**	**Subject information and consent**								
	Subject information	X (prior to screening)							
	Informed consent	X (prior to screening)							
**7.3**	**Eligibility and enrollment procedures**								
	Screening number assignment	X							
	Central pathology review	X							
	Eligibility confirmation	X							
	Subject enrollment	X							
**7.4**	**Clinical assessments**								
	Demographic data and medical history	X							
	ECOG	X	X					X	
**7.5**	**Laboratory assessments (+/- 3 days)**								
	Biochemistry[2]	X	X[3]	X	X			X	
	Hematology[4]	X	X[5]	X	X			X	
	Coagulation tests[6]	X							
	Thyroid function test	X							
	Pregnancy test	X							
	HBV/HCV serology	X							
**7.6**	**Safety assessments**								
	Physical examination	X	X					X	X
	Vital signs[7]	X	X	X	X			X	
	Adverse events[8]	X	X	X	X		X	X	X
	Concomitant medication	X	X	X	X		X	X	
	ECG[9]	X							
	LVEF	X						X	
**7.7**	**Efficacy assessments**								
	MRI/Thoracic-abdominal CT scan	X					X	X[10]	X
	Central radiology review							X	
**7.9**	**Treatment management**								
	Treatment administration[11]		X	X	X				
	Treatment compliance				X				
**7.10**	**Biological sample collection**								
	Tumor block collection	X						X	
**7.11**	**Survival**								
	Follow-up visits								X[12]

1. DSRCT and ES cohort continues until progression.
2. Biochemistry: ALT, AST, bilirubin, creatinine, CPK, GGT, ALP, glucose, albumin, total protein, LDH, urea, sodium, potassium, calcium, magnesium, inorganic phosphate.
3. Biochemistry must be performed on day 1 if not done within one week prior to treatment initiation.
4. Hematology: hemoglobin, WBC, RBC, lymphocytes, platelets, neutrophils, hematocrit.
5. Hematology must be performed on day 1 if not done within one week prior to treatment initiation.
6. Coagulation (partial thromboplastin time, fibrinogen and INR) may be repeated if clinically indicated.
7. Vital signs include: blood pressure, heart rate and temperature.
8. AEs must be collected from the signature of the informed consent, during the entire treatment and up to 28 days after last administration of study drug.
9. ECG may be repeated if clinically indicated.
10. Tumor assessment should be performed if end of treatment was not due to radiological progression, or if the last radiological imaging was performed more than 2 months ago.
11. Nab-paclitaxel will be administered on days 1, 8, and 15 of each cycle.
12. Every 2 months.

2. INTRODUCTION

2.1 Background and scientific rationale

2.1.1 Desmoid tumors

Sarcomas are rare tumors that include more than 60 varieties. Two groups are analyzed in this study: desmoid and small round cell tumours. Desmoid tumors (DTs) are benign and usually deep-seated. They are originated by myofibroblastic neoplasms that grow slowly and arise from musculo aponeurotic stromal components. DTs are rare, < 3% of all soft tissue tumors and can be diagnosed at any age but are more common in ages between 10 and 40. DTs can developed at any site of the body; however, the abdominal wall and soft tissues of the extremities, shoulder, neck, and chest wall are the most common sites of development. Due to rare diagnosis, variety of anatomical presentation, and limited clinical trials performed up to now, treatment selection can be extremely difficult.

Molecular aspects of DTs

DTs are locally aggressive fibroblastic neoplasms usually arising from deep tissues, with local infiltrating behavior and without metastatic capacity. They are also known as aggressive fibromatosis, since the local invasiveness of this tumor can sometimes derive in relevant clinical problems. Examples of these latter events are neurovascular bundle involvement or intestinal obstruction for intra-abdominal presentations. DTs are rare with an estimated incidence of 2-4 per million and year[1,2]. Most DTs arise sporadically, with some preference in limbs, shoulders, abdominal wall, neck and chest wall. In some occasions there is a previous trauma or pregnancy as related causative event. Other DTs can be associated to cancer syndromes as familial adenomatous polyposis (FAP) which represents around 7% of all DTs. In this context, DTs can be a life-threatening condition usually presented after prophylactic colectomy since the surgical trauma would act as trigger factor for DT[3]. No apparent differences have been found in those DT diagnosed in children compared with those in adults[4].

The etiology of DTs is likely multifactorial since endocrine, genetic or traumatic factors have been advocated in their development. In abdominal wall is typical the presentation in women during pregnancy or puerperium period or after traumatic scars. In FAP the probability of developing DTs is about 10-15%, often associated to germline mutation of the adenomatosis polyposis coli (*APC*) gene. Sporadic DTs are also associated with somatic mutations in either β-catenin (*CTNNB1*) or *APC* genes and up to 85% of these sporadic lesions carry mutations in either *CTNNB1* or *APC*[5].

Histopathologically, DTs are lesions poorly circumscribed with infiltration of the surrounding soft-tissue structures. There is typically a proliferation of spindle cells arranged in ill-defined fascicles (in long fascicles or whirling patterns) with tumor cellularity set in a collagenous stroma appearance containing prominent blood vessels. Cells lack cytological atypia with variable mitotic rate.

DTs are usually positive for vimentin and show variable reaction with smooth muscle actin. Nuclear β-catenin immunostaining is expressed in about 67%-80% of cases of DTs[6] but it is not specific of this entity since it is observed, for instance, in 30% of low grade myofibroblastic sarcomas and 22% of solitary fibrous tumors.

Sporadic DTs are commonly associated with somatic mutations of β-catenin gene (*CTNNB1)* and these mutations involve usually codons 41 or 45 of exon 3 of this gene. Studies are controversial regarding the prognostic correlation of the different mutation sites[5,7]. In both studies, the presence of mutations implies more risk of recurrence.

Both, *CTNNB1* as well as *APC* are genes belonging to Wnt signaling pathway and, in the DT context, there is failure in degradation of β-catenin protein in the cytoplasm. The accumulated protein after activating mutation of *CTNNB1* oncogene, in chromosome 3, is then transported to the nucleus where acts as transcription factor by binding to T-cell factor and targeting genes involved in proliferation. In a similar manner, the *APC* tumor suppressor gene, which maps in chromosome 5, forms a molecular complex with several molecules including β-catenin, microtubulin, axin and GSK3β. Thus *APC* deactivates β-catenin

reducing Wnt signal transduction. *APC* mutations result in the stabilization of the β-catenin and consequently an accumulation of this protein does occur[8,9].

Cyclooxygenase-2 (*COX-2*) is one of the target genes of β-catenin and encodes the protein with enzymatic functions involved in the production of prostaglandins[10]. This fact could explain why prostaglandin inhibitors as sulindac or indomethacin can induce responses in DTs[11]. Activation of *COX-2* contributes to tumorigenesis by inhibiting apoptosis, stimulating angiogenesis and invasiveness. However, most of the reported activity of COX-2 inhibitors is limited to short series and usually obtaining stabilizations which represents a doubtful positive outcome in the context of DT, for which there is a clear trend towards stabilization without any treatment. In other tumors where COX-2 is widely expressed, as bladder carcinoma, there was no significant correlation between COX-2 expression and recurrence or survival in invasive tumors treated with paclitaxel as adjuvant treatment. However, MVD estimated by CD105 resulted in a better prognostic biomarker suggesting that the prognostic significance of β-catenin might be independent of COX-2 expression[12]. Similarly, a metronomic scheme of paclitaxel could play some role in metastatic melanoma. In this context, there is robust evidence that COX-2 is related to tumor progression. As taxanes lead to activation of COX-2, other mechanisms, such as its antiangiogenic properties, are advocated to explain the potential activity[13].

Strong rationale has related the antiangiogenic effect of taxanes with the inhibition of Hypoxia-inducible factor-1 (HIF-1α). This could be especially relevant in hormone-refractory prostate cancer, which is known to overexpress HIF-1α as well as HIF-target genes, where docetaxel is the election drug[14]. Interestingly, the antimitotic effect of taxanes does not seem to be the key of survival improvement since the average of mitotic rate in this scenario is around 2%. Moreover, rigorous analyses indicate that β-catenin can enhance HIF-1-mediated transcription, thereby promoting cell survival[15]. It has been recognized that mTOR inhibitors can reduce the process of angiogenesis through inhibition HIF-1α[16]. In fact, a pilot trial is now being conducted with sirolimus in a phase I–II DT study by the Maine Medical Center (Clinicaltrials.gov identifier: NCT01265030).

Other target gene of β-catenin is metalloproteinase-7 (*MMP-7*), which has been reported to play an important role in tumor progression[17]. This makes sense since DTs show locally aggressive growth and may need the cooperation of enzymes responsible of extracellular matrix degradation. Therefore, matrix metalloproteases can be crucial for this aim. Overexpression by immunohistochemistry of MMP-7 in DT has been published, supporting the idea that MMP-7 could be a potential target in this tumor[18]. Interestingly, taxanes have demonstrated inhibitory properties in MMP. Therefore, this biomarker family had to be taken into account[19].

Furthermore, β-catenin seems to have an influence on angiogenesis and authors have proven statistically significant correlation between nuclear expression of β-catenin and overexpression of VEGFR in all DTs[20]. Antiangiogenic effects of taxanes in cancer are well recognized, especially at low doses[21]. This inhibitory effect could be driven either through main angiogenic cytokines VEGF and bFGF or by preventing endothelial cell angiogenic processes.

Clinical considerations in DT

Extra-abdominal desmoid tumors do not usually associate with mortality but with morbidity. These tumors have no metastatic potential even though they can be present as multicenter, more frequently as diachronic, in limbs. In spite of the lack of metastatic involvement, the local aggressiveness of DTs can cause severe damage with invasion of neurovascular bundle or other vital structures. The majority of subjects presents a painless swelling but in some circumstances there is moderate to severe pain, muscular contractures and dysfunction. The overlying skin does not show to be affected in the exploration. DT exhibits an unpredictable behavior and for each case the multidisciplinary team has to personalize the therapeutic approach in accordance with biological growth rate. Spontaneous regression has been registered in these tumors[22]. Radiological investigation includes ultrasonography, showing hypoechoic and poorly defined soft tissue mass. Computed tomography is also of limited value due to similar attenuation between muscle and DT, intravenous contrast usually enhances the lesion. Magnetic resonance imaging is the test of choice. Lesions on T1-weighted imaging are homogenously isointense with a high heterogeneous signal on T2-weighted imaging. Contrast administration results in a significant signal enhancement[23]. Hypocellular DTs have a decreased signal on MRI.

Therapeutic options are diverse in DT and, in the recent years, a non-surgical approach has been promoted by oncologic surgeons from highly experienced institutions[24,25]. Difficulties to render microscopic free margins due to infiltrative nature of DTs, controversial predictive value of R1 vs R0 resections, a trend toward stabilization with "wait and see" approach, among other reasons, have been advocated for conservative non-surgical approach. Radiotherapy has been recommended for both: adjuvant setting and as upfront treatment. In a retrospective survey of 110 subjects, the addition of radiotherapy was an independent factor for local recurrence[26]. High rates of radiation-related complications were associated with doses > 56 Gy[27].

Regarding systemic treatments, various medical treatment options have been investigated, mostly in retrospective case reports. There is a lack of randomized trials and controlled studies in DTs have been very scarce. Antihormonal therapy such as tamoxifen or NSAIDs, sulindac or indomethacin, have been advised for DT. The number of responses range widely and there is no consensus on the dose of tamoxifen (standard or high dose). Activity with NSAIDs has been reported as high as 50% of partial responses, but the majority of responders experienced a delayed response with a mean time of 24 months[28].

Chemotherapy has been offered to DT subjects in the context of more aggressive and commonly more symptomatic tumors. Weekly administration of methotrexate and vinblastine is the most frequent regimen used in DT. The largest series communicated at least stable disease in 18 out of 27 subjects[29]. However this scheme takes a long time to respond, normally several months. Thus, this regimen becomes toxic over time with peripheral neuropathy, bone marrow toxicity or even pneumonitis secondary to methotrexate. One alternative to ameliorate the toxicity is to use vinorelbine instead vinblastine. In any case, the number of cycles required for this regimen seems to be exaggeratedly high in the arena of a benign condition.

Anthracycline-based therapy gives greater benefit[30] but with Grade 3 neutropenia in half of the subjects. Pegylated liposomal doxorubicin at a dose of 50 mg/m^2 every 4 weeks was reported to have significant activity with 4 partial responses and 7 stabilizations out of 12 subjects. Nevertheless, 6 out of 11 subjects required dose reduction because of toxicity[31].

Targeted therapies such as imatinib have reported some responsive cases that could be related to drop in serum *PDGFR-β* values. In a series of 40 subjects treated with imatinib there were 9% of partial responses and 83% of stable disease with 6 months of PFS rate of 74%[32]. However, imatinib is not licensed for this indication.

Taxanes in DT

There are no reports of the use of taxanes in DT to the best of our knowledge. As a personal experience, a subject with metastatic breast carcinoma and DT of abdominal wall showed an early response of DT (80%) to weekly paclitaxel. Taking together molecular reasons, taxanes could be active in DT targeting HIF-1α, MMPs or VEGFR. Regarding clinical arguments, low doses of taxanes could add value into the systemic options of treatment since we might expect much less bone marrow and cardiac toxicity compared with anthracycline-based regimens. On the other hand, we hypothesize that the time to response could be considerably shorter compared with regimens based on alkaloid of vinca.

Among taxanes, nanoparticle albumin-bound paclitaxel (nab-paclitaxel) is a novel formulation of paclitaxel that does not require solvents for its formulation. In breast cancer there is a considerable accrued experience with this compound. In the pivotal phase III trial, nab-paclitaxel every 3 weeks was superior to paclitaxel in terms of response and time to progression[33]. In another phase II randomized trial[34], weekly (3 weeks every 4 weeks) nab-paclitaxel at 150 mg/m^2 was the most active and safe regimen compared with docetaxel 100 mg every 3 weeks, nab-paclitaxel 300 mg/m^2 every 3 weeks or weekly nab-paclitaxel 100 mg/m^2.

2.1.2 Desmoplastic small round cells and Ewing's sarcoma

"Small round cell tumors" have all a similar histological description. This enormous group contains desmoplastic small round cell tumors (DSRCT) as well as neuroblastomas, Wilms' tumors, rhabdomyosarcomas, Ewing's sarcomas (ES), primitive neuroectodermal tumors, small cell

osteosarcomas, poorly differentiated synovial cell sarcomas and lymphomas. They are all characterized by sheets of small cells with round nuclei[35]. Only DSRCT and ES will be considered in this study.

DSRCT presents with multifocal tumors. Because of the rarity of the tumor and the unusually aggressive presentation, treatment is challenging and has not been standardized. ES usually develops in bone (85% of cases) and presents characteristic molecular alterations that help in diagnosing and selecting the best treatment[36].

The therapeutic advantages on this type of tumors have come through a multidisciplinary approach: preservation surgery, precise radiotherapy, implementation of translational research, new challenges on pathologic diagnostics and introduction of new therapeutic agents.

DSRCT was described as a separate entity by Gerald *et al*. in 1991, in clear distinction with all the other small-round blue cell tumors. DSRCT is clinically characterized by male predominance (>5:1); primary (often exclusive) intra-abdominal location associated with multiple and smaller peritoneal implants, with a predilection for pelvic region; initial response to chemotherapy followed by uncontrollable tumor relapse and high mortality rate.

From the histopathological standpoint, DSRCT is characterized by peritoneal implants commonly invading local structures like bladder, colon, small bowel; clusters of small cells separated by a consistent and prominent cellular ("desmoplastic") stroma; growth pattern always invasive but of a pushing nature; broad positive immunohistochemical profile showing co-expression of epithelial markers (EMA and keratin), neuronal markers (NSE), and mesenchymal markers (vimentin and desmin)[37].

Early in 1995 the MSKCC team led by Gerald, Ladanyi and Rosai precisely described the characteristics of the genomic breakpoint and fusion transcripts involved in the EWS-WT1 gene fusion of DSRCT[38]. They proved that DSRCT represents the third tumor type described to be associated with a chromosomal translocation involving the EWS gene and the only tumor ever described to be associated with translocation of WT1. The chimeric transcript of two different tumor-associated genes was clearly implicated in the genesis of DSRCT. The translocation results in chimeric products containing the amino-terminal domain of EWS fused to the nucleic acid binding domain of WT1. The age of presentation, male predominance, and primitive appearance of the neoplastic cells in DSRCT were all reminiscent of the Ewing family of tumors. On the other hand, WT1 was known to be expressed in embryonic structures derived from the intermediate mesoderm, mesenchyme lining the coelom and mesenchyme of the primitive gonad. WT1 is a transcription factor intimately associated with a particular period in normal development the alteration of which may contribute to tumor formation in the primitive cells expressing the gene. An histogenetic hypothesis for DSRCT was suggested: DSRCT arises in the primitive mesenchyme of the coelomic cavities or gonads[39]. Similar to other small-round cell tumors of young people, DSRCT is an alkylator-sensitive and dose-responsive tumor. However, intensive chemotherapy or myeloablative doses of alkylating agents (thiotepa) did not result in complete pathologic responses of large masses. Hence, aggressive surgery to remove bulk tumor was found to be a critical component of a curative strategy[40]. The median survival time at the original report was 19 months and for those achieving CR, the median follow-up was 22 months. It was later found that durable responses were rare at long term. A review from the MSKCC group in 2005 of 66 subjects diagnosed as having DSRCT, from 1972 to 2003, showed a 3-year survival of 55% in those receiving chemotherapy, surgery and radiotherapy, and 27% when all 3 modalities were not used. Three- and five-year survivals were 44% and 15%, respectively[41]. A more recent report of the MSKCC group with 31 subjects managed from 1992 to 2011 showed 3-year OS and PFS rates of 50% and 24%, respectively[42].

Expression of EWS-WT1 induced the expression of platelet derived growth factor-A (PDGFA). Native PDGFA was not induced by WT1 indicating target specificity by the EWS-WT1 fusion transcript. Primary DSRCTs were shown to express high levels of PDGFA, however PDGFA was absent in tumors expressing WT1 and in Ewing's sarcomas expressing EWS-FLI, showing the specificity of PDGFA expression correlating with the tumorigenic activity of EWS-WT1[43]. PDGFA is a secreted growth factor that acts as a potent mitogen and chemo-attractant for fibroblasts and endothelial cells. This finding is relevant because the histology of DSRCT is remarkable for the presence of a profuse stromal reaction. The potential growth advantage conferred by this stromal reaction, including vascular recruitment, is consistent with DSRCT specimens demonstrating active neoangiogenesis in the connective tissues surrounding tumor cells.

As in Ewing's sarcoma, the benefit of high-dose chemotherapy and autologous hemopoietic stem cell transplant is controversial, at best. In 2010 the Italian group reported their experience with 14 DSRCT subjects treated with three consecutive intensified chemotherapy combinations followed by stem cell rescue[44]. With a median follow-up of 27 months only 3 subjects remained alive without evidence of disease. The 3-year EFS and OS rates were 15.5 and 38.9%, respectively. This experience corroborated the initial description by the MSKCC group of the lack of improvement using myeloablative doses of alkylating agents like thiotepa[45]. In 2008, the MSKCC group showed that hybrid PET/CT had superior overall sensitivity and specificity to PET or CT alone in DSRCT[46]. Further retrospective review of 7 subjects with DSRCT who were imaged by FDG-PET and CT at diagnosis and after 3 cycles of chemotherapy showed that FDG-PET did not always correlate with response measurement by CT. A greater decrease in metabolic activity as compared with size was seen in all subjects[47].

The last clinical trials reported for DSRCT subjects include a phase II clinical trial of imatinib for KIT and/or PDGFR alpha-expressing tumors like DSRCT, published in 2010[48]. Seven DSRCT subjects were enrolled and one subject with 3+/4+ PDGFR alpha and 3+/4+ KIT expression had a partial response. In 2011 initial observations of activity with trabectedin (Yondelis®) in adults with DSRCT were reported[49]. In 2012 a phase I trial evaluating the 24-hour infusion of trabectedin in children and adolescents with refractory solid tumors reported one DSRCT with stable disease[50]. Finally, in 2013 the clinical activity of sunitinib in 8 subjects with DSCRT was reported[51].

Nab-paclitaxel has recently shown preclinical activity against pediatric solid tumors such as rhabdomyosarcoma and neuroblastoma[52]. However, the activity of this drug against other solid tumors characteristic of the young, including DSRCT and Ewing's sarcoma, remains unexplored. We hypothesize that the antitumor activity and stroma-disrupting effects of nab-paclitaxel shown in pancreatic ductal adenocarcinoma (Alvarez et al, British Journal of Cancer, 2013) will be replicated in DSRCT. Also, because albumin internalizes in cells via Gp60 receptors and caveolin-mediated endocytosis, there exists a relationship between caveolin expression and malignancy of Ewing's sarcoma[53,54]. We hypothesize that nab-paclitaxel will be active in Ewing's sarcoma.

In 2009 Mora *et al.* published the first series of pediatric subjects treated with gemcitabine and docetaxel (GEMDOX) as relapse therapy[55]. This series was notable for six subjects with Ewing's sarcoma and reported a very high complete response rate of 40%. GEMDOX drugs, not routinely used to treat pediatric malignancies, seemed to rescue relapsed sarcoma subjects, induce tumor remission and, as maintenance chemotherapy, keep disease under control for prolonged periods of time (median duration of responses 10 months), even after 2 or more relapses. On the basis of these results, evaluation in a formal phase 2 setting from GEIS for primary high-risk Ewing's sarcoma subjects is almost complete (Clinicaltrials.gov identifier NCT01696669). More recently, Rapkin L *et al* showed an objective response in 2 of 18 pediatric sarcoma subjects (11%), both with rhabdomyosarcoma, and SD in another 7 of 18 subjects (39%)[56]. Importantly, while concurrent therapy was given in five subjects, both objective responses and four of the seven subjects with SD occurred in subjects who received GEMDOX alone. In conclusion, the combination of gemcitabine and paclitaxel has shown clinical benefit for relapsed sarcoma subjects including Ewing family of tumors and DSRCT. Furthermore, histological and biological features of EWS rearranged tumors suggest that albumin-bound paclitaxel particles (nab-paclitaxel [ABRAXANE], Celgene) could be more active than paclitaxel.

Five subject-derived xenograft (PDX) Ewing's sarcoma models named with the codes HSJD-ES-001, HSJD-ES-003, HSJD-ES-004, HSJD-ES-006 and HSJD-ES-008, established from subject biopsies at Hospital Sant Joan de Déu (HSJD, Barcelona, Spain) were used to evaluate the activity of nab-paclitaxel in vivo. Clinical characteristics of subjects and molecular details of the tumor models are available from investigators at HSJD. Nod-Scid mice (Harlan, Barcelona, Spain) were used for initial engraftment and further passages were performed in athymic nude mice (Harlan). For the survival studies, nude mice bearing subcutaneous 200-500 mm^3 tumors in both flanks were randomized in 2 groups of 5 mice. One group received 50 mg/kg nab-paclitaxel intravenous on days 0, 7 and 14; a second group received no treatment as controls. Mice were sacrificed when the tumor diameter reached 1.5 cm^3 and the study was finalized at day 80 post-treatment. To study the activity of the regimens we evaluated tumor response at the end of treatment (day 21) and animal survival until the end of the study (day 80). Tumor response was evaluated as described[57]. Animal survival was defined as the time interval between the initial date of treatment and the date in which 1.5 cm^3 tumor volume was reached. Median survivals of the animals were calculated using Kaplan-Meier curves and the log-rank test was used for statistical comparisons between

each treatment group and the control group. The levels of SPARC in the Ewing PDX models were assessed by western blot and immunohistochemistry.

Nab-paclitaxel achieved complete response (CR) in 100% of the tumors (n=5, each) of 3 (models HSJD-ES-001, -003 and -008) out of the 5 PDX models. In model HSJD-ES-006, nab-paclitaxel treatment achieved partial response (PR) or stable disease (SD) in 5 and 7 tumors, respectively, out of 12 treated tumors. Model HSJD-ES-004 was resistant to nab-paclitaxel treatment and tumor progressed during treatment.

Efficacy of nab-paclitaxel was correlated with the protein levels of SPARC. Low SPARC expression was found in the PDX model not responding to nab-paclitaxel (HSJD-ES-004) and in the PDX model with partial response or stable disease at day 21 (HSJD-ES-006). In contrast, SPARC levels were high in the 3 PDX models (HSJD-ES-001, -003 and -008) in which maintained CR was achieved. By immunohistochemistry, the preliminary data show high expression of SPARC in the responding model HSJD-ES-003, as compared to low expression in the non-responding model HSJD-ES-004.

Our results correlating SPARC expression in Ewing's sarcoma PDX and nab-paclitaxel efficacy are in contrast to a recently published experience from the PPTP program (COG) showing lack of correlation between SPARC expression and nab-paclitaxel activity in a panel of 8 Ewing's sarcoma xenografts[58]. Current work in the HSJD laboratory is expanding the results to new PDX models to confirm our preliminary data. At HSJD we are working to evaluate paclitaxel distribution in tumors with high and low SPARC expression. We will use our recently published technique to address such question[59].

In conclusion, the preliminary data obtained at HSJD in 5 Ewing's sarcoma PDX models suggest high activity of nab-paclitaxel in Ewing's sarcoma cases expressing high-levels of SPARC. The use of SPARC as a biomarker to select subjects for nab-paclitaxel in Ewing (and DSRCT) could potentially be relevant for a better design of clinical trials and personalized treatments using nab-paclitaxel.

2.2 Nab-paclitaxel

nab-paclitaxel (ABI-007, ABRAXANE® for Injectable Suspension [Celgene Corporation, Summit, New Jersey, United States]) is a human serum albumin bound nanoparticle formulation of paclitaxel with a mean particle size of approximately 130 nanometers. Nab-Paclitaxel has been developed to improve the therapeutic index of paclitaxel; the chemotherapeutic effect is enhanced by exploiting endogenous transport pathways to deliver higher doses of paclitaxel to the tumor[60], while at the same time the toxicities associated with conventional solvent-based paclitaxel formulations using a Cremophor® EL (BASF, Ludwigshafen, Germany) and ethanol vehicle are reduced.

nab-paclitaxel is bound to albumin in amorphous state and, unlike conventional solvent-based paclitaxel formulations where micellar entrapment is observed[61,62,63], has linear pharmacokinetic (PK) characteristics. Based on these pharmacokinetic properties, the dose and short infusion time, an increase of the maximum concentration (C_{max}) of free paclitaxel up to 10-fold greater than with conventional solvent-based paclitaxel has been reported in the literature[64]. The transport of paclitaxel across the endothelium is enhanced through albumin receptor mediated transcytosis, and the delivery of paclitaxel to tumors may be enhanced by binding of the albumin-bound paclitaxel to interstitial albumin binding proteins, such as secreted protein acidic and rich in cysteine (SPARC; also known as osteonectin)[60]. nab-paclitaxel is not known to cross the blood-brain barrier.

Although it has been hypothesized that SPARC expression may result in an increased concentration of nab-paclitaxel in tumors due to its albumin-binding ability, and may play a role in the enhanced antitumor activity, clinical studies remains conflicting[65,66,67,68] and therefore there is not sufficient data supporting the relationship of SPARC expression to clinical outcomes of nab-paclitaxel treatment.

Type of solid tumors had no significant effect on paclitaxel pharmacokinetics in subjects who received nab-paclitaxel. Ethnic origin had no discernible effect upon PK parameters according to the studies conducted in Western countries, Japan and China.

The novel nab-paclitaxel nanoparticles conferred the ability to achieve a higher maximum tolerated dose (MTD) based on every 3-weeks dosing: 300 mg/m^2 for nab-paclitaxel (DM97123) versus 175 mg/m^2 for conventional solvent-based paclitaxel[69]. The use of albumin also enables nab-paclitaxel to be given in a shorter, more convenient infusion time of 30 minutes compared with 3 hours to 24 hours with conventional solvent-based paclitaxel. Nab-Paclitaxel is given without steroid and antihistamine premedication, which is required for conventional solvent-based paclitaxel to prevent solvent-related hypersensitivity reactions[70]. Cremophor EL has been shown to leach plasticizers, specifically di (2-ethylhexyl) phthalate (DEHP), from polyvinyl chloride (PVC) bags and polyethylene-lined tubing[71,72,73,74,75,76].

Although no controlled epidemiologic toxicity studies have been conducted in humans exposed to DEHP, severe effects (eg, carcinogenicity, cardiopulmonary toxicity, hepatotoxicity, and nephrotoxicity) have been observed in experimental models. The Taxol product information instructs users to prepare, store, and administer solutions in glass, polypropylene, or polyolefin containers; non-PVC-containing infusion sets (eg, those with polyethylene lining) should be used[70]. By comparison, standard tubing and intravenous (IV) bags may be used for the IV administration of nab-paclitaxel[61,69].

Please refer to the Investigator Brochure for detailed information concerning the available pharmacology, toxicology, drug metabolism, clinical studies and adverse event (AE) profile of the study drug.

3. CLINICAL STUDY OBJECTIVES

Cohort 1: Desmoid tumor

Primary clinical study objectives

- To determine the overall response rate (ORR) according to RECIST 1.1 and/or clinical benefit rate (CBR) at 3 months with pain improvement of at least 2 points in the Brief Pain Inventory – Short Form (BPI-SF).

Secondary clinical study objectives

- To define the pattern of radiological response according to MRI parameters and to correlate it with CBR and Brief Pain Inventory (BPI) parameters.
- To estimate the efficacy of nab-paclitaxel as measured by the progression-free survival (PFS) assessed by median time.
- To analyze the variation of symptoms during the first year from trial enrollment in accordance with BPI and Analgesic Quantification Algorithm (AQA).
- To analyze the variation of physical function during the first year from trial enrollment.
- To evaluate the safety profile of nab-paclitaxel according to CTCAE 4.0.

Cohort 2: Desmoplastic small round cell tumor and Ewing's sarcoma

Primary clinical study objective

- To determine the objective response rate (ORR) in subjects with desmoplastic small round cell tumor and Ewing's sarcoma, using RECIST 1.1 criteria.

Secondary clinical study objectives

- To evaluate the safety profile of nab-paclitaxel according to CTCAE 4.0.

4. SELECTION OF SUBJECTS

4.1 Inclusion and exclusion criteria for cohort 1: DT

4.1.1 Inclusion criteria DT

1. Subjects (parent or legal guardian if subject under 18 years) must voluntarily sign the informed consent form before any study test is conducted that is not part of routine subject care.
2. Subjects with pathologic diagnosis of deep desmoid tumor of extremities, trunk wall or head and neck region. Intra-abdominal desmoid tumor cases could be enrolled if harboring betacatenin mutation.
3. Subjects must be symptomatic (at least 2 points in the worst pain questionnaire of BPI) and they must be in clinical or radiological progression (according to RECIST 1.1) in the last 6 months.
4. Age ≥ 6 months.
5. Subjects could have received one previous chemotherapy line if the scheme was methotrexate plus vinca alkaloids. Patients who received prostaglandin inhibitors or hormone therapy are also eligible.
6. Availability of archive tumor block.
7. Measurable disease, according to RECIST 1.1 criteria.
8. Performance status ≤1 (ECOG).
9. Normal ECG values.
10. Adequate bone marrow function (hemoglobin ≥ 9 g/dL, leukocytes ≥ 3.000/mm^3, neutrophils ≥ 1.500/mm^3, platelets ≥ 100.000/mm^3). Subjects with plasma creatinine ≤ 1.6 mg/dl, transaminases ≤ 2.5 times the ULN, total bilirubin ≤ 1.25 times the ULN are acceptable.
11. Men or women of childbearing potential must use an effective method of contraception before entry into the study and throughout the same and for 6 months after ending the study treatment. Women of childbearing potential must have a negative urine or serum pregnancy test before study entry.
12. HBV and HCV serologies must be performed prior to inclusion. If HbsAg is positive it is recommended to reject the existence of replicative phase ($HbaAg^+$, DNA VHB^+) remaining at investigators' discretion the preventive treatment with lamivudine. If a potential subject is positive for anti-HCV antibodies, presence of the virus should be ruled out with a qualitative PCR, or the subject should NOT be included in the study (if a qualitative PCR cannot be performed then subject will not be able to enter the study).

4.1.2 Exclusion criteria DT

1. Prior taxane therapy for any indication.
2. Less than 4 weeks elapsed since prior exposure to chemotherapy.
3. More than one previous chemotherapy line.
4. Subjects with desmoid tumor of abdominal cavity (abdominal wall is not an exclusion criterion).
5. Desmoid tumor with ill-defined margins.
6. Unavailability to undergo MRI.
7. Previously irradiated target lesion (if radiation dose exceeded 50 Gy).
8. Pre-existing neuropathy greater than grade 1.
9. Other active invasive malignancy requiring ongoing therapy or expected to require systemic therapy within two years. However, localized squamous cell carcinoma of the skin, basal cell carcinoma of the skin, carcinoma in situ of the cervix or other malignancies requiring only locally ablative therapy, will not result in exclusion.
10. Concomitant anticancer therapy, immunotherapy or radiation therapy within prior 4 weeks.
11. Uncontrolled intercurrent illness including but not limited to ongoing or active infection requiring IV antibiotic, symptomatic congestive heart failure, unstable angina pectoris, cardiac arrhythmia, psychiatric illness or social situations that would limit compliance with study requirements.
12. Hb < 9 g/dL.
13. Women who are pregnant or breast-feeding.
14. Known hypersensitivity to protein bound paclitaxel.
15. Any other concurrent condition that in the investigators opinion would jeopardize compliance with the protocol.
16. Known positive test for infection by human immunodeficiency virus (HIV).

17. Subjects participating in another clinical trial or receiving any other investigational product.

4.2 Inclusion and exclusion criteria for cohort 2: DSRCT and ES

4.2.1 Inclusion criteria

1. Subjects (parent or legal guardian if subject under 18 years) must voluntarily sign the informed consent form before any study test is conducted that is not part of routine subject care.
2. Subject diagnosed of relapsed/refractory desmoplastic small round cell tumor (DSRCT) or Ewing's sarcoma.
3. DSRCT subjects must have received at least one previous poli-chemotherapy line.
4. Ewing's sarcoma subjects must have received at least two standard chemotherapy lines.
5. Age ≥ 6 months.
6. Availability of archive tumor blocks or slides (new biopsy recommended).
7. Measurable disease, according to RECIST 1.1 criteria.
8. Performance status ≤1 (ECOG).
9. Adequate respiratory functions: FEV1 > 1L.
10. Normal ECG values.
11. Adequate bone marrow function (hemoglobin ≥ 9 g/dL, leukocytes ≥ 3,000/mm^3, neutrophils ≥ 1,500/mm^3, platelets ≥ 100,000/mm^3). Subjects with plasma creatinine ≤ 1.6 mg/dL, transaminases ≤ 2.5 times the ULN, total bilirubin ≤ 1.25 times ULN, CPK ≤ 2.5 times ULN, alkaline phosphatase ≤ 2.5 times the ULN are acceptable. If alkaline phosphatase is > 2.5 times the ULN, then the alkaline phosphatase liver fraction and/or 5' nucleotidase and/or GGT must be ≤ ULN.
12. Men or women of child bearing potential should be using an effective method of contraception before entry into the study and throughout the same and for 6 months after ending the study. Women of childbearing potential must have a negative urine pregnancy test before study entry.
13. HBV and HCV serologies must be performed prior to inclusion. If HbsAg is positive it is recommended to reject the existence of replicative phase ($HbaAg^+$, DNA VHB^+) remaining at investigators' discretion the preventive treatment with lamivudine. If a potential subject is positive for anti-HCV antibodies, presence of the virus should be ruled out with a qualitative PCR, or the subject should NOT be included in the study (if a qualitative PCR cannot be performed then subject will not be able to enter the study).
14. Prior taxane therapy for any indication is accepted.
15. > Grade 3 (intense and diffuse) expression of SPARC by immunohistochemistry.

4.2.2 Exclusion criteria

1. Less than 4 weeks elapsed since prior exposure to chemotherapy.
2. Pre-existing neuropathy greater than Grade 1.
3. Other active invasive malignancy requiring ongoing therapy or expected to require systemic therapy within two years. However, localized squamous cell carcinoma of the skin, basal cell carcinoma of the skin, carcinoma in situ of the cervix or other malignancies requiring only locally ablative therapy, will not result in exclusion.
4. Concomitant anticancer therapy, immunotherapy or radiation therapy within prior 4 weeks.
5. Uncontrolled intercurrent illness including but not limited to ongoing or active infection requiring IV antibiotic, symptomatic congestive heart failure, unstable angina pectoris, cardiac arrhythmia, psychiatric illness or social situations that would limit compliance with study requirements.
6. Hb < 9 g/dL.
7. Women who are pregnant or breast-feeding.
8. Known hypersensitivity to protein bound paclitaxel.
9. Any other concurrent condition that in the investigators opinion would jeopardize compliance with the protocol.
10. Known positive test for infection by human immunodeficiency virus (HIV).
11. Subjects participating in another clinical trial or receiving any other investigational product.

4.3 Treatment discontinuation

Subjects who are discontinued from study drug for any reason are not discontinued from the study, and will continue to be followed until study discontinuation.

The following events are considered sufficient reasons for discontinuing a subject from the study drug:

- Unacceptable toxicity
- Adverse event(s)
- Disease progression
- Symptomatic deterioration (global deterioration of health status)
- Physician decision
- Withdrawal by subject
- Withdrawal by parent/guardian
- Death
- Lost to follow up
- Protocol violation
- Pregnancy
- Other (to be specified on CRF)

The reason for discontinuation must be recorded in the CRF and in the source documents. The decision to discontinue a subject from treatment remains the responsibility of the treating physician, which will not be delayed or refused by the Sponsor. However, prior to discontinuing a subject, the investigator may contact the Medical Monitor and forward appropriate supporting documents for review and discussion.

4.4 Subject discontinuation from the study

The following events are considered sufficient reasons for discontinuing a subject from the study:

- Withdrawal by subject
- Withdrawal by parent/guardian
- Death
- Lost to follow up
- Protocol violation
- Other

The reason for discontinuation must be recorded in the CRF and in the source documents.

4.5 Screening failures

A subject is considered to be a screening failure if the he or she signs the informed consent, but withdraws before study enrollment. All potential subjects who are screened in this study (including screening failures) will be listed on the subject screening log but will not be entered in the study database. Reasons for exclusion will be recorded for potential subjects who do not enter the study.

4.6 Study population definitions

The study population definitions are as follows:

- **Enrolled Population** – All subjects enrolled; ie, all subjects who are marked as enrolled in the clinical database regardless of whether they have received the study drug.

- **Safety Population** – All subjects who take at least one dose of study drug.

- **Efficacy Evaluable (EE) Population** – All treated subjects who meet eligibility criteria, complete at least two cycles of investigational product, and have baseline and at least one post-baseline efficacy assessment. Here efficacy assessment means radiological assessment of the tumor or tumor assessment by other appropriate means.

5. TREATMENT

5.1 Treatment administration

nab-paclitaxel (ABRAXANE) will be administered as follows:

- Age ≥ 21: 125 mg/m^2 days 1, 8 and 15 in cycles of 28 days
- Age ≥ 6 months and ≤ 20 years: 240 mg/m^2 (for patients weighing > 10 kg) and 11.5 mg/kg (for patients weighing ≤ 10 kg) on days 1, 8 and 15 in cycles of 28 days

Subjects in the DT cohort will receive a maximum of three cycles. Subjects in the DSRCT and ES cohort will receive unlimited cycles until disease progression, the subject begins a new anticancer treatment, withdrawal of parent/guardian/subject consent/assent, parent/guardian/subject refusal, physician decision, toxicity that cannot be managed by dose delay or dose reduction alone or the study ends for any reason.

nab-paclitaxel will be administered intravenously over approximately 30 minutes, without corticosteroid or antihistamine premedication.

Following administration, the intravenous line should be flushed with sodium chloride 9 mg/mL (0.9%) solution for injection to ensure administration of the complete dose, according to local practice.

5.2 Body surface area or weight changes

If the subject's BSA (or weight for subjects ≤ 10 kg) has changed by ≥ 10% from the last value used for dose calculations, then the subject's dose should be recalculated with the new value. Otherwise, dose adjustments should be based on actual body weight/BSA and follow institutional guidelines.

5.3 Dose modifications

Dose modifications for hematological toxicities are defined as follows:

**CYCLE DAY**	**NEUTROPHILS (Cells/mm^3)**	**or**	**PLATELETS (Cells/mm^3)**	**ABRAXANE DOSE**
Day 1	< 1,500	or	< 100,000	Delay up to recovery
Day 8	≥ 500 & < 1,000		≥ 50,000 & < 75,000	1st dose level reduction:<br>125 → 100 mg/m^2<br>240 → 192 mg/m^2
Day 8	< 500		< 50,000	No administration
Day 15	If dose on day 8 was given with no modification			
	≥ 500 & < 1,000	or	≥ 50,000 & <75,000	1st dose level reduction:<br>125 → 100 mg/m^2<br>240 → 192 mg/m^2
Day 15	< 500		< 50,000	No administration
	If dose on day 8 was already reduced			
Day 15	≥ 1000	or	≥ 75,000	Same dose of on day 8
Day 15	≥ 500 & < 1,000		≥ 50,000 & < 75,000	2nd dose level reduction:<br>100 → 80 mg/m^2<br>192 → 154 mg/m^2
Day 15	< 500		< 50,000	No administration
	If dose on day 8 was not administered			
Day 15	≥ 1,000	or	≥ 75,000	Same dose of on day 1 + GCSF
Day 15	≥ 500 & < 1,000		≥ 50,000 & < 75,000	1st dose level reduction:<br>125 → 100 mg/m^2<br>240 → 192 mg/m^2
Day 15	< 500		< 50,000	No administration

Table 1: Dose modifications for hematological toxicities

Preventive administration of GCSF (5 days) is recommended if neutrophil count is less than 500/mm^3 in previous blood test.

Dose modifications for other toxicities are defined as follows:

**Adverse Reaction**	**ABRAXANE DOSE**
Febrile neutropenia	One dose level reduction for the following doses*
G3/G4 peripheral neuropathy	Temporarily interruption up to ≤ G1 and reduce one dose level for the following administrations
G2/G3 skin toxicity	Reduce one dose level for the following administrations. If toxicity persists stop the treatment
G3/G4 diarrhea or mucositis	Interruption of the treatment up to ≤ G1 and reduce one dose level for the following administrations
Other G3/G4 toxicities	Interruption of the treatment up to ≤ G1 and reduce one dose level for the following administrations

(*) Preventive GCSF for the following administrations

Table 2: Dose modifications for other toxicities

Subjects with mild hepatic insufficiency (total bilirubin > 1 to ≤ 1.5 x ULN and AST or ALT ≤ 10 x ULN) do not require dose adjustments.

The dose reductions for other toxicities are defined as follows:

- First dose reduction:
  125 → 100 mg/m^2
  240 → 192 mg/m^2

- Second dose reduction:
  100 → 80 mg/m^2
  192 → 154 mg/m^2

- If additional dose reduction is required, treatment must be stopped.

5.4 Treatment delays

Study drug administration should occur within a ± 2 day administrative window of the scheduled day of dosing. The study drug may be delayed for other reasons based on the guideline below. If Day 8 or Day 15 treatment is delayed ≤ 4 days, the treatment may be administered and the 28 days cycle continues per protocol. If the investigator suspects a drug related toxicity, an unscheduled visit with additional laboratory assessments may be performed. When treatment (either Day 1, 8, or 15) is consecutively delayed for two or more cycles, dose reduction should be considered. Subjects experiencing study drug-related toxicities that require a delay in scheduled dosing of study drug for > 21 days will be discontinued from further participation in this study (except if day 22 falls on a weekend or holiday, in which case study drug may be administered the next business day).

Day 1 Treatment Delay
If the treatment to be given on Day 1 is delayed, the 28-day cycle will not be considered to start until the day the study drug is actually administered to the subject.

Day 8 Treatment Delay
If the treatment to be given on Day 8 is held for ≤ 4 days, the treatment may be administered and the 28-day cycle continues per protocol. If Day 8 treatment is delayed > 4 days, the treatment will be considered as missed and will not be made up.

Day 15 Treatment Delay
If the treatment to be given on Day 15 is held for ≤ 4 days, the treatment may be administered and the 28-day cycle continues per protocol. If Day 15 treatment is delayed > 4 days, that week becomes the week of

rest. The next dose (if counts and chemistries permit) becomes Day 1 of a new cycle, and the subject is considered to have had a 21-day cycle. The subsequent cycle resumes the scheduled 28-day cycle.

5.5 Missed treatment

A missed Day 8 or Day 15 treatment is defined as study drug not administered within ≤ 4 days of the expected dose day and should be entered in the CRF as a missed dose. If the investigator suspects drug-related toxicity, an unscheduled visit with additional laboratory tests may be performed.

5.6 Treatment overdose

On a per dose basis, an overdose is defined as 10% over the protocol-specified dose of study drug assigned to a given subject, regardless of any associated adverse events or sequelae. On a schedule or frequency basis, an overdose is defined as anything more frequent than the protocol required schedule or frequency. On an infusion rate basis, an overdose is defined as any rate faster than the protocol-specified rate. Complete data about drug administration, including any overdose, regardless of whether the overdose was accidental or intentional, should be reported in the case report form. See section for the reporting of adverse events associated with overdose.

5.7 Concomitant medications and procedures

Over the course of this trial, additional medications may be required to manage aspects of the disease state of the subjects, including side effects from trial treatments or disease progression.

Supportive care, including but not limited to anti-emetic medications, may be administered at the discretion of the investigator.

All concomitant treatments, including blood and blood products, must be reported on the CRF.

For information regarding other drugs that may interact with nab-paclitaxel and affect its metabolism, pharmacokinetics, or excretion, please see the nab-paclitaxel (ABRAXANE) local package insert.

5.7.1 Permitted concomitant medications and procedures

Erythropoietin may be administered at the discretion of the investigator, consistent with institutional guidelines. Granulocyte colony-stimulating factors may be given according to institutional guidelines for the treatment of neutropenic fever or infections associated with neutropenia and for the prevention of febrile neutropenia in subjects with an ANC < 500 cells/µL.

5.7.2 Prohibited concomitant medications and procedures

Granulocyte colony-stimulating factors are allowed during the study treatment. Radiotherapy is not allowed during the study, except for palliative purposes for non-target lesions. Surgical interventions as anticancer therapy during Cycle 1 will require the subject to discontinue from study treatment. Minor surgical interventions in subsequent cycles will be assessed on a case by case basis after discussion with the Clinical Research Physician.

Administration of other chemotherapy, immunotherapy, antitumor hormonal therapy, investigational therapy, or other anticancer therapy during the study is not allowed.

Administration of Coumadin or coumarin derivatives is not allowed during this study; low-molecular weight heparin should be used instead.

The potential drug-drug interaction precautions contained in the *nab*-paclitaxel prescribing information will be applied to this study (refer to the local prescribing information). Specifically, the metabolism of paclitaxel is catalyzed by cytochrome P450 isozymes CYP2C8 and CYP3A4.

Strong inducers of CYP3A4 and CYP2C8 are prohibited for use from the first dose of study drug until permanent discontinuation. Such medications include but are not limited to:

- Strong inducers of CYP3A4: avasimibe, carbamazepine, phenytoin, rifampin, and St. John's wort.

Strong inhibitors of CYP3A4 and CYP2C8 should be avoided whenever possible from the first dose of study drug until permanent discontinuation. If possible, subjects should be switched to other medications for the comorbidity prior to starting the study drug. Such medications include but are not limited to:

- Strong inhibitors of CYP2C8: gemfibrozil
- Strong inhibitors of CYP3A4: boceprevir, clarithromycin, conivaptan, grapefruit juice, itraconazole, ketoconazole, lopinavir/ritonavir, mibefradil, nefazodone, posaconazole, telaprevir, telithromycin, and voriconazole.

Caution is recommended when administering *nab*-paclitaxel concomitantly with any substrates or inhibitors of the cytochrome P450 isozymes CYP2C8 and CYP3A4. Similarly, drugs, herbal preparations, and/or dietary supplements known to influence the expression of CYP3A (eg, garlic supplements) and/or CYP2C8 should be used with caution (see www.druginteractions.com for a regularly updated list of drug interactions with cytochrome P450 isozymes).

Please check the prescribing information of the concomitant medication for full information on the CYP interaction potential.

Subjects and parent(s)/guardian(s) must be made aware that the following medications are not allowed to be taken concomitantly with nab-paclitaxel: ritonavir, saquinavir, indinavir, nelfinavir, or investigational drug(s) other than described as a treatment regimen in this study.

6. DRUG MANAGEMENT

6.1 Supply and distribution of study drug

Nab-paclitaxel (ABRAXANE) is a drug developed and marketed by Celgene. In the GEIS-39 trial, nab-paclitaxel will be supplied by Celgene through its own distribution channels.

6.2 Packaging and labeling

The label(s) for the study drug will include Sponsor name, address, and telephone number, the protocol number, drug name, dosage form and strength (where applicable), amount of drug per container, lot number, expiry date (where applicable), medication identification/kit number, dosing instructions, storage conditions, and required caution statements and/or regulatory statements as applicable. Additional information may be included on the label as applicable per local regulations.

6.3 Accountability and disposal

The Sponsor (or designee) will review with the investigator and relevant site personnel the process for study drug return, disposal, and/or destruction including responsibilities for the site vs Sponsor (or designee).

Partially used and completely used vials should be destroyed according to local guidelines, and disposition should be recorded on the Investigational Drug Accountability Record Form.

The investigator, or designee, shall record the dispensing of study drug to subjects in the study drug accountability record. The study drug record will be made available to the Sponsor, or other authorized Sponsor-designated monitoring personnel, for the purpose of accounting for the study drug supply. Inspections of the study drug supply for inventory purposes and assurance of proper storage will be conducted as necessary.

Any significant discrepancy will be recorded and reported to the Sponsor or its designee and a plan for resolution will be documented.

Investigational product will not be loaned or dispensed by the investigator to another investigator or site.

6.4 Drug compliance

Accurate recording of all study drug administration will be made in the appropriate section of the subject's CRF and source documents. The investigator or designee is responsible for accounting for all study-specific drug both administered or in their custody during the course of the study.

6.5 Local pharmacy responsibilities

The responsibilities of the designated responsible pharmacist(s) at each participating center include, but are not limited to, ensuring that:

- Study drug is handled and stored safely and according to product specific requirements;
- The study drug is dispensed only to trial subjects and in accordance with the protocol;
- There is a sufficient supply of study drug for subjects' continued treatment, and in a timely manner arrange for re-supply of stock;
- Study drug expiry dates are monitored and drug is used in order of expiry date (i.e. earliest expiry first);
- Unused study drug is destroyed locally in accordance with local protocol;
- Study drug receipt, accountability and destruction records are maintained.

7. STUDY PROCEDURES AND ASSESSMENTS

7.1 Calendar of activities

Table 3: Procedures and assessments (Desmoid tumor)

Protocol section reference	Study stages >>	BEFORE TREATMENT	TREATMENT					END OF TREATMENT[1] (+/- 7 days)	FOLLOW-UP (+/- 7 days)
		Screening (≤ 28 days of start of treatment)	Every cycle (up to 3 cycles)						
			Week 1 (Day 1)	Week 2 (Day 8)	Week 3 (Day 15)				
**7.2**	**Subject information and consent**								
	Subject information	X (prior to screening)							
	Informed consent	X (prior to screening)							
**7.3**	**Eligibility and enrollment procedures**								
	Screening number assignment	X							
	Central pathology review	X							
	Eligibility confirmation	X							
	Subject enrollment	X							
**7.4**	**Clinical assessments**								
	Demographic data and medical history	X							
	ECOG	X	X					X	
**7.5**	**Laboratory assessments (+/- 3 days)**								
	Biochemistry[2]	X	X[3]	X	X			X	
	Hematology[4]	X	X[5]	X	X			X	
	Coagulation tests[6]	X							
	Thyroid function test	X							
	Pregnancy test	X							
	HBV/HCV serology	X							
**7.6**	**Safety assessments**								
	Physical examination	X	X					X	X
	Vital signs[7]	X	X	X	X			X	
	Adverse events[8]	X	X	X	X			X	X
	Concomitant medication	X	X	X	X			X	
	ECG[9]	X							
	LVEF	X						X	
**7.7**	**Efficacy assessments**								
	MRI (including diffusion and perfusion)	X						X[10]	X
	Central radiology review							X	
**7.8**	**Pain assessments[11]**								
	Brief Pain Inventory (BPI)	X	X	X	X			X	X
	Analgesic Quantification Algorithm (AQA)	X	X	X	X			X	X
**7.9**	**Treatment management**								
	Treatment administration[12]		X	X	X				
	Treatment compliance				X				
**7.10**	**Biological sample collection**								
	Tumor block collection	X							
	Blood sample collection	X						X	
**7.11**	**Survival**								
	Follow-up visits								X[13]

1. End of treatment for desmoid tumor cohort is on day 84 (after 3 cycles of 28 days).
2. Biochemistry: ALT, AST, bilirubin, creatinine, CPK, GGT, ALP, glucose, albumin, total protein, LDH, urea, sodium, potassium, calcium, magnesium, inorganic phosphate.
3. Biochemistry must be performed on day 1 if not done within one week prior to treatment initiation.
4. Hematology: hemoglobin , WBC, RBC, lymphocytes, platelets, neutrophils, hematocrit.
5. Hematology must be performed on day 1 if not done within one week prior to treatment initiation.
6. Coagulation (partial thromboplastin time, fibrinogen and INR) may be repeated if clinically indicated.
7. Vital signs include: blood pressure, heart rate and temperature.
8. AEs must be collected from the signature of the informed consent, during the entire treatment and up to 28 days after last administration of study drug.
9. ECG may be repeated if clinically indicated.
10. Tumor assessment should be performed if end of treatment was not due to radiological progression, or if the last radiological imaging was performed more than 2 months ago.
11. BPI and AQA only for desmoid tumor cohort. At baseline, days 1, 8, and 15, at the end of treatment, and then every 3 months for 3 years.
12. Nab-paclitaxel will be administered on days 1, 8, and 15 of each cycle.
13. Every 3 months during the first year and every 6 months for 2 additional years (maximum follow-up within the study).

Table 4: Procedures and assessments (Desmoplastic small round cell tumor and Ewing's sarcoma)

**Protocol section reference**	**Study stages >>**	**BEFORE TREATMENT**	**TREATMENT**					**END OF TREATMENT[1] (+/- 7 days)**	**FOLLOW-UP (+/- 7 days)**
		**Screening (≤ 28 days of start of treatment)**	Every cycle (until progression)				Every 8 weeks		
			**Week 1 (Day 1)**	**Week 2 (Day 8)**	**Week 3 (Day 15)**				
**7.2**	**Subject information and consent**								
	Subject information	X (prior to screening)							
	Informed consent	X (prior to screening)							
**7.3**	**Eligibility and enrollment procedures**								
	Screening number assignment	X							
	Central pathology review	X							
	Eligibility confirmation	X							
	Subject enrollment	X							
**7.4**	**Clinical assessments**								
	Demographic data and medical history	X							
	ECOG	X	X					X	
**7.5**	**Laboratory assessments (+/- 3 days)**								
	Biochemistry[2]	X	X[3]	X	X			X	
	Hematology[4]	X	X[5]	X	X			X	
	Coagulation tests[6]	X							
	Thyroid function test	X							
	Pregnancy test	X							
	HBV/HCV serology	X							
**7.6**	**Safety assessments**								
	Physical examination	X	X					X	X
	Vital signs[7]	X	X	X	X			X	
	Adverse events[8]	X	X	X	X		X	X	X
	Concomitant medication	X	X	X	X		X	X	
	ECG[9]	X							
	LVEF	X						X	
**7.7**	**Efficacy assessments**								
	MRI/Thoracic-abdominal CT scan	X					X	X[10]	X
	Central radiology review							X	
**7.9**	**Treatment management**								
	Treatment administration[11]		X	X	X				
	Treatment compliance				X				
**7.10**	**Biological sample collection**								
	Tumor block collection	X						X	
**7.11**	**Survival**								
	Follow-up visits								X[12]

1. DSRCT and ES cohort continues until progression.
2. Biochemistry: ALT, AST, bilirubin, creatinine, CPK, GGT, ALP, glucose, albumin, total protein, LDH, urea, sodium, potassium, calcium, magnesium, inorganic phosphate.
3. Biochemistry must be performed on day 1 if not done within one week prior to treatment initiation.
4. Hematology: hemoglobin, WBC, RBC, lymphocytes, platelets, neutrophils, hematocrit.
5. Hematology must be performed on day 1 if not done within one week prior to treatment initiation.
6. Coagulation (partial thromboplastin time, fibrinogen and INR) may be repeated if clinically indicated.
7. Vital signs include: blood pressure, heart rate and temperature.
8. .AEs must be collected from the signature of the informed consent, during the entire treatment and up to 28 days after last administration of study drug.
9. ECG may be repeated if clinically indicated.
10. Tumor assessment should be performed if end of treatment was not due to radiological progression, or if the last radiological imaging was performed more than 2 months ago.
11. Nab-paclitaxel will be administered on days 1, 8, and 15 of each cycle.
12. Every 3 months during the first year and every 6 months for 2 additional years (maximum follow-up within the study).

7.2 Subject information and consent

All candidate subjects will receive a subject information sheet describing, in simple language, the goals, scope, procedures and relevant implications of the clinical trial. The information sheet will integrate an informed consent form to be signed by the subject (parent/tutor in the case of subjects under 18 years), which is indispensable for study participation. Written informed consent must be given by each subject (or parent/tutor) before screening process initiation (prior to undergoing protocol-specific evaluations and prior to receiving treatment). The subject (parent/tutor) may sign two separate informed consent forms: one for the main study and another one for the biological samples. Subjects must be willing to comply with treatment and follow-up. Procedures conducted as part of the subject's routine clinical management (e.g. blood count, imaging studies such as CT/MRI scans) and obtained prior to signing of informed consent may be utilized for screening/baseline purposes provided these procedures are conducted as specified in the protocol.

7.3 Eligibility and enrollment procedures

Screening number assignment

Once the required informed consent forms have been signed, a unique screening number will be assigned to each subject. Each site will receive inside the Site Investigator File a screening log form with a list of predetermined screening numbers to be assigned. This document should be always at site under the research team custody. This screening number will identify subjects throughout the procedures needed to confirm their suitability for the trial protocol (clinical laboratory tests, imaging tests, central pathology review, etc.).

Central pathology review

After screening number assignment, at least one pre-treatment representative formaline-fixed paraffin-embedded tumor block will be collected for central pathology review. First diagnosis sample or another more recent available sample obtained during the routine care previous to study entry will be acceptable. Subjects cannot be included in the study unless a tumor block is available. Tumor biopsy at the start of the study is not compulsory but recommended if clinically acceptable. The tumor sample is to be shipped by courier to the central pathology review laboratory along with (1) the trial-specific pathology review form and (2) the center's anonymized pathology report. Diagnosis confirmation from the central pathology reviewer will be available in approximately one week. The central pathology review is a compulsory requirement for trial entry in all cases without exception. The study treatment cannot be initiated unless the diagnosis has been confirmed by this means.

An additional document will be provided in the Investigator Site File with detailed tumor collection and shipment procedures.

The main contact point to coordinate tumor block shipments will be:

Sofpromed Investigación Clínica, SLU
Telephone: +34 648 414 261
E-mail: ensayos@sofpromed.com

Eligibility confirmation

The Principal Investigator of each site will be responsible for confirming that the subject to be recruited meets all inclusion criteria and does not meet the exclusion criteria. Subject enrollment will be carried out after eligibility confirmation.

Subject enrollment

Following completion of all screening procedures and eligibility confirmation, subjects will then be enrolled to begin study treatment.

The procedure to enroll a subject is described as follows:

- Complete and sign the subject enrollment form (the form must be signed by an authorized investigator).

- Send (via email or fax) the enrollment form directly to the study CRO in Spain:

Sofpromed Investigación Clínica, SLU
Fax: +34 971 570 222
E-mail: ensayos@sofpromed.com

- The study CRO will carry out the subject enrollment process and will then send back (via email) to the center the subject enrollment confirmation notification. This notification will contain a unique subject number that will identify the subject at all times during the trial.

Additional documentation will be provided in the Investigator Site File with detailed subject enrollment guidelines.

7.4 Clinical assessments

Demographic data and medical history

Demographic information, past or current medical conditions and treatments, current medications, medications taken within 30 days of inclusion, date of diagnosis, prior cancer therapy and surgery, pathological confirmation of malignancy, and staging of the tumor should be recorded. Any pre-existing toxicity (e.g. Grade 1 fatigue) should be documented at this time.

ECOG

ECOG performance status will be assessed at screening: 14 days and 7 days prior to the start of study drug administration. Thereafter, at day 1 of each cycle and at the end of the treatment. If subjects discontinue study treatment without disease progression (e.g. withdrawal of study treatment due to unacceptable toxicity), continue the assessments of ECOG performance status in accordance with the disease assessments until subjects experience disease progression.

7.5 Laboratory assessments

Laboratory assessments should be performed as indicated in the trial calendar of activities. These assessments may be carried out within 3 days before the actual visit to allow flexibility in scheduling. Assessments may be performed more frequently if clinically indicated. Correction of electrolytes (most importantly, potassium, magnesium and calcium) to within normal ranges should take place prior to study entry and during study conduct as clinically indicated.

All laboratory tests with values that become abnormal and clinically significant while the subject is participating in the study or within 28 days after the last dose of study drug should be repeated until the values return to normal or baseline. See Section 5 of this protocol for guidance on subject follow-up and dose management in response to specific laboratory abnormalities.

Results for all unscheduled clinical laboratory assessments (e.g. hematology, TSH/T_4, coagulation parameters) should be recorded on appropriate e-CRF forms.

Table 5: Clinical laboratory assessments

**Clinical Chemistry**	
Renal function	Urea, Creatinine[a]
Liver function test (LFT) Panel	Albumin, Alkaline phosphatase, Alanine aminotransferase (ALT), Aspartate aminotransferase (AST), Gamma-glutamyltransferase (GGT), and Bilirubin (total)
Electrolytes and others	Calcium, Potassium, Sodium, Magnesium, Inorganic phosphate, Glucose, Lactate dehydrogenase (LDH), Creatine phosphokinase (CPK), and Total protein
**Hematology**	Hematocrit, Hemoglobin, White Blood Cell Count, Red Blood Cell Count, Neutrophils, Lymphocytes, and Platelets
**Coagulation Tests**	Activated partial thromboplastin (aPTT), International Normalization Ratio (INR), and Fibrinogen
**Thyroid Function Test**	TSH
**Pregnancy Test**	The study drug must not be administered to pregnant or breast feeding women. For women in fertile age, a negative serum or urine pregnancy test must be confirmed (minimum sensitivity 25 mIU/L or equivalent units of beta human chorionic gonadatropin [β-HCG]) within 72 hours prior to enrollment. Thereafter, the pregnancy test only needs to be repeated if clinically indicated or as required by local regulations.<br><br>A female is eligible to enter and participate in this study if she is of:<br><br>Non-childbearing potential (i.e., physiologically incapable of becoming pregnant), including any female who has had a hysterectomy, a bilateral oophorectomy (ovariectomy), a bilateral tubal ligation, or is post-menopausal.<br><br>Female subjects not using hormone replacement therapy (HRT) must have experienced total cessation of menses for ≥ 1 year and be greater than 45 years in age, OR, in questionable cases, have a follicle stimulating hormone (FSH) value > 40 mIU/mL and an estradiol value < 40 pg/mL (<140 pmol/L).<br><br>Female subjects using HRT must have experienced total cessation of menses for ≥ 1 year and be greater than 45 years of age OR have had documented evidence of menopause based on FSH and estradiol concentrations prior to initiation of HRT.<br><br>All subjects (male and female) must agree to use adequate contraception methods during study treatment and up to 6 months after the last dose. The acceptable contraceptive methods, when used consistently and in accordance with both the product label and the instructions of the physician, are as follows:<br><br>• Oral contraceptive, either combined or progestogen alone<br>• Injectable progestogen<br>• Implants of levonorgestrel<br>• Estrogenic vaginal ring<br>• Percutaneous contraceptive patches<br>• Intrauterine device (IUD) or intrauterine system (IUS) with a documented failure rate of less than 1% per year<br>• Male partner sterilization (vasectomy with documentation of

	azoospermia) prior to the female subject's entry into the study, and this male is the sole partner for that subject<br>• Double barrier method: condom and an occlusive cap (diaphragm or cervical/vault caps) with a vaginal spermicidal agent (foam/gel/film/cream/suppository)<br><br>Female subjects who are lactating must discontinue nursing prior to the first dose of study drug and must refrain from nursing throughout the treatment period and for 90 days following the last dose of study drug.
**HBV/HCV Serology**	HBV and HCV serologies must be performed prior to inclusion. If HbsAg is positive it is recommended to reject the existence of replicative phase ($HbaAg^{+}$, DNA VHB^{+}) remaining at investigators' discretion the preventive treatment with lamivudine. If a potential subject is positive for anti-HCV antibodies, presence of the virus should be ruled out with a qualitative PCR, or the subject should NOT be included in the study (if a qualitative PCR cannot be performed then subject will not be able to enter the study).

a. Estimated creatinine clearance should be calculated using the Cockroft and Gault method (Appendix B). Alternatively, creatinine clearance can be measured directly by 24-hour urine collection.

7.6 Safety assessments

Any subject included in the study receiving at least a single dose of study medication will be evaluable for the toxicity analysis. Safety profile will be characterized by treatment-emergent adverse events, vital signs and laboratory abnormalities. Assessment of adverse events will include type, incidence, severity (graded by the National Cancer Institute [NCI] Common Terminology Criteria for Adverse Events [CTCAE], Version 4.0), timing, seriousness, and relatedness; and laboratory abnormalities. Baseline tumor-related signs and symptoms will be recorded as adverse events during the trial if they worsen in severity or increase in frequency. In each study visit all adverse events will be registered.

Physical examination and vital signs

Pre-treatment (screening): Evaluation by body system, height, weight, body surface area (BSA) and measurement of vital signs (blood pressure and body temperature).

During treatment and follow-up: Evaluation by body system, weight, body surface area (BSA) and verification of subject blood pressure measurements, at day 1 of each cycle, at the end of treatment and at follow-up visits.

Adverse events

The CTC-AE version 4.0 will be used to evaluate the clinical safety of the treatment in this study. Subjects will be assessed for AEs at each clinical visit and as necessary throughout the study and up to 28 days after last administration of study drug.

Concomitant medication

The use of any natural/herbal products or others “folk remedies” should be discouraged but use of these products, as well as use of vitamins or nutritional supplements and all others concomitant medications must be recorded in the electronic case report form (e-CRF). Any medications, with the exceptions noted in section 5 of this protocol, which are considered necessary for subject´s welfare, and which it is believed will not interfere with the study medication, may be given at the discretion of the investigator, registering the medication, doses, dates and reasons for administration in the e-CRF.

ECG

A baseline ECG is to be obtained within 14 days prior to inclusion. ECG may be repeated afterwards if clinically indicated.

LVEF

MUGA or echocardiogram will be performed at baseline and at the end of treatment. A MUGA scan is the preferred method for left ventricular ejection fraction (LVEF) measurement. If a MUGA scan cannot be performed, an echocardiogram should be done. The same type of LVEF assessment must be performed at baseline and at all subsequent points in the study. A MUGA scan should be performed sooner if a subject develops signs and symptoms of congestive heart failure (e.g. shortness of breath during mild exertion or when lying down, feeling very tired, cough -especially at night-, swelling of the feet and/or ankles).

7.7 Efficacy assessments

MRI / Thoracic-abdominal CT scan

Imaging tests will be performed with MRI or thoracic-abdominal CT scan (contrast enhanced, biphasic) as appropriate. **Desmoid tumors will only be assessed with MRI (diffusion and perfusion –dynamic study– imaging is mandatory).** Imaging methods will be employed consistently during the course of each subject's evaluation along the study. Disease should be captured and target/non-target lesions identified at baseline. All the baseline disease assessments should be completed within 28 days prior to the date of inclusion in the trial. Subsequently, imaging studies required to investigate known disease should be done **at the end of the third cycle in the case of DT and every 8 weeks in the case of DSRCT and ES** (until tumor progression is documented in subjects with complete response, partial response or stable disease). Subjects who have not progressed but discontinued treatment due to toxicity or other reasons (unrelated to tumor progression) will still be re-evaluated every 3 months (in the case of DT) and every 2 months (in the case of DSRCT and ES), unless they have started a new anti-cancer therapy.

	**Choi**	**RECIST**
Complete Response (CR)	Disappearance of all lesions.<br>No new lesions.	Disappearance of all lesions
Partial Response (PR)	Decrease in size ≥ 10% of SLD or decrease of density ≥ 15% UH.<br>No new lesions.<br>Absence of progression of non-measurable disease.	Decrease of 30% of the sum of the diameters of target lesions
Stable Disease (SD)	Does not fulfill CR, PR or DP.<br>There is no symptomatic deterioration attributable to tumor progression.	Between PR and DP
Disease Progression (DP)	A tumor increase ≥ 10% of SLD and without PR criteria for the radiological density in the CT scan.<br>New lesions. New intra-tumor nodules or increase of existing nodules or increase of tissue part of a hypodense lesion	Increase of 20% of the total diameter or appearance of new lesions

* SLD: Sum of longest diameter (based on RECIST 1.1).

Table 6: Choi/RECIST criteria for response evaluation

To perform the radiological studies, the following protocols will have to be applied:

Magnetic Resonance (MRI) [diffusion and perfusion –dynamic study– mandatory for DT]

- Morphological pre-contrast examination: Always Axial T1 SE, and T2 FSE (with or without fat pre-saturation) or STIR, other sequences depending of local practice.

- Diffusion study: Axial b50, b400, b800, b1000.
- Dynamic examination:
  Before contrast injection: GE 3D TR less than 1 min Variable Flip angle (5, 10, 15, 20, 25, 28).
  During contrast injection: GE 3D TR less than 1 min (if possible 4 s) for 5 min.
- Morphological post-contrast examination: Coronal or sagittal T1W sequence with fat sat; Axial T1 SE without fat sat.
- Tumor enhancement after contrast will be determined by measuring MR signal by drawing a region of interest around the margin of the entire tumor in the subtracted series from Axial-T1-SE after CE minus Axial-T1-SE before contrast (parameters and position must be identical in both sequences). Two-dimensional regions of interest of the entire lesion will be drawn, and all axial sections encompassing the lesion will be included. Software will calculate semi-quantitatively the mean tumor signal as the average of all the pixels enclosed in the volume of interest.

Computed Tomography (CT Scan)

- Pre-contrast examination.
- Contrast examination: To be performed using 120 mL of a conventional iodinated contrast agent, administered intravenously by an automated injector at a rate of 4 mL/s; the contrast examination will be done in: arterial phase (delay: bolus tracking), portal phase (60 s), delayed phase (6 min).
- Coronal mpr in delayed phase.
- Tumor density will be determined by measuring CT attenuation coefficient in Hounsfield Unit (HU) by drawing a region of interest around the margin of the entire tumor, using section thickness of 5 mm in the portal and delayed phase. Two-dimensional regions of interest of the entire lesion will be drawn, and all axial sections encompassing the lesion will be included. Software will calculate semiquantitatively the mean tumor attenuation in HU defined as the average of all the pixels enclosed in the volume of interest.
- It is strongly recommended to use CT scan when feasible, instead of MRI.

Central imaging review

All imaging tests will be centrally reviewed by using a web-based platform. The person in charge of performing these reviews will be a radiologist designated by the Sponsor.

It is strongly recommended to upload the baseline MRI/CT scan before subject enrollment. If this is not feasible, it should be done as soon as possible. All tumor imaging tests performed during the clinical trial should be uploaded to the imaging platform as soon as possible. All scans generated should be exportable in electronic format (DICOM) to enable secure and rapid electronic transmission to the designated central imaging laboratory.

All scans will be anonymized before upload and identified only with the subject's unique trial number. A central radiology review report will be generated as per specific form.

For any issue regarding central imaging reviews, please contact:

Sofpromed Investigación Clínica, SLU
Tel: +34 648 414 261
E-mail: **ensayos@sofpromed.com**

An additional document will be provided in the Investigator Site File with detailed central imaging upload information.

7.8 Pain assessments

Please refer to Appendix A for pain assessments, including Brief Pain Inventory (BPI) and Analgesic Quantification Algorithm (AQA).

7.9 Treatment management

- Administration: Nab-paclitaxel will be administered to the subjects in each hospital facilities.
- Compliance: Local pharmacies must keep records of the nab-paclitaxel vials administered to subjects. This information is to be entered in the e-CRF.

7.10 Biological sample collection

Biological samples will be collected as specified in section 11.3 of this protocol.

7.11 Survival

All subjects must be followed until death, if possible. The date and cause of death must be evaluated and documented in the e-CRF. In the follow-up visits subject status will be assessed (alive, dead, lost to follow up), as well as any new anti-cancer treatments.

8. PHARMACOVIGILANCE

8.1 Monitoring, Recording and reporting of adverse events

An adverse event (AE) is any noxious, unintended, or untoward medical occurrence that may appear or worsen in a subject during the course of a study. It may be a new intercurrent illness, a worsening concomitant illness, an injury, or any concomitant impairment of the subject's health, including laboratory test values (as specified by the criteria below), regardless of etiology. Any worsening (i.e., any clinically significant adverse change in the frequency or intensity of a preexisting condition) should be considered an AE. A diagnosis or syndrome, rather than the individual signs or symptoms of the diagnosis or syndrome, should be recorded on the AE page of the CRF.

Clinically significant signs and symptoms associated with disease progression are expected to be reported as AEs; however, study indication progressive disease (PD) that is asymptomatic or solely documented radiographically per RECIST 1.1/MIBG does not require reporting as an AE.

Abuse, withdrawal and overdose (accidental or intentional) to an investigational product should be reported as an AE. Any sequela of an accidental or intentional overdose of an investigational product should be reported as an AE or serious adverse events (SAE). In the event of overdose, the subject should be monitored as appropriate and should receive supportive measures as necessary. Actual treatment should depend on the severity of the clinical situation and the judgment and experience of the treating physician.

All subjects will be monitored for AEs during the study. Assessments may include monitoring of any or all of the following parameters: the subject's clinical symptoms, laboratory, pathological, radiological or surgical findings, physical examination findings, or other appropriate tests and procedures.

All AEs (including serious adverse events (SAEs)) will be recorded by the investigator from the time the parent/guardian/subject signs the informed consent/assent document (if applicable) to 28 days after the last dose of study drug and during the end of treatment visit. Serious adverse events made known to the investigator at any time thereafter that are suspected of being related to study drug will also be recorded.

AEs and SAEs will be recorded on the AE page of the CRF and in the subject's source documents.

8.2 Evaluation of adverse events

A qualified investigator will evaluate all adverse events as to:

8.2.1 Seriousness

A SAE is any AE occurring at any dose that:

- Results in death
- Is life-threatening (i.e., in the opinion of the investigator, the subject is at immediate risk of death from the AE)
- Requires inpatient hospitalization or prolongation of existing hospitalization (hospitalization is defined as a subject admission, regardless of length of stay)
- Results in persistent or significant disability/incapacity (a substantial disruption of the subject's ability to conduct normal life functions)
- Is a congenital anomaly/birth defect
- Constitutes an important medical event.

Important medical events are defined as those occurrences that may not be immediately life threatening or result in death, hospitalization, or disability, but may jeopardize the subject or require medical or surgical intervention to prevent one of the other outcomes listed above.

Medical and scientific judgment should be exercised in deciding whether such an AE should be considered serious.

Events **not considered** to be SAEs are hospitalizations for:

- A standard procedure for protocol therapy administration. However, hospitalization or prolonged hospitalization for a complication of therapy administration will be reported as an SAE.
- Routine treatment or monitoring of the studied indication not associated with any deterioration in condition.
- The administration of blood or platelet transfusion as routine treatment of studied indication. However, hospitalization or prolonged hospitalization for a complication of such transfusion remains a reportable SAE.
- A procedure for protocol/disease-related investigations (e.g., surgery, scans, endoscopy, sampling for laboratory tests, bone marrow sampling). However, hospitalization or prolonged hospitalization for a complication of such procedures remains a reportable SAE.
- Hospitalization or prolongation of hospitalization for technical, practical, or social reasons, in absence of an AE.
- A procedure that is planned (i.e., planned prior to starting of treatment on study); must be documented in the source document and the CRF. Hospitalization or prolonged hospitalization for a complication remains a reportable SAE.
- An elective treatment of a pre-existing condition unrelated to the studied indication.
- Emergency outpatient treatment or observation that does not result in admission, unless fulfilling other seriousness criteria above.

If an AE is considered serious, both the AE form/screen of the CRF and the SAE Report Form must be completed.

For each SAE, the investigator will provide information on severity, start and stop dates, relationship to study drug, action taken regarding study drug, and outcome.

8.2.2 Severity / Intensity

For both AEs and SAEs, the investigator must assess the severity / intensity of the event.

The severity / intensity of AEs will be graded based upon the subject's symptoms according to the current active minor version of the NCI CTCAE, Version 4.0.

AEs that are not defined in the NCI CTCAE should be evaluated for severity / intensity according to the following scale:

- Grade 1 = Mild – transient or mild discomfort; no limitation in activity; no medical intervention/therapy required
- Grade 2 = Moderate – mild to moderate limitation in activity, some assistance may be needed; no or minimal medical intervention/therapy required
- Grade 3 = Severe – marked limitation in activity, some assistance usually required; medical intervention/therapy required, hospitalization is possible
- Grade 4 = Life threatening – extreme limitation in activity, significant assistance required; significant medical intervention/therapy required, hospitalization or hospice care probable
- Grade 5 = Death - the event results in death

The term "severe" is often used to describe the intensity of a specific event (as in mild, moderate or severe myocardial infarction); the event itself, however, may be of relatively minor medical significance (such as severe headache). This criterion is *not* the same as "serious" which is based on subject/event *outcome* or *action* criteria associated with events that pose a threat to a subject's life or functioning.

Seriousness, not severity, serves as a guide for defining regulatory obligations.

8.2.3 Causality

The investigator must determine the relationship between the administration of study drug and the occurrence of an AE/SAE as Not Suspected or Suspected as defined below:

- Not suspected: Means a causal relationship of the adverse event to study drug administration is **unlikely or remote**, or other medications, therapeutic interventions, or underlying conditions provide a sufficient explanation for the observed event.

- Suspected: Means there is a **reasonable possibility** that the administration of study drug caused the adverse event. 'Reasonable possibility' means there is evidence to suggest a causal relationship between the study drug and the adverse event.

Causality should be assessed and provided for every AE/SAE based on currently available information. Causality is to be reassessed and provided as additional information becomes available.

If an event is assessed as suspected of being related to an ancillary treatment, please provide the name of the manufacturer when reporting the event.

8.2.4 Duration

For both AEs and SAEs, the investigator will provide a record of the start and stop dates of the event.

8.2.5 Action Taken

The investigator will report the action taken with the study drug as a result of an AE or SAE, as applicable (e.g., discontinuation or reduction of study drug, as appropriate) and report if concomitant and/or additional treatments were given for the event.

8.2.6 Outcome

The investigator will report the outcome for both AEs and SAEs. All SAEs that have not resolved upon discontinuation of the subject's participation in the study must be followed until recovered, recovered with sequelae, not recovered, or death (due to the SAE).

8.3 Abnormal laboratory values

An abnormal laboratory value is considered to be an AE if the abnormality:

- results in discontinuation from the study;
- requires treatment, modification/interruption of study drug dose, or any other therapeutic
- intervention; or
- is judged to be of significant clinical importance.

Regardless of severity grade, only laboratory abnormalities that fulfill a seriousness criterion need to be documented as a serious adverse event.

If a laboratory abnormality is one component of a diagnosis or syndrome, then only the diagnosis or syndrome should be recorded on the AE page/screen of the CRF. If the abnormality was not a part of a diagnosis or syndrome, then the laboratory abnormality should be recorded as the AE. If possible, the laboratory abnormality should be recorded as a medical term and not simply as an abnormal laboratory result (e.g., record thrombocytopenia rather than decreased platelets).

8.4 Pregnancy

All pregnancies or suspected pregnancies occurring in either a female subject or partner of a male subject are immediately reportable events.

Females of childbearing potential

Pregnancies and suspected pregnancies (including elevated βhCG or a positive pregnancy test regardless of age or disease state) of a female subject occurring while the subject is on study drug, within 28 days of the subject's last dose of study drug, are considered immediately reportable events. The study drug is to be discontinued immediately. The pregnancy, suspected pregnancy, or positive pregnancy test must be reported to Sponsor immediately by facsimile, or other appropriate method, using the Pregnancy Initial Report Form, or approved equivalent form.

The female subject may be referred to an obstetrician-gynecologist (not necessarily one with reproductive toxicity experience) or another appropriate healthcare professional for further evaluation.

The investigator will follow the female subject until completion of the pregnancy, and must notify Sponsor immediately about the outcome of the pregnancy (either normal or abnormal outcome) by email, fax or phone and by sending a completed Pregnancy Report Form or approved equivalent Form to Sponsor..

If the outcome of the pregnancy was abnormal (e.g., spontaneous or therapeutic abortion), the investigator should report the abnormal outcome as an AE. If the abnormal outcome meets any of the serious criteria, it must be reported as an SAE to Sponsor, within 24 hours of the investigator's knowledge of the event using the SAE Report Form, or approved equivalent form.

All neonatal deaths that occur within 28 days of birth should be reported, without regard to causality, as SAEs. In addition, any infant death after 28 days that the investigator suspects is related to the in utero exposure to the study drug should also be reported to Sponsor, within 24 hours of the investigator's knowledge of the event using the SAE Report Form, or approved equivalent form.

Male subjects

If a female partner of a male subject taking investigational product becomes pregnant, the male subject taking the study drug should notify the investigator, and the pregnant female partner should be advised to call their healthcare provider immediately.

If a pregnancy related event is reported in a female partner of a male subject, the investigator should ask if the female partner is willing to share information with the Sponsor and allow the pregnancy related event to be followed up to completion.

8.5 Reporting of serious adverse events

Any AE that meets any criterion for an SAE requires the completion of an SAE Report Form in addition to being recorded on the AE form/screen of the CRF.

All SAEs (initial and follow up information) must be reported to the Sponsor within 24 hours of the Investigator's knowledge of the event by fax or email, using the SAE Report Form.

The investigator is required to ensure that the data on these forms are accurate and consistent.

This requirement applies to all SAEs (regardless of relationship to study drug) that occur during the study (from the time the parent/guardian/subject signs informed consent (as applicable) until 28 days after the last dose of study drug or any SAEs made known to the Investigator at any time thereafter that are suspected of being related to study drug.

SAEs occurring after signing the informed consent but prior to treatment will also be reported to the Sponsor.

The SAE report should provide a detailed description of the SAE and include a concise summary of hospital records and other relevant documents. Hospital records or other relevant documents should not be sent to Sponsor. If a subject died and an autopsy has been performed, copies of the autopsy report and death certificate are to be sent to the Sponsor

Where required by local legislation, the investigator is responsible for informing the Ethics Committee (EC) / Institutional Review Board (IRB) of the SAE and providing them with all relevant initial and follow-up information about the event. The investigator must keep copies of all SAE information on file including correspondence with Sponsor and the EC/IRB.

8.6 Expedited reporting of adverse events

For countries within the European Economic Area (EEA), the Sponsor (or its authorized representative) will report in an expedited manner to Regulatory Authorities and Ethics Committees concerned, suspected unexpected serious adverse reactions (SUSARs) in accordance with Directive 2001/20/EC and the Detailed Guidance on collection, verification and presentation of adverse reaction reports arising from clinical trials on investigational products for human use and also in accordance with country-specific requirements.

The Sponsor will inform relevant Regulatory Authorities and Ethics Committees:

- Of all relevant information about serious unexpected adverse events suspected to be related to the study drug that are fatal or life-threatening as soon as possible, and in any case no later than seven days after knowledge of such a case. Relevant follow-up information for these cases will be subsequently be submitted within an additional eight days
- Of all other serious unexpected events suspected to be related to the study drug as soon as possible, but within a maximum of fifteen days of first knowledge by the investigator.

The Sponsor or its authorized representative shall notify the investigator of the following information:

- Any AE suspected of being related to the use of study drug in this study or in other studies that is both serious and unexpected (i.e., SUSAR);
- Any finding from tests in laboratory animals that suggests a significant risk for human subjects including reports of mutagenicity, teratogenicity, or carcinogenicity.

Where required by local legislation, the investigator shall notify his/her EC/IRB promptly of these new serious and unexpected AE(s) or significant risks to subjects.

The Investigator must keep copies of all pertinent safety information on file.

9. DATA MANAGEMENT

9.1 Electronic case report form (e-CRF)

In this protocol the term electronic case report form (e-CRF) refers to a web-based electronic data record in which the subject data will be collected.

An e-CRF is required to be completed by each participating site for each recruited subject. Only duly authorized and trained staff of each center will be granted access to enter and modify data in the e-CRF. Staff in charge of e-CRF usage will be given a unique user name and password.

The completed original electronic CRF files are the sole property of the Sponsor and should not be made available in any form to third parties, except for authorized representatives of the Sponsor or regulatory authorities, without written permission from the Sponsor.

The investigators have the ultimate responsibility for the collection and reporting of all clinical, safety and laboratory data entered on the e-CRFs and any other data collection forms (source documents) and ensuring that they are accurate, authentic, attributable, complete, consistent, legible, timely, enduring and available when requested. The e-CRF must be signed electronically by the investigator or by an authorized staff member to attest that the data contained in the e-CRF is true. Any changes to entries made in the e-CRF will be tracked by an audit trail system that will identify the old and new values of the data fields, the person who made the change and the reason of modification (if necessary), thus not obscuring the original entry.

The data will be recorded in the e-CRF GCP-compliantly at the study center. The e-CRF application is designed to be entirely server-based. All stages in the processing, with the exception of the actual data entry and display, are performed centrally on a web/database server. All the data will be stored in the central server. The server will be securely housed at a professional hosting provider hired by the study CRO in Spain, guaranteeing effective security and backup mechanisms.

For data entry and print-outs, the e-CRF system is based fully on the so-called "web interface". Entry forms and reports are displayed on the client computer as HTML pages via any Web browser (IE 6+, FireFox 2+, Chrome, Safari 3+). No additional software is necessary to operate the e-CRF on the investigator's client computer (no plugin installations or software/hardware adaptations needed). The e-CRF is operating system independent (Win/Mac).

The data will be checked for correctness by validity and consistency checks. Implausible or missing data can be corrected or supplemented following discussion with the investigator. All corrections will be tracked and stored by the audit trail system.

Other than the investigator, only expressly authorized persons trained for the study may complete the e-CRF. Access control will be implemented by an auditable registry of user logins and logouts. Automatic session logouts will be implemented after predefined periods of inactivity for security reasons. The e-CRF will not show subject personal identification data (each subject will be identified by a unique trial number only).

A comprehensive Data Management Plan will be developed by the study CRO specifying e-CRF/Database design and data-related procedures and policies.

9.2 Data reviews

The study data will be reviewed by telephone calls and periodic inspection of the e-CRF with enough frequency to ascertain the following:

- Investigational medicinal product (IMP) storage conditions, sufficiency of drug supply for the trial.
- Compliance with approved protocol and all approved amendments, if any.
- That the investigator receives all documents and all trial supplies needed to conduct the trial properly and to comply with the applicable regulations.

- That the investigator and trial staff is adequately informed about the trial.
- Integrity and accuracy of data:

  - Informed consent (version, signature and date)
  - Eligibility criteria
  - Baseline tests
  - Adverse event collection
  - SAE and SAE reporting
  - Biological samples storage and collection
  - Drug stock reconciliation in pharmacy

- e-CRF completion
- Reporting of protocol deviations according GCPs and the applicable regulatory local requirements. Taking appropriate action to prevent recurrence to the detected deviations.

The study appointed CRO will review the e-CRFs for compliance with the protocol, and for inconsistent or missing data. Should any missing data or data anomalies be found, queries will be sent to the relevant center for resolution. Any systematic inconsistencies identified may trigger monitoring visits to centers. Following the required reviews, the e-CRF data items will be exported into the clinical study database for further analysis.

9.3 Record keeping

To permit evaluations, audits and/or inspections from regulatory authorities or the Sponsor, investigators agree to keep records, including the identity of all participating subjects (e.g. enough information to link records), all original signed informed consent documents, safety reporting forms, source documents and appropriate documentation of relevant correspondence (e.g. letters, meeting minutes, telephone call reports). The records should be kept by the investigator according to International Conference on Harmonisation (ICH) and local regulations. In case an investigator becomes unable for any reason to continue the retention of study records for the required period, the Sponsor should be prospectively notified. The study records must be transferred to a designee acceptable to the Sponsor. Investigators must receive the Sponsor's written permission before disposing of any records.

10. STUDY DESIGN AND STATISTICS

10.1 Study design

GEIS-39 is a phase II, open-label, non-randomized, multicenter clinical trial with two cohorts. The study will be developed with hospitals in Spain, during a recruitment period of 30 months. The study drug will be investigated separately in the cohorts.

10.2 Study endpoints

Cohort 1: Desmoid tumor

Primary clinical study endpoint

- Overall response rate (ORR) (confirmed complete response [CR] and partial response [PR]), measured using RECIST 1.1 criteria. Response criteria will be based on the baseline identification of target lesions and radiological assessments every 3 months until tumor progression.
- Clinical benefit rate (CBR), measured as CR+PR+SD for 3 months with improvement of pain of at least 2 points in the Brief Pain Inventory – Short Form (BPI-SF).

Secondary clinical study endpoints

- Pattern of radiological response according to MRI parameters (decrease of contrast enhancement in T1-Gd wi, decrease of high signal in T2-wi; increase of low signal bands; increase of the ADC score of diffusion) correlated with CBR and BPI parameters.
- Efficacy measured by the progression-free survival (PFS) rate assessed by median time.
- Variation of symptoms during the first year from trial enrollment, measured with BPI and Analgesic Quantification Algorithm (AQA).
- Variation of physical function during the first year from trial enrollment, assessed every 3 months.
- Safety profile of nab-paclitaxel, through assessment of adverse event type, incidence, severity, time of appearance, related causes, as well as physical explorations and laboratory tests. Toxicity will be graded and tabulated by using NCI-CTCAE 4.0.

Cohort 2: Desmoplastic small round cell tumor and Ewing's sarcoma

Primary clinical study endpoint

- Objective response rate (ORR) (confirmed complete response [CR] and partial response [PR]), measured using RECIST 1.1 criteria. Response criteria will be based on the baseline identification of target lesions and radiological assessments every 8 weeks until tumor progression.

Secondary clinical study endpoint

- Safety profile of nab-paclitaxel, through assessment of adverse event type, incidence, severity, time of appearance, related causes, as well as physical explorations and laboratory tests. Toxicity will be graded and tabulated by using NCI-CTCAE 4.0.

10.3 Sample size

For sample size calculation, a Simon two-stage admissible design[77] is used for cohort 1 (DT), and a Simon two-stage Minimax design is used for cohort 2 (DSRCT and ES). Sample size was calculated using error rates alpha equal to 5% and beta equal to 20%. The total estimated sample size is 60 treated patients (35 for cohort 1 and 25 for cohort 2).

Cohort 1: Desmoid tumor

Option related to efficacy in ORR and/or CBR with pain improvement: 40% - Sample size: 35 subjects

Success (defined as RECIST 1.1 response and/or CBR with pain improvement) in 20% of the cases or less will be considered as unacceptable and would not warrant further investigation (null hypothesis). Therefore, the value of P0 will be taken as 20% (RECIST 1.1 response and/or CBR with pain improvement).

Success (defined as RECIST 1.1 response and/or CBR with pain improvement) in 40% of the cases or more will be considered as an acceptable result warranting further investigation of the drug in this histology (alternative hypothesis). Therefore, the value of P1 will be taken as 40% (RECIST 1.1 response and/or CBR with pain improvement).

A total of 21 eligible and treated subjects will be included in the first stage of the study. If ≤ 4 RECIST 1.1 responses and/or CBR with pain improvement are observed the trial will be stopped in this cohort with the conclusion that the drug should not be further investigated.

Else (>4 RECIST 1.1 responses and/or CBR with pain improvement are observed), subjects will continue to be accrued until 35 eligible subjects enter the study. If 12 or more successes are observed in those 35 subjects, it will be concluded that the results of this trial warrant further investigation.

Cohort 2: DSRCT at least 2nd line and Ewing's sarcoma at least 3rd line

Option related to efficacy: 30% - Sample size: 25 subjects

Success (defined as RECIST 1.1 response) in 10% of the cases or less will be considered as unacceptable, and would not warrant further investigation (null hypothesis). Therefore, the value of P0 will be taken as 10%. Success in 30% of the cases or more will be considered as an acceptable result warranting further investigation of the drug in this histology (alternative hypothesis). Therefore, the value of P1 will be taken as 30%.

A total of 16 eligible and treated subjects will be included in the first stage of the study. If 1 or less successes are observed, the trial will be stopped in this cohort with the conclusion that the drug should not be further investigated. Else (>1 successes), subjects will continue to be accrued until 25 eligible subjects enter the study. If 5 or more successes are observed in those 25 subjects, it will be concluded that the results of this trial warrant further investigation.

10.4 Efficacy analysis

Disease should be captured (with MRI/CT scan) and target/non-target identified at baseline. All the baseline disease assessments should be completed within 28 days prior to the date of inclusion in the trial. Subsequently, imaging studies required to investigate known disease should be done at the end of the third cycle in the case of DT and every 8 weeks in the case of DSRCT and ES (until tumor progression is documented in subjects with complete response, partial response or stable disease). Subjects who have not progressed but discontinued treatment due to toxicity or other reasons unrelated to tumor progression will still be re-evaluated every 3 months (in the case of DT) and every 2 months (in the case of DSRCT and ES), unless they have started a new anti-cancer therapy.

10.5 Safety analysis

Any subject included in the study receiving at least a single dose of study medication will be evaluable for the toxicity analysis. Safety profile will be characterized by treatment-emergent Adverse Events (TEAE), vital signs and laboratory abnormalities. Assessment of adverse events will include type, incidence, severity (graded by the National Cancer Institute [NCI] Common Terminology Criteria for Adverse Events [CTCAE], Version 4.0), timing, seriousness, and relatedness; and laboratory abnormalities. Baseline tumor-related signs and symptoms will be recorded as adverse events during the trial if they worsen in severity or increase in frequency. In each study visit all adverse events will be registered according to NCI-CTC version 4.0.

11. TRANSLATIONAL STUDY

11.1 Translational objectives

Based on the rationale described in the introduction of this protocol, the following translational objectives are defined:

DT Cohort

1.- To analyze protein levels of potential predictive and/or prognostic biomarkers of the response to nab-paclitaxel. The biomarkers (β-catenin, APC, CD105, SPARC, MMP-7, FAS, FASL, THBS1, VEGF and VEGFR1-2) will be evaluated by tissue microarray (TMA)/ immunohistochemistry (IHC) from paraffin block tumor biopsies samples and/ or by ELISA from peripheral blood plasma samples.
2.- To analyze the role of nab-paclitaxel in modulating the expression of soluble factors (CD105, SPARC, FASL and VEGF). Soluble factors expression will be determined by ELISA and qRT-PCR from peripheral blood samples.
3.- To correlate protein expression of potential predictive and/ or prognostic biomarkers of the response to nab-paclitaxel (β-catenin, APC, CD105, SPARC, FAS, FASL, MMP-7, THBS1, VEGF and VEGFR1-2), with clinical endpoint (response rate, time to response, number of lines etc.) in DT.

DSRCT and ES Cohort

1.- To correlate the activity of nab-paclitaxel against DSRCT and Ewing's sarcoma with the expression of SPARC by immunohistochemistry.
2.- To correlate the activity of nab-paclitaxel against DSRCT and Ewing's sarcoma with the loss of p16.
3.- To generate DSRCT and Ewing's sarcoma tumor xenografts from subjects being entered into the trial and evaluate the activity of nab-paclitaxel by preclinical pharmacology.

11.2 Methods and experiments

11.2.1 DT Cohort

Protein expression experiments:

Immunohistochemistry (IHC)

IHC studies will be performed on formalin-fixed, paraffin embedded tumor blocks. Briefly, sections will be sliced (3-4µm thick) and transferred to positively charged-surface glass slides and they will be dried ON, at 62ºC. Next, the sections will be dewaxed and rehydrated through an increased series of ethanol and will be washed with PBS. To desmask antigens, the slides will be treated in a PT Link of DAKO for one cycle of 20min, at 95ºC, without boiling and with a buffer pH8.0. Subsequently, the sections will be left tempering and once again washed with PBS. Immunodetection will be performed with the DAKO EnVision Visualization Method (DAKO), with diaminobenzidine chromogen as substrate. The sections will be counterstained with hematoxylin. The antibodies used in this study will recognize the following antigens: β-catenin, APC, CD105, SPARC, FAS, FASL, MMP-7, THBS1, VEGF and VEGFR1-2.

Tissue microarrays (TMAs)

All the samples obtained from paraffin-embedded tumor blocks will be include in a TMA. This research tool allows the inclusion of many different tumor samples to be studied at the same time and with a high level of standardization. Furthermore, they only require small amounts of reagent and minimal laboratory personnel, which decreases the associated costs. Briefly, TMA consists of carefully placing, in the form of a cylinder of tumor tissue, a high number of samples in one paraffin embedded tissue. Thus, all the samples will be processed under the same technical conditions. When possible, two different tumor regions will be studied in each block.

Protocol for the construction of the tissue microarray

Once the material and both the X and Y axis of the tissue microarray are measured (MTA-1 Manual Tissue Arrayer, Beecher Instruments; USA), the TMA will be constructed as follows:

- Collection of blocks and preparation: before the construction of the TMEA, the blocks from different centers will be shipped to and stored at HUVR. The blocks will be revised by a pathologist expert in sarcomas and a haematoxylin-eosin staining will be carried out on each block in order to check for the existence of the tumor and to select the two representative areas.
- Design of the microarray: the TMA will be constructed with all the cases of study and with a control of known reactivity. The diameter of each cylinder in our study will be of 0.5mm. It will be prepared a computer template, to identify the exact position of each sample in the array.
- Construction of the microarray: an appropriate set of needles will be selected (1mm diameter). Then, the thickest one will be placed in the appropriate position (left) to take a cylinder of tissue from the donor block, and the one which will make the hole in the receptor block, on the right. The perforation will be made in the receptor block, rotating the piston of the needle to reach a depth of approximately 3mm. Afterwards, the donor tissue block will be sited above the bridge, the needles will be changed and the selected tumor tissue will be placed. Next, the bridge is removed with the donor block and the cylinder of tissue is expelled exactly over the hole (in paraffin). This process will be repeated until all the cases are completed.
- 3µm sections will be taken from each tissue microarray and will be collected on poly-lysine slides (DAKO) for staining with H&E and further IHC assays.

Peer Review: To improve the reliability of the IHC analysis, the results will be read by two pathologists, experts in sarcomas. Additionally, it will be reviewed samples from all surfaces of the block (one sample for every 5 cases) in order to validate the results.

ELISA

The protein levels of Endoglin, FASL, VEGF and SPARC will be also evaluated, by ELISA, from plasma samples. Plasma will be isolated from peripheral blood by centrifugation at 3,000 rpm, during 5 minutes, at 4 ºC. The protein levels of Endoglin will be evaluated using the Human Endoglin/CD105 Quantikine ELISA Kit (R&D Systems, Minneapolis, MN, USA), FasL will be evaluated using the Human Fas Ligand/TNFSF6 Quantikine ELISA Kit (R&D Systems), SPARC will be evaluated using the Human SPARC Quantikine ELISA Kit (R&D Systems) and the protein levels of VEGF will be evaluated using the Human VEGF Quantikine ELISA Kit (R&D Systems). All the ELISA experiments will be performed following the manufacturer's instructions.

For RNA expression experiments:

RNA extraction

mRNA expression levels will be evaluated from peripheral blood samples. Total RNA will be extracted using the PAXgene RNA Kit Blood (PreAnalytiX; Hombrechtikon, Switzerland), following the manufacturer's instructions. RNA concentration will be measured at 260 and 280nm using the Nanodrop® 1000 spectrophotometer (Thermo Scientific; Waltham, MA, USA).

Reverse transcription

Reverse transcription will be performed from 40 ng of total RNA using the High Capacity cDNA Reverse Transcription Kit® (Applied Biosystems; Foster City, CA, USA), following manufacturer's protocol.

qRT-PCR

Expression levels of *CD105* (Endoglin), *FASL*, *VEGFA* and *SPARC* will be determined in triplicate, by qRT-PCR, in 10 µl reactions, containing: 2 µl of the synthesized cDNA, 5 µl of TaqMan Fast Universal PCR

Master Mix (Applied Biosystems) and 1µl of the corresponding expression assay (Applied Biosystems). All PCR reactions will be carried out on a 7500 Fast Real Time PCR system (Applied Biosystems). The expression of these genes will be normalized to β2-microglobulin mRNA levels (Hs99999907_m1, Applied Biosystems,). Threshold Cycle data will be analyzed as follow: Ratio= $((Etarget)^{\Delta CPtarget(control\text{-}sample)}) / ((Eref)^{\Delta CPref(control\text{-}sample)})$, in order to quantify gene expression changes during treatment. Expression levels will be expressed in Log2 ratios.

11.2.2 DSRCT and ES Cohort

Regular immunohistochemistry for SPARC and FISH for p16 will be performed on FFPE sections from tumors of subjects entered into the trial. The ideal would be new biopsies from the tumor at progression or at relapse when the subject is being considered for the trial. In its absence, it would be used FFPE sections from the tumor at diagnosis.

Fresh tumor samples will be xenografted into the mice models as described in previous publications of the group (Ordóñez JL, et al. PARP inhibitor Olaparib enhances sensitivity of Ewing's sarcoma cells to Trabectedin: An in vitro and in vivo assay. Oncotarget 2015). Preclinical pharmacology will be developed as previously described (Monterrubio, C., et al., Combined Microdialysis-Tumor Homogenate Method for the Study of the Steady State Compartmental Distribution of a Hydrophobic Anticancer Drug in Subject-Derived Xenografts. Pharm Res, 2015).

11.3 Biological sample collection

The following biological samples will be collected in the study:

<u>DT Cohort</u>

1. One paraffin-embedded tumor block sample collected at diagnosis (the most recent block taken).
2. Two 2.5-mL PAXgene tubes of peripheral blood within 72 hours prior to starting treatment (baseline).
3. Two 2.5-mL PAXgene tubes of peripheral blood at the end of the third 4-week cycle.
4. Two 2.5-mL PAXgene tubes of peripheral blood within 72 hours after first radiological response to nab-paclitaxel is documented.
5. Two 2.5-mL PAXgene tubes of peripheral blood within 72 hours after progressive disease is documented.

<u>DSRCT and ES Cohort</u>

1. 20 slides of FFPE tissues from the most recent biopsy sample of the subject is mandatory to be submitted to the HSJD laboratory for SPARC immunohistochemistry and p16 FISH analysis.
2. A fresh tumor from biopsy should be considered for selected cases for xenografting and preclinical pharmacology modeling. Samples at HSJD will be all processed for xenograft and preclinical pharmacology.

For the shipment of biological samples, please contact:

**Sofpromed Investigación Clínica, SLU**<br>**Telephone: +34** 648 414 261<br>**E-mail:** ensayos@sofpromed.com

An additional document will be provided in the Investigator Site File with detailed guidelines regarding biological sample management.

12. ETHICAL AND REGULATORY ASPECTS

12.1 Ethics committees

The study protocol and/or related documents will be submitted to ethics committees, according to local regulations in Spain, before commencement of the clinical trial. This approval is indispensable for study start-up.

12.2 Competent authorities

The study protocol and/or related documents will be submitted to the regulatory authority before commencement of the clinical trial (Agencia Española de Medicamentos y Productos Sanitarios (AEMPS) of Spain).

12.3 Ethical conduct of the study

This study will be conducted in accordance with the ethical principles that have their origin in the Declaration of Helsinki adopted by the 18th World Medical Association General Assembly, Helsinki, Finland.

The study will be carried out in conformity with the requirements of the "Declaration of Helsinki" adopted by the 18th World Medical Association General Assembly held in Helsinki, Finland, June 1964 and revised by the 29th World Medical Association General Assembly held in October 1975 in Tokyo, the 35th World Medical Association General Assembly held in Venice in October 1983, the 41st World Medical Association General Assembly celebrated in Hong Kong in 1989, the 48th World Medical Association General Assembly held in Somerset West, South Africa in October 1996, the 52nd World Medical Association General Assembly celebrated in Edinburgh, Scotland in October 2000, the 59th World Medical Association General Assembly held in Seoul, Korea, October 2008, the 64th World Medical Association General Assembly held in Fortaleza, Brazil, October 2013; the Good Clinical Practice (GCP) norms issued by the working group on the Efficacy of Medicinal Substances of the European Union (1990) (CPMP/ICH/135/95) and applicable regulatory requirements and laws on the country where the Trial is taking place.

According to Directive 95/46 of the European parliament and 2001/20/EC by which the requirements to perform a clinical trial are established, the information obtained in the course of the clinical trial, will only be able to be used by the clinical trial Sponsor to evaluate the results according to the mentioned regulation.

12.4 Subject privacy

All parties involved in the study must ensure protection of subject personal data and will not include subject names on any Sponsor forms, reports, publications, or in any other disclosures, except when required by laws.

Subject names and other identifiable data will be replaced by an alphanumerical code provided by the Sponsor.

In case of data transfer, the Sponsor will maintain high standards of confidentiality and protection of subject personal data.

In order to warrant the confidentiality of study data according to Directive 95/46 of the European parliament and 2001/20/EC, personal and clinical data can only be accessed by the Sponsor of the study or its designated staff, for monitoring/auditing purposes, the investigator and team of collaborators, the Ethics Committee of the investigational site, or the one overseeing the center, and pertinent Health Authorities.

The investigator should facilitate access to the source documents and data for monitoring and auditing purposes.

The content of the electronic case report forms (e-CRF), as well as the documents generated during the study will be protected from non-permitted uses by persons not involved in the investigation, and will

therefore be considered strictly confidential and not revealed to third parties, except those specified in the previous paragraphs.

12.5 Subject information sheet and consent

The subject information sheet and informed consent documents must be in compliance with ICH GCP guidelines, local regulatory requirements and legal requirements. The information sheets and consents used in the study, and any changes made during its course, must be prospectively approved by the ethics committees before use. Site investigators must ensure that each subject, or the corresponding representatives, is well informed about the nature and the objectives of the study, including potential risks associated with the trial. Investigators, or the persons designated by them, will obtain written informed consents from each subject or from the subject's legal representative before any trial-specific activity begins.

The subject should sign two separate informed consents: one for the clinical trial and another for biological samples studies.

The study subject will provide his/her consent, signing by duplicate the appropriate model. For this purpose, each model must carry the signature of investigator and subject. The investigator will retain one copy of the original of each subject's signed consent form.

The subject must always give his/her written consent before being admitted into the study and before biological samples are taken.

12.6 Insurance policy

A clinical trial insurance policy, in accordance with pertinent regulatory requirements, will be provided for the study in Spain. This policy will be issued and funded by the Sponsor.

13. TRIAL GOVERNANCE AND RESPONSIBILITIES

13.1 Trial Steering Committee (TSC)

A Trial Steering Committee (TSC) will be set up and will include mainly the study coordinating investigators. The trial statistician would also attend and supply the committee with all applicable data and information, for all discussions with the exception of private discussions of the committee.

It is the role of the TSC to monitor progress of the trial and safety of participants, and to ensure there is adherence to the protocol and the principles of Good Clinical Practice.

The TSC's terms of reference, roles and responsibilities will be defined in a charter issued by the Sponsor.

The TSC should meet in confidence at regular intervals, and at least annually. A report of the findings and recommendations will be produced following each meeting. This report will be submitted to the Trial Management Group (TMG) and, if required, to the relevant ethics committees and competent authorities.

13.2 Trial Management Group (TMG)

A Trial Management Group (TMG) will be set up and will include the coordinating investigators and identified collaborators, the trial statistician and trial managers. Selected principal investigators and key study personnel will be invited to join the TMG as appropriate to ensure representation from a range of centers and professional groups. Notwithstanding the legal obligations of the Sponsor and coordinating investigators, the TMG have operational responsibility for the conduct of the trial.

13.3 Sponsor responsibilities

The Sponsor of this clinical trial is the Grupo Español de Investigación en Sarcomas (GEIS). GEIS will delegate on the study CRO a set of responsibilities that will be indicated in an specific agreement. The responsibilities of the Sponsor within the trial will also be specified in the contracts signed with each participating hospital.

13.4 Principal investigators responsibilities

Responsibilities of each Principal Investigator and participating center will be detailed in a contract with the Sponsor or with the delegated country-specific coordinating institution with trial coordination responsibilities in that country.

Principal Investigator responsibilities include putting and keeping in place arrangements to run the trial at their site according to the trial protocol and applicable guidelines, local regulations and the principles of GCP. These responsibilities include, but are not limited to, ensuring that:

- The applicable ethical and institution specific approvals are in place before recruiting subjects;
- Sufficient data is recorded for all subjects to enable accurate linkage between hospital records and e-CRFs;
- Source data and all trial related documentation are accurate, complete, maintained and accessible for monitoring and audit visits;
- All staff involved with the trial are trained in and work to the applicable regulatory requirements;
- Original consent forms are personally signed and dated by both the subject and investigator and are kept together in a central log together with a copy of the specific subject information sheet(s) given at the time of consent;
- All essential documents are retained in accordance with local regulations;
- Staff comply with the protocol and trial guidance notes for the trial;
- SAEs are reported to the study CRO within the required timelines.

14. STUDY DEVELOPMENT CONSIDERATIONS

14.1 Inclusion of the study in clinical trial registries

The clinical trial will be registered in the *clinicaltrials.gov* database of the National Institute of Health of the United States of America, as well as in *clinicaltrialsregister.eu*.

14.2 Quality control and quality assurance

Each study site may be subject to review by the ethics committees and/or to quality assurance audits performed by the Sponsor, or companies working with or on behalf of the Sponsor, and/or to inspection by appropriate regulatory authorities.

The investigators and their staff should be available during these visits, audits or inspections. Sufficient time is to be devoted to these processes.

14.3 Definition of end of study

The study will be considered closed from a normative point of view after data on primary and secondary variables are sufficiently prepared for its initial publication.

14.4 Sponsor discontinuation criteria

Premature termination of this study may occur due to regulatory authority decision, change in opinion of ethics committees, drug safety problems, or at discretion of the Sponsor. If the study is prematurely terminated, the Sponsor will notify the investigators. After notification, the investigator must contact all participating subjects and the hospital. All study materials must be collected and all e-CRFs completed to the fullest extent possible.

This study can be terminated prematurely if in the opinion of the Sponsor there is a reasonable and sufficient cause. Investigators will receive a written notification in which the Sponsor motivates the interruption of the study. Reasons that justify are as follows, but not limited to:

- Finding of unforeseen, considerable or unacceptable risks for the subjects.
- Impossibility to include an acceptable number of subjects.
- Insufficient compliance with protocol requisites.
- Plans to modify halt or discontinue the development of study drug.
- In case of early termination of the study, all the study material (study drugs, etc.) must be returned to the Sponsor.

14.5 Publication of results

The final publication of the trial results will be written by the coordinating investigators on the basis of the final analysis performed.

The draft manuscript will be reviewed by the coordinating investigators and other co-authors. After revision the manuscript will be sent to a major scientific journal. Results obtained in the different strata may be separately published.

Regarding authorship, institutional entry of 5% of evaluable subjects in the study results in qualification for one authorship (two names for 20% entry, up to the number of authors allowed by the journal). The reference pathologists, radiologists and statisticians, who have contributed to the trial, will be included in the authorship of the final manuscript.

All manuscripts will include an appropriate acknowledgement section, mentioning all investigators who have contributed to the trial, as well as supporting parties.

All publications (papers, abstracts, or presentations) including data from the present trial will be submitted for review to all co-authors prior to submission.

Results of the translational research will be published in major scientific journals, after the publication of the main results of the clinical study. The first authors will be the translational study coordinators. All centers that have contributed to at least 5% of the analyzed material will be represented in the publication by one co-author; centers that have contributed to at least 15% of the analyzed material will be represented by two co-authors; this(these) author(s) will be selected by each center internally (e.g. pathologist, molecular biologist, clinician). All centers that have provided material for the analysis will be acknowledged.

APPENDIX A: PAIN AND SYMPTOMS ASSESSMENT

Subjects assessed their pain with the Brief Pain Inventory – Short Form (BPI-SF) at baseline and before treatment on days 8 and 15 every four weeks in months 1 – 3, and then every month until the first year is completed. Subjects rated pain severity at its worst, at its least, on average, and now on an 11-point scale (0, no pain; 1 – 4, mild pain; 5 – 6, moderate pain; 7 – 10, severe pain). The clinical relevance of changes in scores was determined based on the minimally important difference (MID), the smallest difference that subjects perceived as important and that would have led the clinician to consider a change in the subject' s management. The MID for BPI-SF within this study was conservatively defined as 2 based on anchor-based and distribution-based methods. Analgesic use was determined at each visit from concomitant medication records and scored with the Analgesic Quantification Algorithm (AQA), an 8-point scale. AQA scores of _ 2 points were categorized as no/low analgesic use, and scores _ 3 points as strong opioid use.

Pre-specified endpoints were the proportion of subjects with a clinically relevant increase or decrease in worst pain (≥ 2-point increase or decrease from baseline, respectively) and the time to clinically relevant increase or decrease in worst pain (≥ 2 point increase or decrease from baseline, respectively), the proportions of subjects with moderate or severe worst pain at baseline (worst pain score ≥ 4 points) who shifted to no or mild pain (worst pain score ≥ 4 points) during the study, the proportions of subjects with a shift from strong opioid use (score ≥ 3) to no/low analgesic use (score ≥ 2), and the proportion of subjects with a shift from no/low analgesic use to strong opioid use.

AQA Score	Description
0	No analgesic
1	Non-opioid analgesics
2	Weak opioids*
3	Strong opioids ≤75 mg OME per day
4	Strong opioids >75–150 mg OME per day
5	Strong opioids >150–300 mg OME per day
6	Strong opioids >300–600 mg OME per day
7	Strong opioids >600 mg OME per day

* For example, codeine and tramadol.
AQA = Analgesic Quantification Algorithm; OME = oral morphine equivalent.

	Equianalgesic Dose (mg)				
Name	IV	SC	IM	PO	OME
Morphine	10	10	10	30	30
Fentanyl*	0.1	0.1	0.1	2.4	30
Hydromorphone	1	1	1	5	20
Methadone	10	10	10	20	30
Oxycodone	—	—	—	20	30
Hydrocodone	—	—	—	40	40
Codeine	120	120	120	200	30
Tramadol	100	100	100	100	30
Buprenorphine	0.3	0.3	0.3	0.2	30
Butorphanol	—	—	2	—	30
Nalbuphine	10	10	10	—	30
Pentazocine	60	60	60	60	20

* Fentanyl, 0.1 mg transdermal has an OME of 30 mg.
IV = intravenous; SC = subcutaneous; IM = intramuscular; OME = oral morphine equivalent; PO = oral.

APPENDIX B: DETERMINATION OF CREATININE CLEARANCE (Cl_{CR})

Estimation of creatinine clearance using Cockroft and Gault method:

$$Cl_{CR} \text{ for males (mL/min)} = \frac{[140 - \text{age (years)}] \times [\text{weight (kg)}]}{(72) \times [\text{Serum creatinine (mg/dL)}]}$$

$$Cl_{CR} \text{ for females (mL/min)} = \frac{(0.85) \times [140 - \text{age (years)}] \times [\text{weight (kg)}]}{(72) \times [\text{Serum creatinine (mg/dL)}]}$$

For SI units:

$$Cl_{CR} \text{ for males (mL/min)} = \frac{[140 - \text{age (years)}] \times [\text{weight(kg)}] \times (1.23)}{[\text{Serum creatinine (µmol/L)}]}$$

$$Cl_{CR} \text{ for females (mL/min)} = \frac{[140 - \text{age (years)}] \times [\text{weight(kg)}] \times (1.05)}{[\text{Serum creatinine (µmol/L)}]}$$

Calculation of creatinine clearance based on 24-hour urinary creatinine excretion and concurrent serum creatinine levels:

$$Cl_{CR} = (C_U \cdot V) / C_{CR}$$

Here, C_U is the concentration of creatinine in the urine (mg/dL or µmol/L, for SI units), V is the urine volume (in mL per minute of urine produced during the collection period), C_{CR} is the serum creatinine concentration (mg/dL or µmol/L, for SI units), and Cl_{CR} is the creatinine clearance in mL per minute.

APPENDIX C: DECLARATION OF HELSINKI, WORLD MEDICAL ASSOCIATION GENERAL ASSEMBLY

Adopted by the 18th World Medical Association General Assembly held in Helsinki, Finland, June 1964 and revised by the 29th World Medical Association General Assembly held in October 1975 in Tokyo,
the 35th World Medical Association General Assembly held in Venice in October 1983,
the 41st World Medical Association General Assembly celebrated in Hong Kong in 1989,
the 48th World Medical Association General Assembly held in Somerset West, South Africa in October 1996,
the 52nd World Medical Association General Assembly celebrated in Edinburgh, Scotland in October 2000,
Paragraph 29 Clarification note, added by the World Medical Association General Assembly, Washington 2002,
the 59th World Medical Association General Assembly held in Seoul, Korea, October 2008,
and the 64th World Medical Association General Assembly held in Fortaleza, Brazil, October 2013.

INTRODUCTION

The World Medical Association (WMA) has developed the Declaration of Helsinki as a statement of ethical principles for medical research involving human subjects, including research on identifiable human material and data.

The Declaration is intended to be read as a whole and each of its constituent paragraphs should not be applied without consideration of all other relevant paragraphs.

Although the Declaration is addressed primarily to physicians, the WMA encourages other participants in medical research involving human subjects to adopt these principles.

It is the duty of the physician to promote and safeguard the health of subjects, including those who are involved in medical research. The physician's knowledge and conscience are dedicated to the fulfilment of this duty.

The Declaration of Geneva of the WMA binds the physician with the words, "The health of my subject will be my first consideration," and the International Code of Medical Ethics declares that, "A physician shall act in the subjectsubject's best interest when providing medical care."

Medical progress is based on research that ultimately must include studies involving human subjects. Populations that are under-represented in medical research should be provided appropriate access to participation in research.

In medical research involving human subjects, the well-being of the individual research subject must take precedence over all other interests.

The primary purpose of medical research involving human subjects is to understand the causes, development and effects of diseases and improve preventive, diagnostic and therapeutic interventions (methods, procedures and treatments). Even the best current interventions must be evaluated continually through research for their safety, effectiveness, efficiency, accessibility and quality.

In medical practice and in medical research, most interventions involve risks and burdens. Medical research is subject to ethical standards that promote respect for all human subjects and protect their health and rights. Some research populations are particularly vulnerable and need special protection. These include those who cannot give or refuse consent for themselves and those who may be vulnerable to coercion or undue influence.

Physicians should consider the ethical, legal and regulatory norms and standards for research involving human subjects in their own countries as well as applicable international norms and standards. No national

or international ethical, legal or regulatory requirement should reduce or eliminate any of the protections for research subjects set forth in this Declaration.

PRINCIPLES FOR ALL MEDICAL RESEARCH

It is the duty of physicians who participate in medical research to protect the life, health, dignity, integrity, right to self-determination, privacy, and confidentiality of personal information of research subjects.

Medical research involving human subjects must conform to generally accepted scientific principles, be based on a thorough knowledge of the scientific literature, other relevant sources of information, and adequate laboratory and, as appropriate, animal experimentation. The welfare of animals used for research must be respected.

Appropriate caution must be exercised in the conduct of medical research that may harm the environment.

The design and performance of each research study involving human subjects must be clearly described in a research protocol. The protocol should contain a statement of the ethical considerations involved and should indicate how the principles in this Declaration have been addressed. The protocol should include information regarding funding, sponsors, institutional affiliations, other potential conflicts of interest, incentives for subjects and provisions for treating and/or compensating subjects who are harmed as a consequence of participation in the research study. The protocol should describe arrangements for post-study access by study subjects to interventions identified as beneficial in the study or access to other appropriate care or benefits.

The research protocol must be submitted for consideration, comment, guidance and approval to a research ethics committee before the study begins. This committee must be independent of the researcher, the sponsor and any other undue influence. It must take into consideration the laws and regulations of the country or countries in which the research is to be performed as well as applicable international norms and standards but these must not be allowed to reduce or eliminate any of the protections for research subjects set forth in this Declaration. The committee must have the right to monitor ongoing studies. The researcher must provide monitoring information to the committee, especially information about any serious adverse events. No change to the protocol may be made without consideration and approval by the committee.

Medical research involving human subjects must be conducted only by individuals with the appropriate scientific training and qualifications. Research on subjects or healthy volunteers requires the supervision of a competent and appropriately qualified physician or other health care professional. The responsibility for the protection of research subjects must always rest with the physician or other health care professional and never the research subjects, even though they have given consent.

Medical research involving a disadvantaged or vulnerable population or community is only justified if the research is responsive to the health needs and priorities of this population or community and if there is a reasonable likelihood that this population or community stands to benefit from the results of the research.

Every medical research study involving human subjects must be preceded by careful assessment of predictable risks and burdens to the individuals and communities involved in the research in comparison with foreseeable benefits to them and to other individuals or communities affected by the condition under investigation.

Every clinical trial must be registered in a publicly accessible database before recruitment of the first subject.

Physicians may not participate in a research study involving human subjects unless they are confident that the risks involved have been adequately assessed and can be satisfactorily managed. Physicians must immediately stop a study when the risks are found to outweigh the potential benefits or when there is conclusive proof of positive and beneficial results.

Medical research involving human subjects may only be conducted if the importance of the objective outweighs the inherent risks and burdens to the research subjects.

Participation by competent individuals as subjects in medical research must be voluntary.

Although it may be appropriate to consult family members or community leaders, no competent individual may be enrolled in a research study unless he or she freely agrees.

Every precaution must be taken to protect the privacy of research subjects and the confidentiality of their personal information and to minimize the impact of the study on their physical, mental and social integrity.

In medical research involving competent human subjects, each potential subject must be adequately informed of the aims, methods, sources of funding, any possible conflicts of interest, institutional affiliations of the researcher, the anticipated benefits and potential risks of the study and the discomfort it may entail, and any other relevant aspects of the study. The potential subject must be informed of the right to refuse to participate in the study or to withdraw consent to participate at any time without reprisal. Special attention should be given to the specific information needs of individual potential subjects as well as to the methods used to deliver the information. After ensuring that the potential subject has understood the information, the physician or another appropriately qualified individual must then seek the potential subject's freely-given informed consent, preferably in writing. If the consent cannot be expressed in writing, the non-written consent must be formally documented and witnessed.

For medical research using identifiable human material or data, physicians must normally seek consent for the collection, analysis, storage and/or reuse. There may be situations where consent would be impossible or impractical to obtain for such research or would pose a threat to the validity of the research. In such situations the research may be done only after consideration and approval of a research ethics committee.

When seeking informed consent for participation in a research study the physician should be particularly cautious if the potential subject is in a dependent relationship with the physician or may consent under duress. In such situations the informed consent should be sought by an appropriately qualified individual who is completely independent of this relationship.

For a potential research subject who is incompetent, the physician must seek informed consent from the legally authorized representative. These individuals must not be included in a research study that has no likelihood of benefit for them unless it is intended to promote the health of the population represented by the potential subject, the research cannot instead be performed with competent persons, and the research entails only minimal risk and minimal burden.

When a potential research subject who is deemed incompetent is able to give assent to decisions about participation in research, the physician must seek that assent in addition to the consent of the legally authorized representative. The potential subject's dissent should be respected.

Research involving subjects who are physically or mentally incapable of giving consent, for example, unconscious subjects, may be done only if the physical or mental condition that prevents giving informed consent is a necessary characteristic of the research population. In such circumstances the physician should seek informed consent from the legally authorized representative. If no such representative is available and if the research cannot be delayed, the study may proceed without informed consent provided that the specific reasons for involving subjects with a condition that renders them unable to give informed consent have been stated in the research protocol and the study has been approved by a research ethics committee. Consent to remain in the research should be obtained as soon as possible from the subject or a legally authorized representative.

Authors, editors and publishers all have ethical obligations with regard to the publication of the results of research. Authors have a duty to make publicly available the results of their research on human subjects and are accountable for the completeness and accuracy of their reports. They should adhere to accepted guidelines for ethical reporting. Negative and inconclusive as well as positive results should be published or otherwise made publicly available. Sources of funding, institutional affiliations and conflicts of interest should be declared in the publication. Reports of research not in accordance with the principles of this Declaration should not be accepted for publication.

PRINCIPLES FOR MEDICAL RESEARCH WHEN COMBINED WITH MEDICAL CARE

The physician may combine medical research with medical care only to the extent that the research is justified by its potential preventive, diagnostic or therapeutic value and if the physician has good reason to believe that participation in the research study will not adversely affect the health of the subjects who serve as research subjects.

The possible benefits, risks, burdens and effectiveness of a new intervention must be tested against those of the best current proven intervention, except in the following circumstances:

The use of placebo, or no treatment, is acceptable in studies where no current proven intervention exists; or.

Where for compelling and scientifically sound methodological reasons the use of placebo is necessary to determine the efficacy or safety of an intervention and the subjects who receive placebo or no treatment will not be subject to any risk of serious or irreversible harm. Extreme care must be taken to avoid abuse of this option.

At the conclusion of the study, subjects entered into the study are entitled to be informed about the outcome of the study and to share any benefits that result from it, for example, access to interventions identified as beneficial in the study or to other appropriate care or benefits.

The physician must fully inform the subject which aspects of the care are related to the research.

The refusal of a subject to participate in a study or the subject's decision to withdraw from the study must never interfere with the subject-physician relationship.

In the treatment of a subject, where proven interventions do not exist or have been ineffective, the physician, after seeking expert advice, with informed consent from the subject or a legally authorized representative, may use an unproven intervention if in the physician's judgment it offers hope of saving life, re-establishing health or alleviating suffering. Where possible, this intervention should be made the object of research, designed to evaluate its safety and efficacy.

In all cases, new information should be recorded and, where appropriate, made publicly available.

REFERENCES

1. Hosalkar HS, Torbert JT, Fox EJ, et al: Musculoskeletal desmoid tumors. J Am AcadOrthopSurg 16:188-98, 2008
2. Nieuwenhuis MH, Casparie M, Mathus-Vliegen LM, et al: A nation-wide study comparing sporadic and familial adenomatous polyposis-related desmoid-type fibromatoses. Int J Cancer 129:256-61, 2011
3. Hartley JE, Church JM, Gupta S, et al: Significance of incidental desmoids identified during surgery for familial adenomatous polyposis. Dis Colon Rectum 47:334-8; discussion 339-40, 2004
4. Meazza C, Bisogno G, Gronchi A, et al: Aggressive fibromatosis in children and adolescents: the Italian experience. Cancer 116:233-40, 2010
5. Lazar AJ, Tuvin D, Hajibashi S, et al: Specific mutations in the beta-catenin gene (CTNNB1) correlate with local recurrence in sporadic desmoid tumors. Am J Pathol 173:1518-27, 2008
6. Carlson JW, Fletcher CD: Immunohistochemistry for beta-catenin in the differential diagnosis of spindle cell lesions: analysis of a series and review of the literature. Histopathology 51:509-14, 2007
7. Domont J, Salas S, Lacroix L, et al: High frequency of beta-catenin heterozygous mutations in extra-abdominal fibromatosis: a potential molecular tool for disease management. Br J Cancer 102:1032-6, 2010
8. Kotiligam D, Lazar AJ, Pollock RE, et al: Desmoid tumor: a disease opportune for molecular insights. HistolHistopathol 23:117-26, 2008
9. Lips DJ, Barker N, Clevers H, et al: The role of APC and beta-catenin in the aetiology of aggressive fibromatosis (desmoid tumors). Eur J SurgOncol 35:3-10, 2009
10. Vane JR, Bakhle YS, Botting RM: Cyclooxygenases 1 and 2. Annu Rev PharmacolToxicol 38:97-120, 1998
11. Hansmann A, Adolph C, Vogel T, et al: High-dose tamoxifen and sulindac as first-line treatment for desmoid tumors. Cancer 100:612-20, 2004
12. Bamias A, Kyriakou F, Chorti M, et al: Microvessel density (MVD) and cyclooxygenase-2 (COX-2)/ beta-catenin interaction are associated with relapse in subjects with transitional carcinoma receiving adjuvant chemotherapy with paclitaxel/carboplatin: a hellenic cooperative oncology group (HECOG) study. Anticancer Res 28:2479-86, 2008
13. Bhatt RS, Merchan J, Parker R, et al: A phase 2 pilot trial of low-dose, continuous infusion, or "metronomic" paclitaxel and oral celecoxib in subjects with metastatic melanoma. Cancer 116:1751-6, 2010
14. Zhong H, Agani F, Baccala AA, et al: Increased expression of hypoxia inducible factor-1alpha in rat and human prostate cancer. Cancer Res 58:5280-4, 1998
15. Kaidi A, Williams AC, Paraskeva C: Interaction between beta-catenin and HIF-1 promotes cellular adaptation to hypoxia. Nat Cell Biol 9:210-7, 2007
16. Toschi A, Lee E, Gadir N, et al: Differential dependence of hypoxia-inducible factors 1 alpha and 2 alpha on mTORC1 and mTORC2. J BiolChem 283:34495-9, 2008
17. Matono H, Oda Y, Nakamori M, et al: Correlation between beta-catenin widespread nuclear expression and matrix metalloproteinase-7 overexpression in sporadic desmoid tumors. Hum Pathol 39:1802-8, 2008
18. Denys H, De Wever O, Nusgens B, et al: Invasion and MMP expression profile in desmoidtumours. Br J Cancer 90:1443-9, 2004
19. Guo XL, Lin GJ, Zhao H, et al: Inhibitory effects of docetaxel on expression of VEGF, bFGF and MMPs of LS174T cell. World J Gastroenterol 9:1995-8, 2003
20. Matono H, Tamiya S, Yokoyama R, et al: Abnormalities of the Wnt/beta-catenin signalling pathway induce tumour progression in sporadic desmoidtumours: correlation between beta-catenin widespread nuclear expression and VEGF overexpression. Histopathology 59:368-75, 2011
21. Wang J, Lou P, Lesniewski R, et al: Paclitaxel at ultra-low concentrations inhibits angiogenesis without affecting cellular microtubule assembly. Anticancer Drugs 14:13-9, 2003
22. Escobar C, Munker R, Thomas JO, et al: Update on desmoid tumors. Ann Oncol 23:562-9, 2012
23. O'Keefe F, Kim EE, Wallace S: Magnetic resonance imaging in aggressive fibromatosis. ClinRadiol 42:170-3, 1990
24. Fiore M, Rimareix F, Mariani L, et al: Desmoid-type fibromatosis: a front-line conservative approach to select subjects for surgical treatment. Ann SurgOncol 16:2587-93, 2009
25. Salas S, Dufresne A, Bui B, et al: Prognostic factors influencing progression-free survival determined from a series of sporadic desmoid tumors: a wait-and-see policy according to tumor presentation. J ClinOncol 29:3553-8, 2011
26. Baumert BG, Spahr MO, Von Hochstetter A, et al: The impact of radiotherapy in the treatment of desmoidtumours. An international survey of 110 subjects.A study of the Rare Cancer Network.RadiatOncol 2:12, 2007
27. Guadagnolo BA, Zagars GK, Ballo MT: Long-term outcomes for desmoid tumors treated with radiation therapy. Int J RadiatOncolBiolPhys 71:441-7, 2008
28. Janinis J, Patriki M, Vini L, et al: The pharmacological treatment of aggressive fibromatosis: a systematic review. Ann Oncol 14:181-90, 2003
29. Skapek SX, Ferguson WS, Granowetter L, et al: Vinblastine and methotrexate for desmoidfibromatosis in children: results of a Pediatric Oncology Group Phase II Trial. J ClinOncol 25:501-6, 2007
30. Gega M, Yanagi H, Yoshikawa R, et al: Successful chemotherapeutic modality of doxorubicin plus dacarbazine for the treatment of desmoid tumors in association with familial adenomatous polyposis. J ClinOncol 24:102-5, 2006
31. Constantinidou A, Jones RL, Scurr M, et al: Pegylated liposomal doxorubicin, an effective, well-tolerated treatment for refractory aggressive fibromatosis. Eur J Cancer 45:2930-4, 2009
32. Penel N, Le Cesne A, Bui BN, et al: Imatinib for progressive and recurrent aggressive fibromatosis (desmoid tumors): an FNCLCC/French Sarcoma Group phase II trial with a long-term follow-up. Ann Oncol 22:452-7, 2011

33. Gradishar WJ, Tjulandin S, Davidson N, et al: Phase III trial of nanoparticle albumin-bound paclitaxel compared with polyethylated castor oil-based paclitaxel in women with breast cancer. J ClinOncol 23:7794-803, 2005
34. Gradishar WJ, Krasnojon D, Cheporov S, et al: Significantly longer progression-free survival with nab-paclitaxel compared with docetaxel as first-line therapy for metastatic breast cancer. J ClinOncol 27:3611-9, 2009
35. Imaging of desmoplastic small round cell tumour in adults. B KIS, K N O'REGAN,A AGOSTON, O JAVERY, J JAGANNATHAN, and N H RAMAIYA. The British Journal of Radiology, 85 (2012), 187–192. DOI: 10.1259/bjr/57186741
36. Review of therapeutic strategies for osteosarcoma, chondrosarcoma and Ewing sarcoma. Xing Dai, Wei Ma, Xijing He,and Rajiv Kumar Jha.Med SciMonit. 2011; 17(8): RA177–RA190. doi: 10.12659/MSM.881893
37. Gerald WL, Miller HK, Battifora H, et al: Intra-abdominal desmoplastic small round-cell tumor. Report of 19 cases of a distinctive type of high-grade polyphenotypic malignancy affecting young individuals.Am J SurgPathol 15:499-513, 1991
38. Gerald WL, Rosai J, Ladanyi M: Characterization of the genomic breakpoint and chimeric transcripts in the EWS-WT1 gene fusion of desmoplastic small round cell tumor. ProcNatlAcadSci U S A 92:1028-32, 1995
39. Gerald WL, Ladanyi M, de Alava E, et al: Clinical, pathologic, and molecular spectrum of tumors associated with t(11;22)(p13;q12): desmoplastic small round-cell tumor and its variants. J ClinOncol 16:3028-36, 1998
40. Schwarz RE, Gerald WL, Kushner BH, et al: Desmoplastic small round cell tumors: prognostic indicators and results of surgical management. Ann SurgOncol 5:416-22, 1998
41. Lal DR, Su WT, Wolden SL, et al: Results of multimodal treatment for desmoplastic small round cell tumors. J PediatrSurg 40:251-5, 2005
42. Desai NB, Stein NF, LaQuaglia MP, et al: Reduced toxicity with intensity modulated radiation therapy (IMRT) for desmoplastic small round cell tumor (DSRCT): an update on the whole abdominopelvic radiation therapy (WAP-RT) experience. Int J RadiatOncolBiolPhys 85:e67-72, 2013
43. Lee SB, Kolquist KA, Nichols K, et al: The EWS-WT1 translocation product induces PDGFA in desmoplastic small round-cell tumour. Nat Genet 17:309-13, 1997
44. Bisogno G, Ferrari A, Rosolen A, et al: Sequential intensified chemotherapy with stem cell rescue for children and adolescents with desmoplastic small round-cell tumor. Bone Marrow Transplant 45:907-11, 2010
45. Kushner BH, LaQuaglia MP, Wollner N, et al: Desmoplastic small round-cell tumor: prolonged progression-free survival with aggressive multimodality therapy. J ClinOncol 14:1526-31, 1996
46. Kushner BH, Laquaglia MP, Gerald WL, et al: Solitary relapse of desmoplastic small round cell tumor detected by positron emission tomography/computed tomography. J ClinOncol 26:4995-6, 2008
47. Magnan H, Abramson SJ, Price AP, et al: Positron emission tomography for response assessment in desmoplastic small round cell tumor. J PediatrHematolOncol 35:e190-3, 2013
48. Chao J, Budd GT, Chu P, et al: Phase II clinical trial of imatinibmesylate in therapy of KIT and/or PDGFRalpha-expressing Ewing sarcoma family of tumors and desmoplastic small round cell tumors. Anticancer Res 30:547-52, 2010
49. Lopez-Gonzalez A, Cantos B, Tejerina E, et al: Activity of trabectidin in desmoplastic small round cell tumor. Med Oncol 28 Suppl 1:S644-6, 2011
50. Chuk MK, Aikin A, Whitcomb T, et al: A phase I trial and pharmacokinetic study of a 24-hour infusion of trabectedin (Yondelis(R), ET-743) in children and adolescents with relapsed or refractory solid tumors. Pediatr Blood Cancer 59:865-9, 2012
51. Italiano A, Kind M, Cioffi A, et al: Clinical activity of sunitinib in subjects with advanced desmoplastic round cell tumor: a case series. Target Oncol 8:211-3, 2013
52. Zhang L, Marrano P, Kumar S, et al: Nab-paclitaxel is an active drug in preclinical model of pediatric solid tumors. Clin Cancer Res 19:5972-83, 2013
53. Tirado OM, MacCarthy CM, Fatima N, et al: Caveolin-1 promotes resistance to chemotherapy-induced apoptosis in Ewing's sarcoma cells by modulating PKCalpha phosphorylation. Int J Cancer 126:426-36, 2010
54. Sainz-Jaspeado M, Martin-Liberal J, Lagares-Tena L, et al: Caveolin-1 in sarcomas: friend or foe? Oncotarget 2:305-12, 2011
55. Mora J, Cruz CO, Parareda A, et al: Treatment of relapsed/refractory pediatric sarcomas with gemcitabine and docetaxel. J PediatrHematolOncol 31:723-9, 2009
56. Rapkin L, Qayed M, Brill P, et al: Gemcitabine and docetaxel (GEMDOX) for the treatment of relapsed and refractory pediatric sarcomas. Pediatr Blood Cancer 59:854-8, 2012
57. Houghton, P.J., et al., The pediatric preclinical testing program: description of models and early testing results. Pediatr Blood Cancer, 2007. 49(7): p. 928-40.
58. Houghton, P.J., et al., Initial testing (stage 1) of the tubulin binding agent nanoparticle albumin-bound (nab) paclitaxel (Abraxane) by the Pediatric Preclinical Testing Program (PPTP). Pediatr Blood Cancer, 2015.
59. Monterrubio, C., et al., Combined Microdialysis-Tumor Homogenate Method for the Study of the Steady State Compartmental Distribution of a Hydrophobic Anticancer Drug in Subject-Derived Xenografts. Pharm Res, 2015.
60. Desai N, Trieu V, Yao Z, Louie L, Ci S, Yang A, et al. Increased antitumor activit y, intratumor paclitaxel concentrations, and endothelial cell transport of cremophor-free, albumin-bound paclitaxel, ABI-007, compared with cremophor-based paclitaxel. Clin Cancer Res
2006;12(4):1317-24.
61. Ibrahim NK, Desai N, Legha S, Soon-Shiong P, Theriault RL, Rivera E, et al. Phase I and Pharmocokinetic Study of ABI-007, a Cremophor-Free, Protein-Stabilized, Nanoparticle Formulation of Paclitaxel. Clin Cancer Res 2002 May;8(5):1038-44.

62. Sparreboom A, van Zuylen L, Brouwer E, Loos W, de Bruijn P, Gelderblom H, et al. Cremophor EL-mediated alteration of paclitaxel distributio n in human blood: clinical pharmacokinet ic implications. Cancer Res 1999;59:1454-57.
63. ten Tije AJ, Verweij J, Loos WJ, Sparreboom A. Pharmacological effects of fo rmulation vehicles: implicatio ns fo r cancer chemotherapy. Clin Pharmacokinet 2003;42(7):665-85.
64. Gardner ER, Dahut WL, Scripture CD, Jones J, Aragon-Ching JB, Desai N, et al. Randomized Crossover Pharmacokinetic Study of Solvent-Based Paclit axel and *nab*-Paclitaxel. ClinCancer Res 2008;14(13):4200-5.
65. Alvarez-Gallego R, Cubillo A, Rodriguez-Pascual J, Quijano Y, De Vicente E, Garcia L, et al. Antitumor activity of nab-paclitaxel and gemcitabine in resectable pancreatic cancer [abstract]. American Society of Clinical Oncology Annual Meeting (ASCO); 2012 Jun 1-5; Chicago, IL. Abstract no. 4040.
66. Blackwell KL, Hamilton EP, Rocha G, Gainey M, Trieu V, Motamed K, et al. SPARC microenvironment signature (SMS) in patie nts treated with nab-paclitaxel (nab-P)/carboplat in (C)/bevacizumab(B) for triple negative metastatic breast cancer (TNMBC) [abstract]. Proceedings of the European Society fo r Medical Oncolo gy Congress; 2010 October 8-12; Milan, Italy. Abstract no. 181.
67. Desai N, Trieu V, Damascelli B, Soon-Shiong P. SPARC Expression Correlates with Tumor Response to Albumin-Bound Paclitaxel in Head and Neck Cancer Patients. Transl Oncol 2009;2(2):59-64.
68. Yardley DA, Raefsky E, Castillo R, Lahiry A, LoCicero R, Thompson D, et al. Results of a multicenter pilot study of weekly nab-paclitaxel, carboplat in with bevacizumab, and trastuzumab as neoadjuvant therapy in HER2+ locally advanced breast cancer with SPARC correlatives. [abstract] Proceedings of the 45th American Society of Clinical Oncolo gy Annual Meeting (ASCO); 2009 May 29 - June 2; Orlando, FL. J Clin Oncol 2009;27(15 suppl):527.
69. Nyman DW, Campbell, KJ, Hersch E, Richardsom, K, Patrick K, Trieu B, et al. A Phase I trial of ABI-007, nanoparticle paclitaxel, administered to subjects with advanced non-hematologic malignancies. J Clin Oncol 2004, ASCO Annual Meeting Proceedings (Post-Meeting Edition);22(14)(15 Jul Suppl):2027.
70. Taxol [package insert]. Princeton (NJ): Bristol-Myers Squibb; 2011.
71. Allwood MC, Martin H. The Extraction of Diethylhexylphthalate (DHEP) from Polyvinyl Chloride Components of Intravenous Infusion Containers and Administration Sets by Paclitaxel Injection. Int J Pharmaceut 1996;127:65-71.
72. Gelderblom H, Verweij J, Nooter K, Sparreboom A. Cremophor EL: the drawbacks and advantages of vehicle selectio n fo r drug formulatio n. Eur J Cancer 2001 Sep;37(13):1590-8.
73. Pfeifer RW, Hale KN, Cronquist SE, Daniels M. Precipitatio n of Paclitaxel During Infusion by Pump. Am J Hosp Pharm 1993 Dec;50:2518-21.
74. Song DI, Hsu Li-Fen, Au JL-S. Binding of taxol to plastic and glass containers and protein under in vitro conditions. J Pharm Sci 1996 Jan;85(1):29-31.
75. Venkataramanan R, Burckart GJ, Ptachcinski RJ, Blaha R, Logue LW, Bahnson A, et al. Leaching of Diethylhxyl Phthalate from Polyvinyl Chloride Bags into Intravenous Cyclosporine Solution. Am J Hosp Pharm 1986 Nov;43:2800-2.
76. Xu QA, Trissel LA, Zhang Y. Paclitaxel Compatibilit y with the IV Express Filter™ Unit. Int J Pharm Compd 1998 May-June;2(3):243-5.
77. Jung SH, Lee T, Kim K, George SL. Admissible two-stage designs for phase II cancer clinical trials. Statistics in medicine 2004;23(4):561-9 doi 10.1002/sim.1600.
