# Supplementary material for: A phase II trial of weekly nab-paclitaxel for progressive and symptomatic desmoid tumors
Source: Nat Commun. 2022 Oct 21;13:6278. doi: 10.1038/s41467-022-33975-6 (PMC9587294; doi:10.1038/s41467-022-33975-6)
Supplement: Supplementary file 2 — Reporting Summary [file 41467_2022_33975_MOESM2_ESM.pdf]

nature portfolio

Corresponding author(s): Javier Martin-Broto

Last updated by author(s): Sep 29, 2022

Reporting Summary

Nature Portfolio wishes to improve the reproducibility of the work that we publish. This form provides structure for consistency and transparency in reporting. For further information on Nature Portfolio policies, see our Editorial Policies and the Editorial Policy Checklist.

Statistics

For all statistical analyses, confirm that the following items are present in the figure legend, table legend, main text, or Methods section.

n/a	Confirmed	
☐	☒	The exact sample size (*n*) for each experimental group/condition, given as a discrete number and unit of measurement
☐	☒	A statement on whether measurements were taken from distinct samples or whether the same sample was measured repeatedly
☐	☒	The statistical test(s) used AND whether they are one- or two-sided<br>*Only common tests should be described solely by name; describe more complex techniques in the Methods section.*
☐	☒	A description of all covariates tested
☐	☒	A description of any assumptions or corrections, such as tests of normality and adjustment for multiple comparisons
☐	☒	A full description of the statistical parameters including central tendency (e.g. means) or other basic estimates (e.g. regression coefficient) AND variation (e.g. standard deviation) or associated estimates of uncertainty (e.g. confidence intervals)
☐	☒	For null hypothesis testing, the test statistic (e.g. *F*, *t*, *r*) with confidence intervals, effect sizes, degrees of freedom and *P* value noted<br>*Give P values as exact values whenever suitable.*
☒	☐	For Bayesian analysis, information on the choice of priors and Markov chain Monte Carlo settings
☐	☒	For hierarchical and complex designs, identification of the appropriate level for tests and full reporting of outcomes
☒	☐	Estimates of effect sizes (e.g. Cohen's *d*, Pearson's *r*), indicating how they were calculated

Our web collection on statistics for biologists contains articles on many of the points above.

Software and code

Policy information about availability of computer code

Data collection	Clinical data was collected using a web-based electronic CRF. Genes expression data was collected from HTG Transcriptome Panel; endpoints were sampled and annotated manually. Data manipulation was carried on through R version 4.2. costumed R code for methods design was implemented with Limma package (https://www.rdocumentation.org/packages/limma/versions/3.28.14). Plotting the data was perpetrated through R built-in packages.
Data analysis	The clinical trial data was analyzed using SPSS v28.0., R Limma Package, R-Ebayes package and oligo package (v1.54.1).

For manuscripts utilizing custom algorithms or software that are central to the research but not yet described in published literature, software must be made available to editors and reviewers. We strongly encourage code deposition in a community repository (e.g. GitHub). See the Nature Portfolio guidelines for submitting code & software for further information.

Data

Policy information about availability of data

All manuscripts must include a data availability statement. This statement should provide the following information, where applicable:

- Accession codes, unique identifiers, or web links for publicly available datasets
- A description of any restrictions on data availability
- For clinical datasets or third party data, please ensure that the statement adheres to our policy

The clinical data (including demographics, diagnosis, treatment, and efficacy information) can be accessed in the Source Data File. The Microarray dataset is available in the ArrayExpress database under accession code E-MTAB-12163 [https://www.ebi.ac.uk/arrayexpress/experiments/E-MTAB-12163]. The clinical data

used for the translational research and raw gene expression data are available in the ArrayExpress database under the access [E-MTAB-12163] [https://www.ebi.ac.uk/arrayexpress/experiments/E-MTAB-12163]. Source data are provided with this paper.

Human research participants

Policy information about studies involving human research participants and Sex and Gender in Research.

Reporting on sex and gender	Participants gave permission on identifying their gender (M/F)
Population characteristics	The clinical trial allowed the participation of patients of both pediatric and adult populations (6 months or older), with a pathologic diagnosis of deep desmoid tumor of extremities, trunk wall or head and neck region. Participants were required to have measurable disease according to RECIST 1.1 criteria and they could have received one previous chemotherapy line if the scheme was methotrexate plus vinca alkaloids. Patients were also sampled according to their response to the drug under study, and with respect to the location of the desmoid tumor (gastric, neck, extremities, thoracic).
Recruitment	Patients were offered the possibility of participating in the clinical trial by principal investigators at routine hospital visits. Clinical investigators were medical oncologists at tertiary Spanish hospitals in charge of managing desmoid tumor patients. Access to trial participation was publicly promoted via the Spanish Sarcoma Research Group (GEIS). No self-selection or any other type of bias that could impact the results were identified.
Ethics oversight	The initial protocol and its subsequent amendments were approved by the "Provincial de Sevilla" Research Ethics Committee (Seville, Spain) as well as by the Spanish Agency of Medicines and Medical Devices (AEMPS).

Note that full information on the approval of the study protocol must also be provided in the manuscript.

Field-specific reporting

Please select the one below that is the best fit for your research. If you are not sure, read the appropriate sections before making your selection.

[x] Life sciences [ ] Behavioural & social sciences [ ] Ecological, evolutionary & environmental sciences

For a reference copy of the document with all sections, see nature.com/documents/nr-reporting-summary-flat.pdf

Life sciences study design

All studies must disclose on these points even when the disclosure is negative.

Sample size	Regarding the clinical trial design, for sample size estimation, a feasible Simon two-stage design was used, selecting error rates alpha equal to 5% and beta equal to 20%. A total of 21 eligible and treated patients was estimated to be included in the first stage. If ≤ 4 patients had radiological and/or clinical response, the trial would be stopped. Otherwise, with > 4 patients with radiological and/or clinical response, the trial will continue to accrue up to 35 eligible and evaluable patients to enter the study. If 12 or more successes were observed in those 35 subjects, it will be concluded that the results of the trial warrant further investigation. The determined sample size was adequate to evaluate the study endpoints. The translational study was conducted with data of 16 patients, comprising 27189 genes.
Data exclusions	All clinical data was analyzed, except the results related to the variation of physical function of patients during the first year from trial enrollment, which will be assessed in a subsequent publication. Translational data was background corrected, quantile normalized via the RMA (Robust Multi-array Average) method from the oligo package (v1.54.1). DE genes were computed through limma package, modelling the design over each of the 16 patients response. Exclusion occurred over genes not differentially expressed (log2fc near 0), and/or genes with p-value for Differential expression >0.05.
Replication	Procedures of replicative nature included desmoid tumor diagnosis confirmation by an expert central pathologist. This second, additional diagnosis was performed to verify the initial diagnosis provided by the local pathologist at the clinical site. In addition, a central expert radiologist revised the radiological responses specified by the local radiologists.
Randomization	No randomization procedure was used in the clinical trial design (for being a single-arm study). Radiological RECIST 1.1 response classification of the patients (response is the covariate) was exploited.
Blinding	This was a single-arm, open label clinical trial. No blinding procedures were used as researchers were aware of all available data (patient ID, treatment allocation, response, genes raw counts).

Reporting for specific materials, systems and methods

We require information from authors about some types of materials, experimental systems and methods used in many studies. Here, indicate whether each material, system or method listed is relevant to your study. If you are not sure if a list item applies to your research, read the appropriate section before selecting a response.

Materials & experimental systems

n/a	Involved in the study
x	Antibodies
x	Eukaryotic cell lines
x	Palaeontology and archaeology
x	Animals and other organisms
	x Clinical data
x	Dual use research of concern

Methods

n/a	Involved in the study
x	ChIP-seq
x	Flow cytometry
x	MRI-based neuroimaging

Clinical data

Policy information about clinical studies

All manuscripts should comply with the ICMJEguidelines for publication of clinical research and a completedCONSORT checklist must be included with all submissions.

Clinical trial registration	NCT03275818
Study protocol	The full clinical trial protocol is publicly available at: https://grupogeis.org/GEIS-39_Abrades_Protocol_V2_220218-clean.pdf
Data collection	Clinical data were entered into a web-based electronic case report form (CRF) by clinical site staff (study coordinators and data entry personnel). The origin of the data was the medical records, charts, and reports available at the clinical sites where patients were treated (8 tertiary Spanish hospitals). Data collection started upon enrollment of the first patient (May 2017).
Outcomes	Primary outcomes included overall response rate (ORR), clinical benefit rate (CBR) and pain improvement. The ORR (confirmed complete response [CR] and partial response [PR]) was measured using RECIST 1.1 criteria. Response criteria were based on the baseline identification of target lesions and radiological assessments (using magnetic resonance imaging - MRI) performed every 3 months. CBR was measured as CR+PR+SD for 3 months with improvement of pain of at least 2 points in the Brief Pain Inventory – Short Form (BPI-SF). Secondary outcomes included patterns of radiological response according to MRI parameters, treatment efficacy measured by the progression-free survival (PFS) rate assessed by median time, variation of symptoms during the first year from trial enrollment (measured with BPI and Analgesic Quantification Algorithm - AQA score), and the safety profile (toxicity classified using NCI-CTCAE 4.0).
